# Integrative Multi-omics and machine learning analysis of sphingolipid-associated molecular stratification identifies diagnostic and prognostic signatures in idiopathic pulmonary fibrosis

**DOI:** 10.1186/s13023-026-04563-4

**Published:** 2026-08-25

**Authors:** Yi Liao, Lingjing Yang, Xiaoshu Liu, Liqing Yang

**Affiliations:** Department of Pulmonary and Critical Care Medicine, School of Medicine, Sichuan Provincial People’s Hospital, University of Electronic Science and Technology of China, Chengdu, China

**Keywords:** Idiopathic pulmonary fibrosis, Sphingolipid, Machine learning, Diagnosis, Prognosis

## Abstract

**Background:**

Emerging evidence suggests that sphingolipid metabolism is involved in respiratory diseases, including idiopathic pulmonary fibrosis (IPF). This study aimed to evaluate the diagnostic and prognostic value of sphingolipid-related genes in IPF and to identify potential sphingolipid-associated biomarkers.

**Methods:**

Sphingolipid-related genes were obtained from the GeneCards database. Non-negative matrix factorization (NMF) was performed to identify molecular clusters and differentially expressed genes (DEGs). High-dimensional weighted gene co-expression network analysis (hdWGCNA) was used to determine fibroblast-associated genes. The intersecting genes were used to construct diagnostic and prognostic models using multiple machine learning algorithms. Hub genes were identified from the overlap between the diagnostic and prognostic signatures. In silico gene knockout was conducted using scTenifoldKnk. Functional validation was performed using Cell Counting Kit-8 (CCK-8) assay, wound healing assay, quantitative real-time polymerase chain reaction (RT-qPCR), and western blotting (WB).

**Results:**

Three molecular clusters with significant prognostic differences were identified. hdWGCNA revealed 60 key fibroblast-associated genes. Diagnostic and prognostic models constructed from these genes showed promising diagnostic and prognostic utility across independent cohorts, while the prognostic model exhibited some degree of cohort-dependent variability. CCDC80 was identified as a hub gene associated with sphingolipid-defined molecular heterogeneity and fibroblast-related transcriptional programs. scTenifoldKnk analysis indicated that simulated CCDC80 knockout affected fibrosis-related signaling pathways. In vitro experiments demonstrated that CCDC80 knockdown attenuated fibroblast proliferation, migration, and fibrotic activation.

**Conclusion:**

This study identified sphingolipid-related molecular signatures with potential diagnostic and prognostic value in IPF and highlighted CCDC80 as a candidate profibrotic regulator. Further validation in larger and clinically standardized cohorts is required before clinical translation.

**Clinical trial number:**

Not applicable.

**Supplementary Information:**

The online version contains supplementary material available at https://doi.org/10.1186/s13023-026-04563-4.

## Background

Idiopathic pulmonary fibrosis (IPF) was once regarded as a relatively uncommon yet highly debilitating form of interstitial lung disease, characterized by relentless fibrotic remodeling of the pulmonary parenchyma. Epidemiologically, IPF predominantly affects older adults, with a typical age at onset of 65–70 years, and shows a clear male predominance [1]. The incidence of this disease rises progressively with advancing age, and the global disease burden continues to expand. This upward trend is likely driven by population aging, improved clinical awareness, and advances in diagnostic technologies [1]. A meta-analysis published in 2025 reported that the global annual incidence of this disease is approximately 5.8 cases per 100,000 individuals, with a prevalence of about 17.7 per 100,000 [2]. Substantial geographic variation was observed, with higher rates in North America and comparatively lower rates in Asian populations [2]. In several regions and metropolitan areas of China, IPF is no longer regarded as a rare disease but is increasingly recognized as a relatively common clinical entity [3]. Despite advances in antifibrotic treatment, IPF continues to impose a severe global health burden, with a median post-diagnosis survival of merely 2–3 years [1]. This highlights the critical need for improved tools for early diagnosis and prognostic stratification.

An increasing body of evidence supports the notion that disrupted phospholipid metabolism plays a role in accelerating the progression of IPF. Sphingolipids are biologically active lipid components of cell membranes that regulate diverse signaling networks, including profibrotic pathways such as the transforming growth factor-β (TGF-β) axis [4]. Significantly increased levels of the bioactive sphingolipid mediator sphingosine-1-phosphate (S1P) have been detected in the lungs of IPF patients and in relevant animal models. The enzyme responsible for its production, sphingosine kinase 1 (SphK1), is upregulated in idiopathic pulmonary fibrosis and correlates with worsening pulmonary function and poorer survival outcomes [5]. Ceramide, another critical sphingolipid mediator, plays a proapoptotic and profibrotic role in the pulmonary microenvironment. Aberrant ceramide accumulation triggers alveolar epithelial damage, inflammatory responses, and extracellular matrix deposition, ultimately driving fibrotic progression [6]. Importantly, genetic inhibition of acid sphingomyelinase, a key enzyme involved in ceramide generation, markedly attenuated bleomycin-induced lung inflammation and fibrosis in mice, supporting the therapeutic potential of targeting the sphingomyelin/ceramide pathway [7]. Collectively, these observations suggest that aberrant sphingolipid metabolism is mechanistically involved in IPF development, supporting its potential for biomarker identification and targeted therapeutic strategies.

In parallel with biochemical discoveries, the last decade has seen substantial advances in complex disease genomics and genetic epidemiology. Innovative statistical and machine learning frameworks now enable fine-mapping of causal variants and functional interpretation through integration with extensive functional genomics resources, with growing applications in respiratory diseases such as lung cancer [8] and interstitial lung disorders [9]. Machine learning-based strategies have also been successfully applied to other biological and clinical datasets, including spectroscopic fusion data for disease classification and transcriptomic biomarker modeling for cardiovascular diagnosis, further supporting their utility in high-dimensional biological data interpretation and biomarker discovery [10, 11]. IPF represents a paradigm of a complex disorder with a strong genetic contribution, with the well-characterized promoter variant of the *MUC5B* gene constituting the most influential genetic risk factor identified to date [12]. In addition, numerous susceptibility genes have been identified, including telomere-related genes and surfactant protein genes, indicating that genetic factors play a substantial role in the development of IPF [13]. On the basis of these discoveries, the integration of genomics with machine learning methodologies is being actively leveraged to enhance diagnostic accuracy and prognostic stratification in IPF [14]. In this context, advanced genomic integration and computational modeling approaches were employed to establish sphingolipid-centered diagnostic and prognostic frameworks for IPF, with the aim of enhancing risk stratification, informing precision treatment, and identifying therapeutically actionable targets supported by experimental evidence.

## Materials and methods

### Data acquisition and preprocessing

Transcriptomic datasets associated with idiopathic pulmonary fibrosis (IPF) were obtained from the Gene Expression Omnibus (GEO) database, including GSE47460, GSE32537, GSE53845, and GSE150910, which contained lung tissue samples from patients with IPF and matched controls. In addition, GSE70866 consisted of bronchoalveolar lavage fluid (BALF) samples, whereas GSE28042, GSE27957, and GSE93606 were derived from peripheral blood. These four cohorts provided complete prognostic information, including follow-up time and survival status. Only datasets with sample sizes exceeding 40 and with clearly defined diagnostic criteria for IPF or appropriate control selection were included. Detailed baseline characteristics of the included datasets are presented in Supplementary Table 1. Raw expression data were downloaded and preprocessed using the limma package [15] in R. Probe identifiers were converted to corresponding gene symbols according to platform-specific annotation files, and expression values from multiple probes mapping to the same gene were averaged. Subsequently, all datasets were normalized and log2-transformed using the formula log2(x + 1) to reduce technical variability and enhance comparability across cohorts. Sphingolipid-associated genes were obtained from the GeneCards database using “sphingolipid” as the search term. To improve biological specificity and reduce weakly associated entries, genes with a GeneCards relevance score > 7 were retained. The relevance score reflects the strength of association between a gene and the queried term based on integrated GeneCards annotation and supporting evidence.

### Non-negative matrix factorization analysis

Non-negative matrix factorization (NMF) [16] was performed using the expression matrix restricted to the GeneCards-derived sphingolipid-associated genes with relevance score > 7 in the GSE70866 BALF cohort. After removing duplicated or undetectable genes, the remaining sphingolipid-associated genes were used as the input features for NMF-based clustering. Matrix decomposition was performed using the NMF package in R with the Brunet algorithm. To determine the optimal number of clusters, factorization ranks ranging from 2 to 10 were evaluated, with 10 independent runs conducted for each rank. A fixed random seed was applied to ensure reproducibility. Model stability was assessed using cophenetic correlation coefficients, and consensus clustering results were visualized using consensus heatmaps. The optimal number of clusters was determined based on the trends of cophenetic coefficients and the patterns observed in the consensus matrices.

### Differential expression analysis between molecular subtypes

A linear modeling framework with empirical Bayes moderation was applied to perform pairwise comparisons between subtypes. Differentially expressed genes (DEGs) were identified using thresholds of |log₂ fold change| > 0.585 and false discovery rate–adjusted P-value < 0.05, and significant genes from all comparisons were integrated for downstream analyses.

### Single-sample gene set enrichment analysis of immune cell infiltration

Single-sample gene set enrichment analysis (ssGSEA) [17] was applied to the GSE70866 transcriptomic dataset to estimate the relative enrichment levels of immune-related signatures. The ssGSEA algorithm was implemented using the “ssgsea” method in the Gene Set Variation Analysis (GSVA) package [18] with Gaussian kernel estimation. Absolute ranking was performed to each immune-related gene set within individual samples to calculate enrichment scores. These scores were subsequently normalized to a 0–1 scale to quantify the relative abundance of immune cell populations.

### High-dimensional weighted gene co-expression network analysis

Single-cell RNA sequencing data were obtained from the previously published dataset GSE128033 [19]. Data preprocessing, quality control, and normalization were performed according to the established pipeline described in our prior study [20]. The dataset comprised eight samples from patients with IPF and nine samples from healthy controls. High-dimensional weighted gene co-expression network analysis (hdWGCNA) [21] was performed on single-cell RNA sequencing data to identify gene co-expression modules specifically within fibroblast populations. Fibroblast subsets were selected based on cell-type annotations derived from Seurat [22] clustering results. Genes expressed in at least 5% of fibroblast cells were retained using a fraction-based filtering strategy. To enhance network stability and reduce technical noise, metacells were constructed by aggregating transcriptionally similar fibroblast cells according to cell type and sample group information. K-nearest neighbor clustering was conducted using Harmony-corrected embeddings with a neighborhood size of 25. Metacell expression profiles were subsequently normalized and used as input for hdWGCNA. Normalized RNA expression data from fibroblast populations were extracted for network construction. Soft-thresholding powers were systematically evaluated using a signed network topology, and the optimal value was selected based on scale-free topology criteria. Gene co-expression networks were then constructed, and topological overlap matrices were calculated to identify gene modules through hierarchical clustering. Module eigengenes were computed to summarize the expression patterns of each module. Eigengene-based connectivity (kME) values were calculated to assess intramodular connectivity, and hub genes were identified accordingly. Module-specific gene sets were further used to calculate expression scores at the single-cell level using a Seurat-based scoring approach. Inter-module correlations and associations between modules and cell types were subsequently evaluated. Cell–cell communication analysis was conducted using the CellChat package based on normalized single-cell RNA sequencing data and curated human ligand–receptor interaction databases. Communication probabilities were calculated after identifying overexpressed genes and interactions, and aggregated networks were constructed to characterize the strengths and patterns of intercellular signaling across different cell types.

### Integrated machine learning framework for diagnostic and prognostic modeling

Nested cross-validation was not applied in the original model-selection procedure because the study design prioritized independent external validation across multiple GEO cohorts. To reduce overfitting risk, we adopted the following strategies: dimensionality reduction before modeling, training-only feature selection, internal cross-validation for parameter tuning, exclusion of unstable models with very small feature sets, use of parsimonious signatures when performance differences were minimal, and final assessment in independent validation cohorts that were not involved in any stage of model construction. To further improve reproducibility, the computational workflow was provided as Supplementary Data Sheet 1. This file contains the cleaned and annotated R scripts for both diagnostic and prognostic machine learning analyses. An integrated machine learning framework was established to develop both diagnostic classifiers and prognostic prediction models. For diagnostic analysis, twelve algorithms—including least absolute shrinkage and selection operator (LASSO), Ridge regression, elastic net (Enet), stepwise generalized linear models (Stepglm), support vector machines (SVM), gradient boosting–based generalized linear models (glmBoost), linear discriminant analysis (LDA), partial least squares regression for generalized linear models (plsRglm), random forest (RF), gradient boosting machines (GBM), extreme gradient boosting (XGBoost), and Naive Bayes (NB)—were incorporated into a two-stage pipeline that integrated feature selection and model construction, resulting in 113 distinct algorithmic combinations. For prognostic analysis, survival-oriented learners, including Cox proportional hazards regression (CoxPH), LASSO-Cox, Enet-Cox, CoxBoost, random survival forest (RSF), gradient boosting–based survival models, partial least squares Cox regression (plsRcox), and survival support vector machines (survivalsvm), together with their corresponding combinations, were evaluated using a comparable two-stage strategy, generating 101 algorithmic combinations. During the pre-training phase, variable selection was independently conducted for each candidate feature-selection method to derive reduced feature sets, thereby minimizing redundant computations across different model combinations. Each final model was subsequently trained using the corresponding selected features, and models retaining fewer predictors than a predefined minimum threshold were excluded to reduce instability and overfitting. In diagnostic modeling, trained classifiers generated sample-level predicted probabilities for downstream evaluation. In prognostic modeling, risk scores were calculated as linear predictors derived from fitted survival models. Model performance was quantified using the area under the receiver operating characteristic curve (AUC) for diagnostic models and the concordance index (C-index) for prognostic models. The performances of all algorithmic combinations were summarized in integrated evaluation matrices to facilitate optimal model selection.

### In silico gene knockdown analysis

In silico gene knockdown analysis was performed using the scTenifoldKnk package [23] to systematically evaluate the regulatory roles of candidate genes. Highly variable genes were identified using a variance-stabilizing transformation approach, and expression profiles of each target gene together with highly variable genes were retained for the construction of gene regulatory networks. Virtual knockout simulations were conducted by computationally silencing individual target genes, followed by reconstruction of perturbed networks. Quality control procedures were applied to remove low-quality cells and minimize the influence of mitochondrial gene expression. Multiple subsampled gene regulatory networks were generated to enhance analytical robustness, and differential regulatory effects induced by virtual gene perturbation were quantitatively assessed. Genes exhibiting significant regulatory alterations after in silico knockdown were subsequently identified and subjected to downstream functional analyses.

## Functional enrichment analysis

Functional enrichment analyses were performed using the clusterProfiler package [24, 25] in R. Gene Ontology (GO) [26], Kyoto Encyclopedia of Genes and Genomes (KEGG) [27], and Reactome pathway enrichment analyses [28] were subsequently conducted to explore the biological functions and signaling pathways associated with the identified genes.

### Cell culture

Human lung fibroblasts (Product ID: CP-H011) were obtained from Procell Co., Ltd. (Wuhan, China) and cultured in Dulbecco’s modified Eagle’s medium (DMEM) supplemented with 10% fetal bovine serum (FBS) at 37 °C in a humidified atmosphere containing 5% CO₂. Small interfering RNAs targeting CCDC80 (siCCDC80) and negative control siRNA (siCtrl) were synthesized by Tsingke Biotechnology Co., Ltd. After 24 h of transfection, cells were incubated in serum-reduced medium (0.5–1% FBS) for 12 h and subsequently stimulated with recombinant human transforming growth factor-β1 (TGF-β1; HumanBiomics) at a final concentration of 2.5 ng/mL for 24–48 h. Cells were divided into three groups: (1) siCtrl group; (2) siCtrl + TGF-β1 group; and (3) siCCDC80 + TGF-β1 group. Following treatment, cell proliferation and migration were evaluated using the Cell Counting Kit-8 (CCK-8) assay and wound healing assay, respectively. All experiments were performed in triplicate and repeated at least three times independently.

### Cell proliferation assay using CCK-8

Cell viability was assessed using the CCK-8 assay. Cells were digested with 0.25% trypsin–EDTA, resuspended in complete medium, and seeded into 96-well plates at a density of approximately 1,000 cells per well. After incubation at 37 °C with 5% CO₂ for 24–96 h, 10 µL of CCK-8 solution was added to each well, followed by incubation for 2 h. The absorbance was measured at 450 nm using a microplate reader, and cell viability was calculated according to the manufacturer’s instructions.

### Wound healing assay

Cell migration was evaluated using a wound healing assay. Cells were seeded into six-well plates at a density of approximately 1 × 10⁶ cells per well and cultured until reaching near confluence. A linear scratch was generated using a sterile 200-µL pipette tip, followed by gentle washing with phosphate-buffered saline to remove detached cells. Cells were then cultured in medium containing 2% fetal bovine serum, and images were captured at 0 h and at indicated time points using an inverted microscope. The wound closure rate was calculated based on changes in the scratched area to assess migratory capacity.

### Quantitative real-time polymerase chain reaction analysis

Gene expression levels were quantified using real-time quantitative polymerase chain reaction (RT-qPCR). Total RNA was extracted using TRIzol reagent, and RNA purity and concentration were assessed spectrophotometrically. Complementary DNA (cDNA) was synthesized using a commercial reverse transcription kit with genomic DNA removal. RT-qPCR was performed using a SYBR Green–based master mix on a real-time PCR system under standard cycling conditions, followed by melt-curve analysis to confirm amplification specificity. Relative gene expression levels were calculated using comparative quantification methods according to the manufacturer’s instructions. Primer sequences are provided in Supplementary Table 2.

### Western blotting

Cells were lysed in radioimmunoprecipitation assay (RIPA) buffer supplemented with protease and phosphatase inhibitors, and protein concentrations were determined using a bicinchoninic acid (BCA) assay. Equal amounts of protein were separated by sodium dodecyl sulfate–polyacrylamide gel electrophoresis (SDS–PAGE) and transferred onto polyvinylidene fluoride (PVDF) membranes. After blocking with 5% non-fat milk in Tris-buffered saline with Tween 20 (TBST) for 1 h at room temperature, the membranes were incubated overnight at 4 °C with primary antibodies against alpha-smooth muscle actin (α-SMA) (Proteintech, 14395-1-AP, 1:1000), CCDC80 (Aviva Systems Biology, ARP66332_P050, 1:500), and GAPDH (Thermo Fisher Scientific, A300-642 A-T, 1:5000). After washing, membranes were incubated with appropriate infrared dye–conjugated secondary antibodies (LI-COR Biosciences, 1:10,000) for 1 h at room temperature. Target protein expression levels were normalized to GAPDH.

### Statistical analysis

All statistical analyses were performed using R software (version 4.3.1). Principal component analysis (PCA) was conducted on scaled gene expression data using the prcomp function, and the first three principal components were extracted for three-dimensional visualization. Correlations between variables were evaluated using Spearman’s rank correlation analysis. Data distribution and variance homogeneity were examined prior to statistical testing. For normally distributed data with equal variances, parametric tests were applied, including independent Student’s t-tests for two-group comparisons and one-way analysis of variance (ANOVA) for multiple-group comparisons. When parametric assumptions were not satisfied, nonparametric methods were used, including the Wilcoxon rank-sum test for two-group comparisons and the Kruskal–Wallis test for multiple-group comparisons. Survival differences between groups were assessed using Kaplan–Meier survival analysis in conjunction with the log-rank test, and the optimal cutoff value for survival stratification was determined using the surv_cutpoint() function. Cross-cohort expression validation was performed using random-effects meta-analysis based on standardized mean differences (SMDs), and heterogeneity was assessed using Cochran’s Q test and the I² statistic. All statistical tests were two-sided, and a P-value < 0.05 was considered statistically significant.

## Results

### Molecular classification defines prognostic and clinical heterogeneity in IPF

**Fig. 1:**
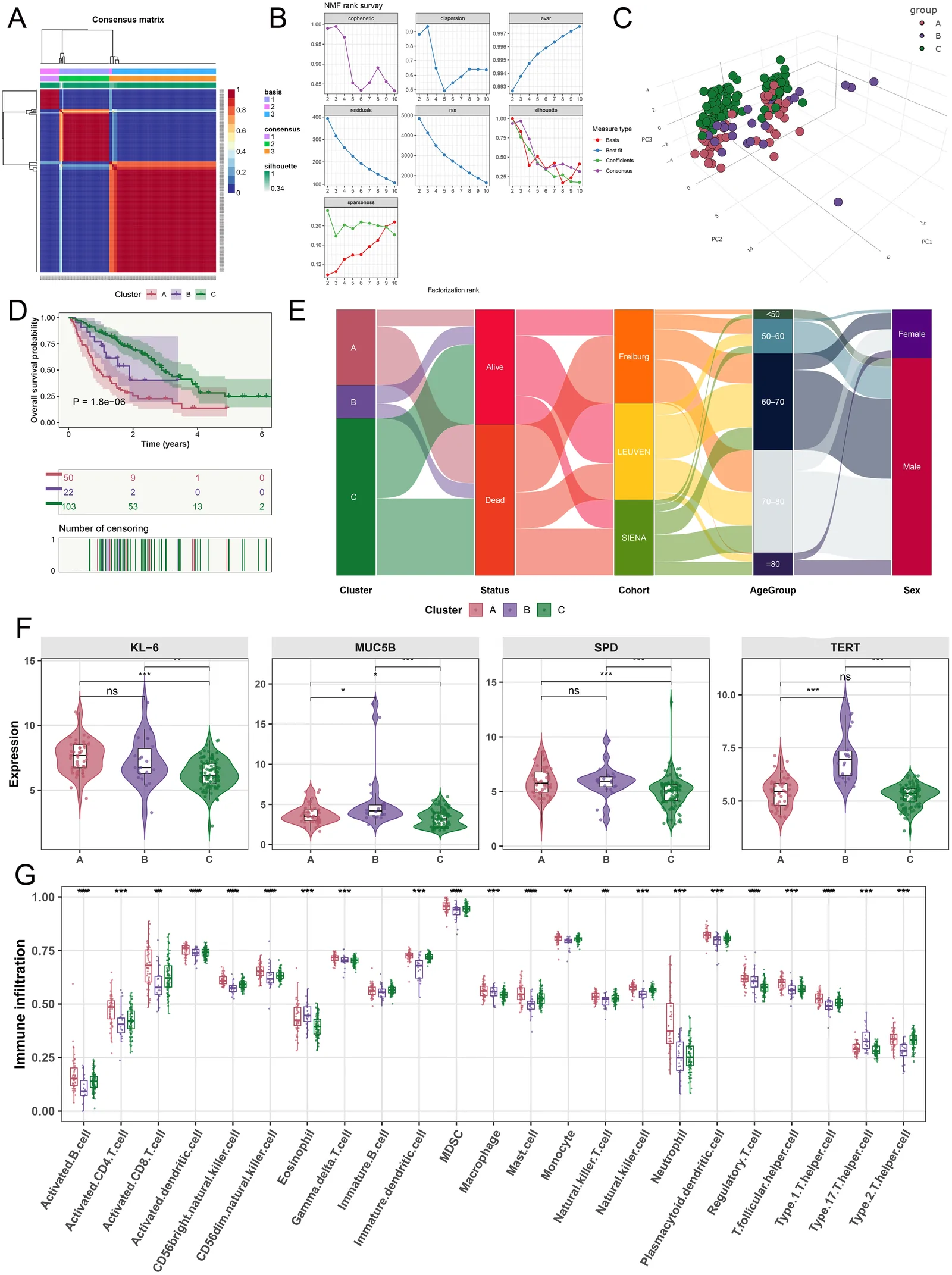
Molecular subtyping of IPF based on sphingolipid-related genes. (**A**) Consensus clustering heatmap showing three molecular subtypes. (**B**) Rank survey for determining the optimal cluster number. (**C**) Three-dimensional PCA plot of the three subtypes. (**D**) Kaplan–Meier survival curves among molecular subtypes. (**E**) Alluvial plot showing the distribution of subtypes according to clinical features. (**F**) Expression of fibrosis-related biomarkers among subtypes. (**G**) Immune infiltration differences among subtypes. Statistical significance is indicated as follows: * *P* < 0.05, ** *P* < 0.01, *** *P* < 0.001

Based on the GeneCards search using “sphingolipid” as the keyword, 52 genes (Supplementary Table 3) with a relevance score > 7 were retained as the sphingolipid-associated gene set for downstream analysis. Unsupervised clustering analysis was conducted using the NMF algorithm to classify patients into distinct molecular subtypes. The consensus matrix revealed robust clustering stability at k = 3, characterized by high intra-cluster correlation and clear inter-cluster separation (Fig. 1A). Rank survey analysis, including cophenetic coefficient, dispersion, and silhouette width, consistently supported three as the optimal cluster number (Fig. 1B). Three-dimensional PCA further confirmed that the three subtypes were distributed in distinct regions of the reduced-dimensional space, indicating substantial transcriptomic heterogeneity among subgroups (Fig. 1C). Kaplan–Meier survival analysis demonstrated significant differences in overall survival among the three subtypes, with subtype C exhibiting the most favorable prognosis and subtype A showing the poorest survival outcome (log-rank P-value < 0.001; Fig. 1D). Sankey diagram analysis illustrated the relationships between molecular subtypes and clinical variables, including survival status, cohort source, age group, and sex, revealing distinct clinical distribution patterns across subgroups (Fig. 1E). Comparative analysis of fibrosis-associated biomarkers [29–32] showed significant subtype-specific expression differences, with KL-6, MUC5B, SPD, and TERT displaying heterogeneous expression profiles among subtypes (Fig. 1F). Furthermore, immune infiltration analysis revealed marked differences in immune cell composition, with subtype C characterized by enhanced adaptive immune cell infiltration, whereas subtype A exhibited relatively lower immune activity and increased innate immune components (Fig. 1G). Collectively, these findings indicate that the identified molecular subtypes represent biologically and clinically distinct entities with divergent prognostic and immunological characteristics. The complete lists of DEGs across all clusters are provided in Supplementary Table 4.

### Fibroblast-specific co-expression networks revealed by hdWGCNA

**Fig. 2:**
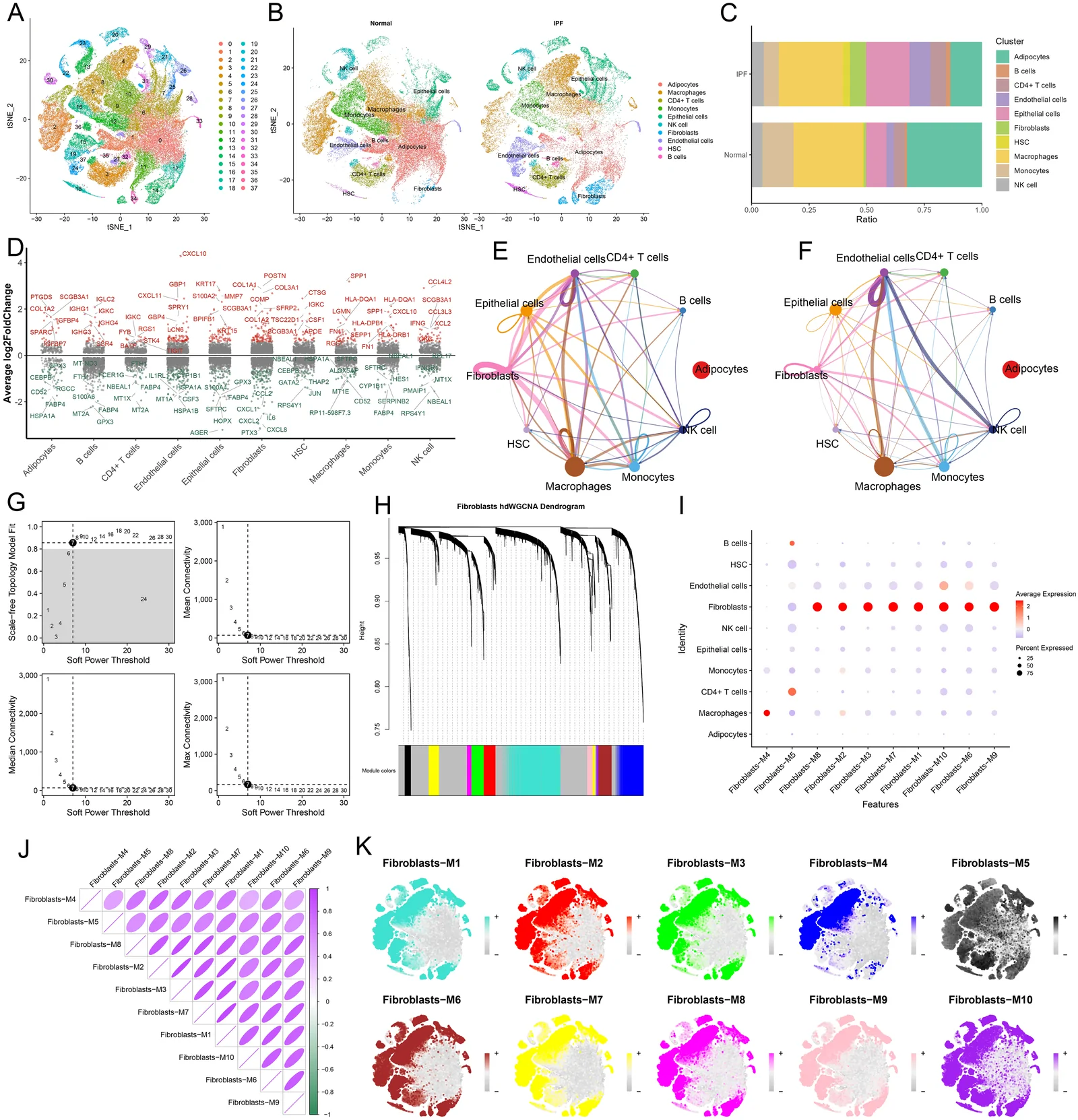
Single-cell transcriptomic profiling and fibroblast-specific hdWGCNA analysis. (**A**) t-SNE visualization of single-cell clusters. (**B**) Annotation of major cell populations. (**C**) Cell-type proportions in control and IPF samples. (**D**) Representative differentially expressed genes across cell types. (**E**–**F**) CellChat analysis of intercellular interaction number (**E**) and interaction strength (**F**). (**G**) Selection of the soft-thresholding power for hdWGCNA. (**H**) Fibroblast co-expression modules identified by hdWGCNA. (**I**) Module activity across fibroblast clusters. (**J**) Correlations among module eigengenes. (**K**) t-SNE visualization of module hub gene expression

Single-cell transcriptomic profiling identified multiple distinct cellular populations in lung tissues, as visualized by t-SNE analysis (Fig. 2A). Cell-type annotation revealed major immune and stromal populations, including fibroblasts, epithelial cells, macrophages, endothelial cells, and lymphocytes (Fig. 2B). Comparative analysis showed marked alterations in cellular composition between normal and IPF samples, with a pronounced expansion of fibroblasts in IPF tissues (Fig. 2C). Differential expression analysis across major cell types identified distinct transcriptional signatures, with fibroblasts exhibiting prominent upregulation of extracellular matrix–related and inflammatory-associated genes (Fig. 2D). Cell–cell communication analysis further indicated that fibroblasts actively interacted with macrophages, endothelial cells, and epithelial cells, forming dense intercellular signaling networks in both interaction number and strength (Fig. 2E–F). To investigate fibroblast-specific regulatory programs, high-dimensional weighted gene co-expression network analysis (hdWGCNA) was performed. Based on scale-free topology criteria, a soft-thresholding power of 7 was selected for network construction (Fig. 2G). Hierarchical clustering of topological overlap matrices identified multiple robust gene modules within fibroblasts (Fig. 2H). These modules exhibited distinct expression patterns across cell types, with several modules showing strong fibroblast-specific enrichment (Fig. 2I). Correlation analysis among module eigengenes revealed coordinated regulatory relationships between fibroblast modules, indicating organized transcriptional programs (Fig. 2J). Projection of module expression onto t-SNE embeddings further demonstrated heterogeneous spatial distributions of individual modules within fibroblast populations (Fig. 2K). For each fibroblast-specific module, the top 25 hub genes were selected based on kME values and intramodular connectivity, and were summarized in Supplementary Table 5. Genes overlapping between cluster-specific DEGs and hdWGCNA-identified hub genes were extracted and used for downstream analyses, as summarized in Supplementary Table 6.

### Development and validation of a robust machine learning-based diagnostic model

**Fig. 3:**
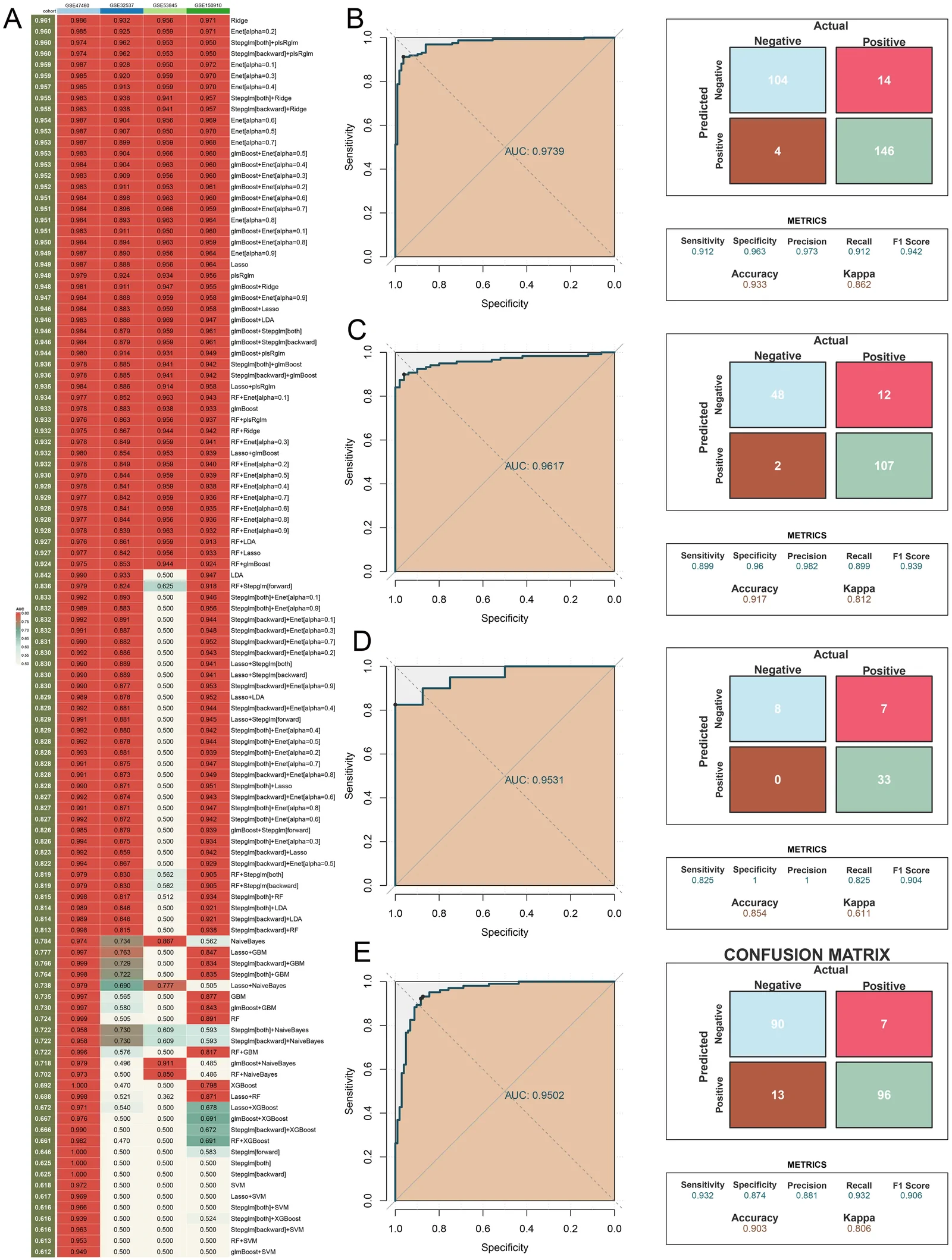
Construction and validation of the diagnostic model. (**A**) AUC comparison of 113 diagnostic algorithm combinations. (**B**–**E**) ROC curves and confusion matrices of the selected diagnostic model in GSE47460, GSE32537, GSE53845, and GSE150910

Based on the intersection genes obtained from differential expression analysis and hdWGCNA, an integrated machine learning framework was implemented to develop diagnostic classifiers. A total of 113 algorithm combinations were systematically evaluated in the training cohort GSE47460 (Fig. 3A). Among all candidate models, the Stepglm (both) + partial least squares regression for generalized linear models (plsRglm) algorithm was selected as the selected classifier. Although its mean area under the receiver operating characteristic curve (AUC) ranked second among all combinations, the difference from the top-ranked model was minimal (ΔAUC = 0.001). Notably, this model required only 20 predictive genes, representing the smallest feature set, thereby providing an optimal balance between diagnostic performance and model simplicity. In the training dataset GSE47460, the selected model achieved an AUC of 0.9739, with an accuracy of 93.3%, sensitivity of 91.2%, and specificity of 96.3%, indicating excellent discriminatory capability between idiopathic pulmonary fibrosis and normal samples. The generalizability of the Stepglm (both) + plsRglm classifier was further evaluated in three independent validation cohorts, including GSE32537, GSE53845, and GSE150910 (Fig. 3B–E). In GSE32537, the model yielded an AUC of 0.9617 with an accuracy of 91.7%. In GSE53845, an AUC of 0.9531 and an accuracy of 85.4% were achieved, accompanied by perfect specificity. In GSE150910, the classifier demonstrated an AUC of 0.9502 and an accuracy of 90.3%, further confirming its robust diagnostic performance. Collectively, these results suggest that the Stepglm (both) + plsRglm model, constructed using a minimal set of intersection genes, exhibits high accuracy and strong generalization ability across multiple independent cohorts, highlighting its potential clinical applicability for IPF diagnosis.

### Prognostic performance of the machine learning-based risk model

**Fig. 4:**
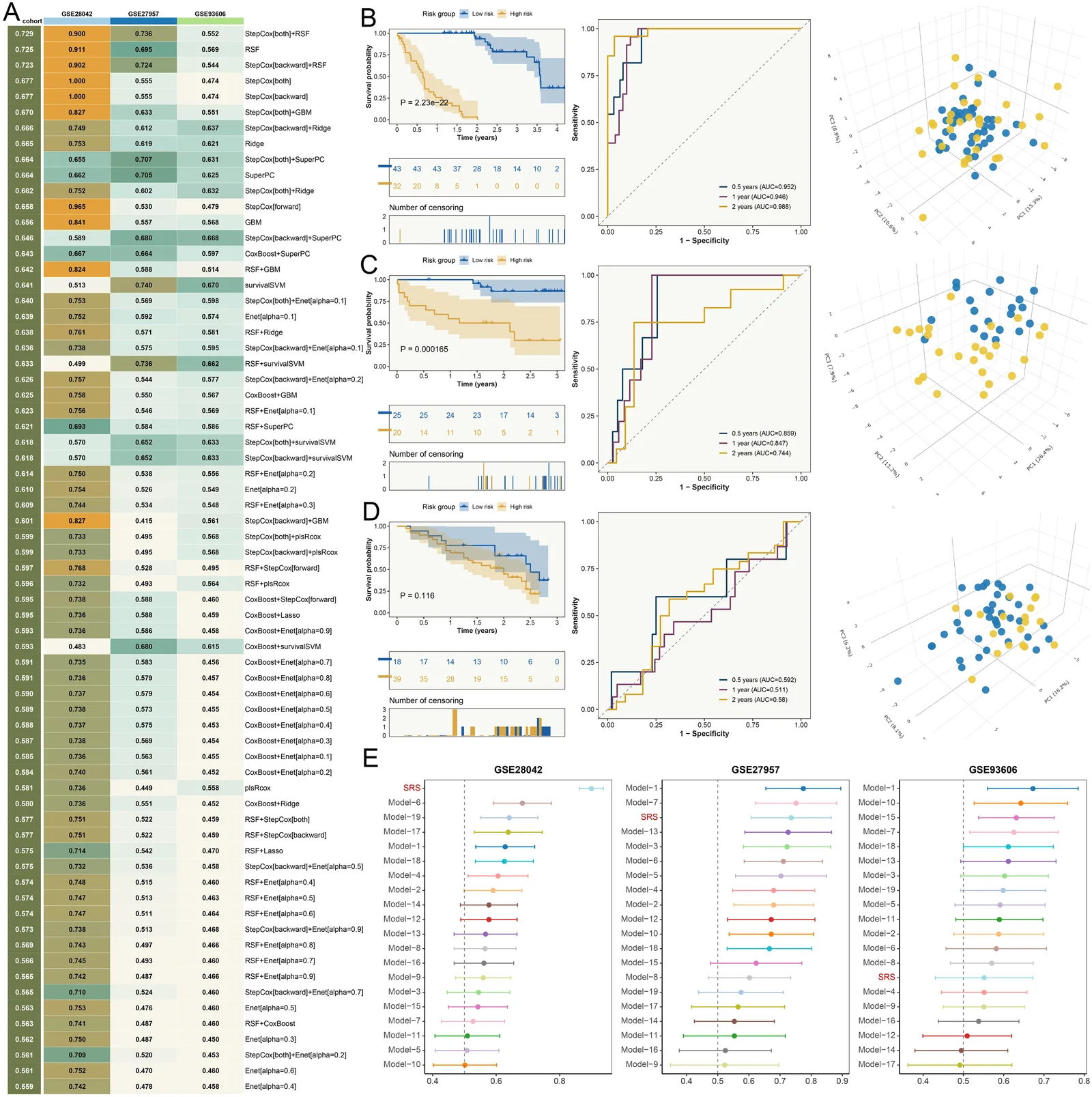
Prognostic performance of the machine learning-based risk model. (**A**) C-index comparison of 101 prognostic algorithm combinations. (**B**–**D**) Kaplan–Meier survival curves, time-dependent ROC curves, and three-dimensional PCA plots in GSE28042, GSE27957, and GSE93606. (**E**) Comparison of the proposed risk model with previously published prognostic signatures

Based on the intersection genes derived from differential expression analysis and hdWGCNA, a machine learning–based prognostic risk model was constructed and evaluated in one training cohort (GSE28042) and two independent validation cohorts (GSE27957 and GSE93606) (Fig. 4A). The optimal cutoff of the Sphingolipid related risk score (SRS) was determined using the surv_cutpoint function, and patients were stratified into high- and low-risk groups accordingly. In GSE28042, Kaplan–Meier analysis revealed a highly significant survival difference between risk groups, with the high-risk group showing markedly poorer outcomes (Fig. 4B). The model also demonstrated strong time-dependent predictive accuracy across follow-up time points (Fig. 4B). In GSE27957, the survival separation remained significant (Fig. 4C), with consistently robust time-dependent ROC performance (Fig. 4C). In GSE93606, the high-risk group showed an unfavorable survival trend, although the difference was not statistically significant (Fig. 4D). PCA further indicated distinct transcriptomic patterns between risk groups in the training cohort and a consistent separation trend in validation cohorts (Fig. 4B–D, right). Finally, when benchmarked against previously published prognostic signatures (Supplementary Table 7), the proposed model achieved a relatively high concordance index across cohorts (Fig. 4E). However, because survival separation in GSE93606 did not reach statistical significance, the prognostic performance of this model should be interpreted cautiously, and its generalizability across different blood-derived cohorts requires further validation.

### Identification and functional characterization of hub genes

**Fig. 5:**
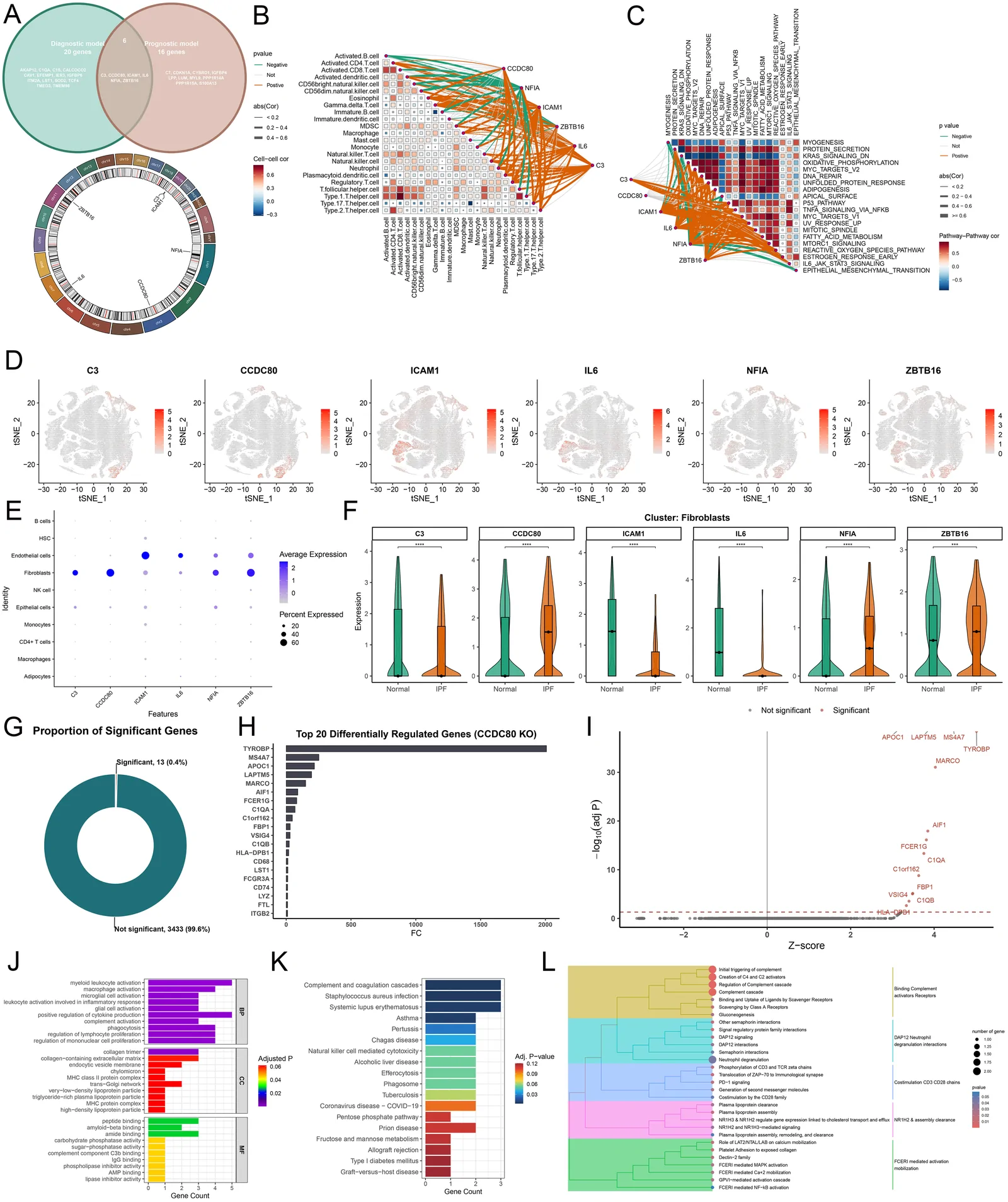
Integrated identification and functional annotation of hub genes. (**A**) Overlap between diagnostic and prognostic model genes and chromosomal distribution of hub genes. (**B**) Correlations between hub genes and immune infiltration. (**C**) Associations between hub genes and hallmark pathways. (**D**–**E**) Cellular distribution of hub genes in single-cell data. (**F**) Differential expression of hub genes in control and IPF fibroblasts. (**G**–**I**) Gene perturbation effects after simulated CCDC80 knockdown. (**J**–**L**) GO, KEGG, and Reactome enrichment analyses of significantly perturbed genes

Intersection genes shared by the diagnostic and prognostic models were identified as potential hub genes for subsequent analyses (Fig. 5A), including C3, CCDC80, ICAM1, IL6, NFIA, and ZBTB16, which are located on chromosomes 1, 3, 7, 11, and 19. Correlation analyses were performed to investigate the relationships between hub gene expression, immune cell infiltration, and hallmark signaling pathways. As shown in Fig. 5B, significant associations were observed between hub gene expression and multiple immune cell populations, particularly activated T cells, natural killer cells, macrophages, dendritic cells, and monocytes, with most interactions exhibiting moderate to high positive correlation coefficients, suggesting close associations with immune activation and inflammatory infiltration in IPF. Pathway-level analysis further revealed extensive interactions between hub genes and key biological processes (Fig. 5C), including tumor necrosis factor alpha (TNF-α) signaling via nuclear factor kappa B (NF-κB), interleukin 6–Janus kinase–signal transducer and activator of transcription 3 (IL-6–JAK–STAT3) signaling, MYC targets, reactive oxygen species pathway, epithelial–mesenchymal transition, and fatty acid metabolism, with IL6, ICAM1, and C3 showing especially strong associations with inflammatory and stress-response pathways. The cellular distribution patterns of these hub genes are shown in Fig. 5D, demonstrating preferential expression in fibroblasts for C3, CCDC80, NFIA, and ZBTB16 (Fig. 5E). Moreover, comparative analysis between idiopathic pulmonary fibrosis and control fibroblasts revealed that CCDC80 was significantly upregulated in idiopathic pulmonary fibrosis–derived fibroblasts (Fig. 5F), suggesting a potential role in disease progression and fibrotic remodeling.

### In silico gene perturbation and functional enrichment analysis using scTenifoldKnk

To investigate the regulatory impact of hub genes at the single-cell transcriptomic level, in silico gene perturbation analysis was performed using the scTenifoldKnk framework, which enables virtual gene knockdown through gene regulatory network reconstruction and comparative network analysis. Following virtual knockdown of the target gene CCDC80, differential regulatory changes were systematically evaluated, revealing that a small proportion of genes exhibited significant regulatory alterations, with 13 genes (0.4%) reaching statistical significance, whereas the majority of genes (99.6%) remained unchanged, indicating selective transcriptional reprogramming rather than global disruption of gene expression (Fig. 5G). The top differentially regulated genes, including TYROBP, MS4A7, APOC1, LAPTM5, MARCO, AIF1, FCER1G, C1QA, and C1QB, showed prominent regulatory shifts and were mainly associated with innate immune responses and macrophage activation (Fig. 5H), This finding was further supported by Z-score-based analysis, which demonstrated significant transcriptional changes in multiple immune-related genes (Fig. 5I). To further elucidate the biological consequences of these regulatory alterations, functional enrichment analyses were conducted using GO, KEGG, and Reactome databases, revealing that differentially regulated genes were predominantly enriched in immune- and inflammation-related biological processes, including myeloid leukocyte activation, macrophage activation, inflammatory response, and complement activation, as well as cellular components associated with extracellular matrix organization and major histocompatibility complex class II protein complexes and molecular functions related to peptide binding and immunoglobulin binding (Fig. 5J). Consistently, pathway analyses demonstrated significant enrichment in complement and coagulation cascades, phagosome, efferocytosis, natural killer cell–mediated cytotoxicity, Fc receptor–mediated signaling, neutrophil degranulation, platelet activation, lipid metabolism, and CD3/CD28 costimulation pathways (Fig. 5K–L). Collectively, these results suggest that scTenifoldKnk-based virtual knockdown induces focused and biologically meaningful transcriptional reprogramming, primarily affecting immune regulation, inflammatory responses, and extracellular matrix remodeling, thereby highlighting the potential regulatory role of the hub gene in disease-associated molecular networks.

### Effects of CCDC80 silencing on fibroblast proliferation, migration, and fibrotic activation

**Fig. 6:**
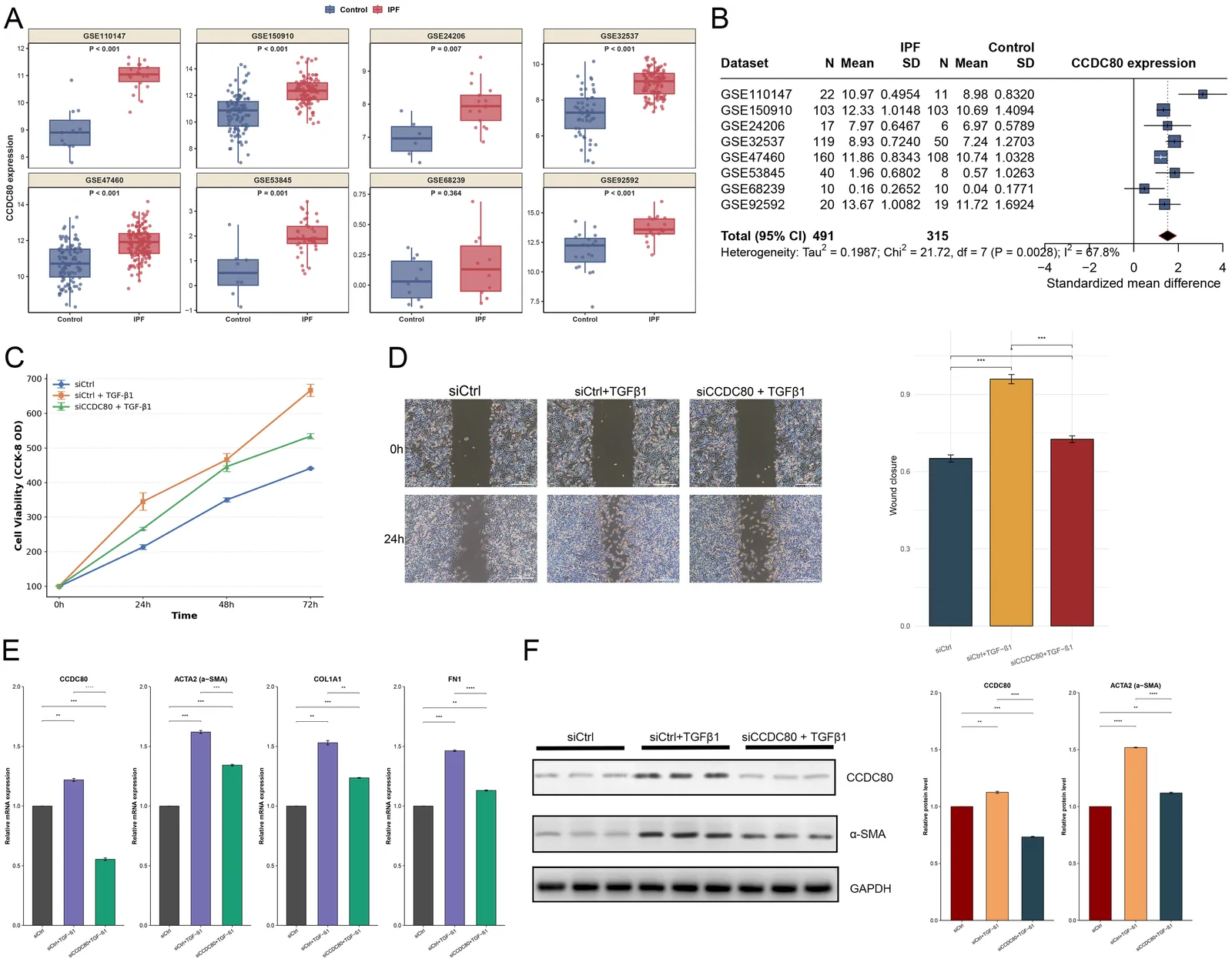
Functional validation of CCDC80 in fibroblasts. (**A**) CCDC80 expression in IPF and control samples across eight independent transcriptomic datasets. Differences were assessed using the Wilcoxon rank-sum test. (**B**) Random-effects meta-analysis of CCDC80 expression differences between IPF and control samples. The standardized mean difference (SMD) was calculated as Hedges’ g, with positive values indicating higher expression in IPF. (**C**) Cell viability assessed by CCK-8 assay in three experimental groups: siCtrl, siCtrl plus TGF-β1, and siCCDC80 plus TGF-β1. (**D**) Wound healing assay comparing migratory capacity among the three groups. (**E**) Quantitative PCR analysis of fibrosis-related gene expression in the three experimental conditions. (**F**) Western blot analysis showing protein expression levels across the three groups

To further validate the disease-related relevance of CCDC80, we examined its expression in eight independent IPF cohorts comprising 491 IPF and 315 control samples. CCDC80 was significantly upregulated in IPF samples in most datasets, whereas GSE68239 showed a positive but non-significant trend (Fig. 6A). Random-effects meta-analysis confirmed that CCDC80 expression was significantly higher in IPF than in controls, with a pooled SMD of 1.5224 (95% CI: 1.1341–1.9107; P-value < 0.0001; Fig. 6B). Cell proliferation analysis using the CCK-8 assay showed that the siCtrl + TGF-β1 group exhibited the highest growth rate, followed by the siCCDC80 + TGF-β1 group, whereas the siCtrl group displayed the lowest proliferative capacity (Fig. 6C). These results indicate that TGF-β1 markedly promoted fibroblast proliferation, which was partially attenuated by CCDC80 knockdown. Wound-healing assay results demonstrated that fibroblast migration was most pronounced in the siCtrl + TGF-β1 group, moderately enhanced in the siCCDC80 + TGF-β1 group, and weakest in the siCtrl group (Fig. 6D). These findings suggest that TGF-β1 significantly stimulated cell migratory activity, while silencing CCDC80 partially suppressed this effect. Furthermore, RT-qPCR and WB analyses demonstrated that CCDC80 expression was significantly reduced in the siCCDC80 group, confirming effective knockdown. In contrast, α-SMA expression was lowest in the siCtrl group, increased in the siCCDC80 + TGF-β1 group, and was highest in the siCtrl + TGF-β1 group (Fig. 6E and F). These results indicate that TGF-β1 induced strong fibrotic activation in fibroblasts, which was partly reversed by CCDC80 knockdown.

## Discussion

In this study, an integrative analytical framework combining transcriptome profiling, hdWGCNA, machine learning modeling, single-cell analysis, and experimental validation was applied to systematically investigate key regulatory mechanisms in IPF. First, NMF was used to define prognostic clusters and identify DEGs associated with survival differences. Subsequently, hdWGCNA was performed to identify fibroblast-related candidate key genes. Diagnostic and prognostic models were then constructed to further prioritize core genes. Through this multi-level strategy, CCDC80 was identified as a central hub gene with significant diagnostic, prognostic, and functional relevance. In vitro experiments further demonstrated that CCDC80 may be involved in fibroblast activation and fibrotic responses. Consistent results from bulk transcriptomic analyses, single-cell data, and cellular experiments collectively support a potential role of CCDC80 in fibrotic progression.

Building on the first step of our analytical framework, the NMF-derived subtypes may provide clinically relevant information for IPF risk stratification. In the BALF-derived GSE70866 cohort, three sphingolipid-related molecular subtypes were identified and showed marked prognostic differences, with subtype A exhibiting the poorest survival and subtype C showing the most favorable outcome. These findings suggest that sphingolipid-related molecular stratification may help distinguish patients with different progression risks. Importantly, these subtypes were not only separated at the transcriptomic level but were also associated with clinically relevant variables, including survival status, cohort source, age group, and sex. The biological features of these subtypes further support their potential clinical significance. Fibrosis-associated biomarkers, including KL-6, MUC5B, SPD, and TERT, displayed subtype-specific expression patterns, indicating that the three subtypes may represent distinct fibrotic molecular states. In addition, immune infiltration analysis revealed extensive differences in both adaptive and innate immune cell populations across subtypes, suggesting that sphingolipid-related molecular stratification may reflect distinct immune microenvironmental patterns in IPF. Therefore, these subtypes may provide a useful framework for identifying high-risk patients, refining follow-up intensity, and exploring subtype-specific biological mechanisms. Meanwhile, the subtype-associated DEGs also provided the basis for the subsequent integration with fibroblast-specific hdWGCNA, linking BALF-derived molecular heterogeneity to fibroblast-centered regulatory programs.

The defining pathological hallmark of IPF is the usual interstitial pneumonia (UIP) pattern, which is characterized by marked spatial heterogeneity in fibrotic involvement, with dense fibrotic lesions interspersed with relatively preserved lung parenchyma [33]. Fibroblastic foci are consistently observed at the interface between fibrotic and less affected regions and represent critical microanatomical niches where myofibroblasts and aberrantly accumulated extracellular matrix converge [33]. Evidence from spatial proteomic studies further indicates that fibroblastic foci possess a quantitatively enriched and compositionally distinct extracellular matrix profile and serve as active sites of collagen biosynthesis. Concurrently, single-cell transcriptomic analyses have provided direct lineage-level evidence supporting fibroblasts and myofibroblasts as the principal cellular effectors of fibrotic remodeling in IPF lungs [34]. In addition to canonical IPF-associated myofibroblasts, multiple stromal cell subpopulations including invasive fibroblast-like cells have been identified, reflecting substantial cellular heterogeneity within the fibrotic niche [34]. Moreover, aberrant epithelial cell populations such as basaloid-like cells located adjacent to fibroblastic foci are spatially aligned with mesenchymal cells, suggesting that epithelial–mesenchymal interactions constitute a fundamental histopathological basis for tissue remodeling in IPF [34]. More refined single cell study further demonstrates that pulmonary fibrosis is not driven by a single fibroblast state but instead results from the coordinated contributions of multiple fibroblast-lineage states residing within distinct spatial microenvironments that collectively promote extracellular matrix expansion [35]. Accordingly, the present study focuses on fibroblast-related candidate core genes with potential regulatory significance in IPF.

There is relatively direct human evidence linking the sphingolipid signaling axis, particularly S1P, to IPF. A study has reported elevated S1P levels in both serum and bronchoalveolar lavage fluid from patients with IPF, and S1P concentrations in bronchoalveolar lavage fluid show significant correlations with pulmonary function parameters [36]. Meanwhile, the key S1P synthetic enzyme SPHK1 is upregulated in IPF lung tissue and is associated with the expression of alpha-smooth muscle actin, vimentin, and type I collagen, indicating a close relationship between the S1P–SPHK1 axis and myofibroblast accumulation together with extracellular matrix deposition [36]. In animal models, increased S1P levels are also observed in murine pulmonary fibrosis, inhibition or knockdown of Sphk1 alleviated bleomycin-induced lung fibrosis, whereas loss of sphingosine-1-phosphate lyase function aggravated fibrotic phenotypes, collectively supporting a causal role of this axis in vivo [5]. At the level of downstream signaling within fibroblasts, in vivo studies showed that fibroblast-specific deletion of Sphk1 confers protection against bleomycin-induced pulmonary fibrosis, while in vitro experiments demonstrated that TGF-β induces SPHK1 activation in human lung fibroblasts and that blockade of SPHK1 reduces YAP1 nuclear translocation, mitochondrial reactive oxygen species production, and the expression of fibronectin and α-SMA, thereby directly linking S1P signaling to myofibroblast differentiation and extracellular matrix production [37]. In addition, S1P receptor subtypes have been shown to modulate profibrotic responses in fibroblasts [38]. Collectively, the convergence of fibroblast-centered pathological features in IPF and the causal involvement of sphingosine-related signaling in driving myofibroblast activation and extracellular matrix deposition provided a strong rationale for prioritizing fibroblast-associated sphingolipid-related genes in the present study.

The major clinical challenge in diagnosing IPF lies in identifying a usual interstitial pneumonia (UIP) pattern within an appropriate clinical context while excluding other known causes of interstitial lung disease. The 2018 official clinical practice guidelines conditionally recommend bronchoalveolar lavage and surgical lung biopsy for cases classified as probable UIP, indeterminate UIP, or alternative diagnosis, underscoring that histopathological evidence remains essential for a subset of patients [39]. Against this background, the use of lung tissue transcriptomic and gene expression profiles to assist in the identification of UIP or IPF has been explored and supported by prospective validation studies. Gene expression signatures derived from transbronchial biopsy samples, particularly when integrated with machine learning approaches, have demonstrated the ability to capture UIP-associated features and provide complementary information to radiological assessment [40]. In the present study, the diagnostic model achieved an area under the curve exceeding 0.95 in both training and validation cohorts, indicating favorable diagnostic performance compared with several previously reported models [41, 42]. This approach may therefore offer quantitative molecular evidence to support clinical decision-making in diagnostically challenging cases or in patients unable to complete standard imaging examinations. Nevertheless, its translational applicability is primarily constrained by tissue acquisition strategies and warrants further validation in multicenter, prospective settings using real-world sampling approaches such as transbronchial or cryobiopsy.

Although the diagnostic classifier showed favorable performance in lung tissue transcriptomic cohorts, its direct use in routine clinical practice remains limited because lung tissue acquisition is invasive and is not required for all patients with suspected IPF. Therefore, the present model should be interpreted as a tissue-derived molecular reference rather than an immediately deployable screening test. A feasible translational strategy would be to convert the selected diagnostic genes into a simplified assay, such as RT-qPCR, targeted RNA sequencing, or a Nanostring-based panel, and then evaluate the classifier score in clinically accessible specimens. BALF may represent a practical intermediate sample type because it reflects the local airway and alveolar microenvironment and has been used for transcriptome-based IPF endotyping with prognostic relevance [43]. Blood-based assays would be more suitable for repeated monitoring, outpatient follow-up, and large-scale risk assessment, as previous studies have shown that peripheral blood gene-expression profiles can predict IPF outcomes [44]. In addition, the clinical feasibility of molecular classifiers in interstitial lung disease has been supported by the Envisia Genomic Classifier, which uses RNA derived from transbronchial biopsy samples to assist UIP/IPF diagnosis [45]. Therefore, future translation of our model should follow a stepwise strategy: first, technical conversion of the signature into a robust low-gene clinical assay; second, validation in BALF, blood, or bronchoscopic samples; and third, prospective evaluation together with high-resolution computed tomography (HRCT) patterns, pulmonary function, and multidisciplinary diagnosis according to current IPF diagnostic guidelines [46]. Such validation is necessary before the classifier can be considered for routine clinical decision-making.

The clinical course of IPF is highly heterogeneous, and reliance solely on the rate of pulmonary function decline or radiological progression often fails to identify in a timely manner patients at high risk. As a result, prognostic biomarkers that are reproducible, minimally invasive, and suitable for dynamic longitudinal monitoring have attracted sustained interest. Peripheral blood transcriptomic profiling has yielded landmark advances in this field. One study proposed a 52-gene signature based on peripheral blood mononuclear cell expression to predict adverse outcomes in IPF, which was subsequently validated in international multicenter cohorts, supporting its cross-cohort reproducibility and prognostic relevance [44]. At the level of disease progression prediction, further evidence indicates that blood-based transcriptomic profiles can also predict fibrotic progression and are linked to specific immune cell-related patterns [47]. Compared with previously published prognostic models, the model developed in the present study demonstrates a clear advantage in predictive performance. Moreover, blood sampling is minimally invasive and repeatable, making it well suited for risk stratification and longitudinal follow-up. Nevertheless, blood transcriptomic signals are susceptible to confounding factors such as infection status, pharmacological treatment, comorbidities, and sampling time points, underscoring the need for further validation in prospective follow-up studies with standardized sampling protocols and treatment-stratified analyses to strengthen clinical applicability.

The present study further identified CCDC80 as a potential biomarker in IPF. From a molecular perspective, CCDC80 is not merely an intracellular protein but contains a signal peptide and can be partially secreted into the extracellular space. Previous study has shown that CCDC80 participates in intracellular signaling and may undergo proteolytic processing, suggesting its potential to influence the tissue microenvironment and cell–matrix interactions [48]. In the context of pulmonary diseases, emerging evidence has implicated CCDC80 in related pathological processes. For example, a comparative transcriptomic analysis across multiple pulmonary arterial hypertension cohorts identified CCDC80 as a commonly dysregulated gene, and in vivo functional validation demonstrated that CCDC80 deletion reduced the expression of COL1A1 and endothelin-1 [49]. In addition, a study in chronic obstructive pulmonary disease reported that WGCNA modules correlated with specific lung macrophage subpopulation proportions were enriched for extracellular matrix organization and collagen metabolism and included CCDC80 among the constituent genes [50]. With respect to IPF, prior work has shown an association between CCDC80 expression and declines in diffusing capacity of the lung for carbon monoxide [51]. Building on these observations, the present study advances the evidence from correlative associations to functional validation. Under TGF-β1 stimulation, CCDC80 expression was upregulated in parallel with α-SMA, whereas silencing CCDC80 partially attenuated fibroblast proliferation, migration, and myofibroblastic phenotypes. These findings suggest that CCDC80 may participate in transforming growth factor beta-driven amplification of fibrotic responses and provide a potential entry point for further mechanistic investigation in IPF. CCDC80 should not be interpreted as a canonical sphingolipid metabolic gene. Instead, it was identified as a fibroblast-associated hub gene linked to sphingolipid-defined molecular heterogeneity. Direct lipidomic or metabolomic evidence is still needed to clarify whether CCDC80 is functionally involved in sphingolipid metabolism.

Several limitations should be acknowledged. First, this study was mainly based on public retrospective transcriptomic datasets, and the proposed diagnostic and prognostic models have not yet been validated in an independent prospective clinical IPF cohort. Although multiple datasets were used for validation, differences in platform, cohort composition, tissue origin, and sampling procedures may still affect model comparability and generalizability. Lung tissue, BALF, peripheral blood mononuclear cells (PBMCs), and whole blood may reflect different biological compartments of IPF; therefore, the proposed signatures should currently be regarded as candidate molecular tools rather than ready-to-use clinical assays. Second, the prognostic model showed potential stratification value, but its performance was not uniformly significant across all external blood-derived validation cohorts, suggesting possible cohort-dependent variability. Further validation in larger, standardized, and prospectively collected blood-based IPF cohorts is needed. Third, the sphingolipid-associated gene set was derived from GeneCards using a relevance score threshold rather than from direct lipidomic or metabolomic profiling. Thus, the identified subtypes and signatures reflect sphingolipid-associated transcriptional heterogeneity but do not directly measure sphingolipid metabolic activity. CCDC80 should also not be interpreted as a canonical sphingolipid metabolic gene; rather, it was identified as a fibroblast-associated hub gene linked to sphingolipid-defined molecular heterogeneity. Targeted sphingolipidomics and metabolomic analyses are required to clarify this relationship. Fourth, direct integration of NMF-derived bulk subtypes with single-cell fibroblast subsets was not feasible because matched bulk and single-cell samples were unavailable. Finally, although in vitro experiments supported a role of CCDC80 in fibroblast activation, its downstream signaling mechanisms and in vivo function were not directly validated. Future studies should include matched multi-omics cohorts, pathway-specific experiments, rescue assays, and pulmonary fibrosis animal models.

## Conclusion

In conclusion, this study established sphingolipid-associated molecular stratification and developed diagnostic and prognostic signatures for IPF. CCDC80 was identified as a fibroblast-associated hub gene linked to sphingolipid-defined molecular heterogeneity and may contribute to fibrotic remodeling. Further lipidomic and mechanistic studies are required to clarify whether CCDC80 is directly or indirectly involved in sphingolipid metabolism.

## Supplementary Information

Below is the link to the electronic supplementary material.

Supplementary Material 1

Supplementary Material 2

Supplementary Material 3

Supplementary Material 4

Supplementary Material 5

Supplementary Material 6

Supplementary Material 7

Supplementary Material 8

Supplementary Material 9

## Data Availability

No datasets were generated or analysed during the current study.
