# Supplementary material for: Integrative Multi-omics and machine learning analysis of sphingolipid-associated molecular stratification identifies diagnostic and prognostic signatures in idiopathic pulmonary fibrosis
Source: Orphanet J Rare Dis. 2026 Aug 25;21:384. doi: 10.1186/s13023-026-04563-4 (PMC13733085; doi:10.1186/s13023-026-04563-4)
Supplement: Supplementary file 1 [file 13023_2026_4563_MOESM1_ESM.pdf]

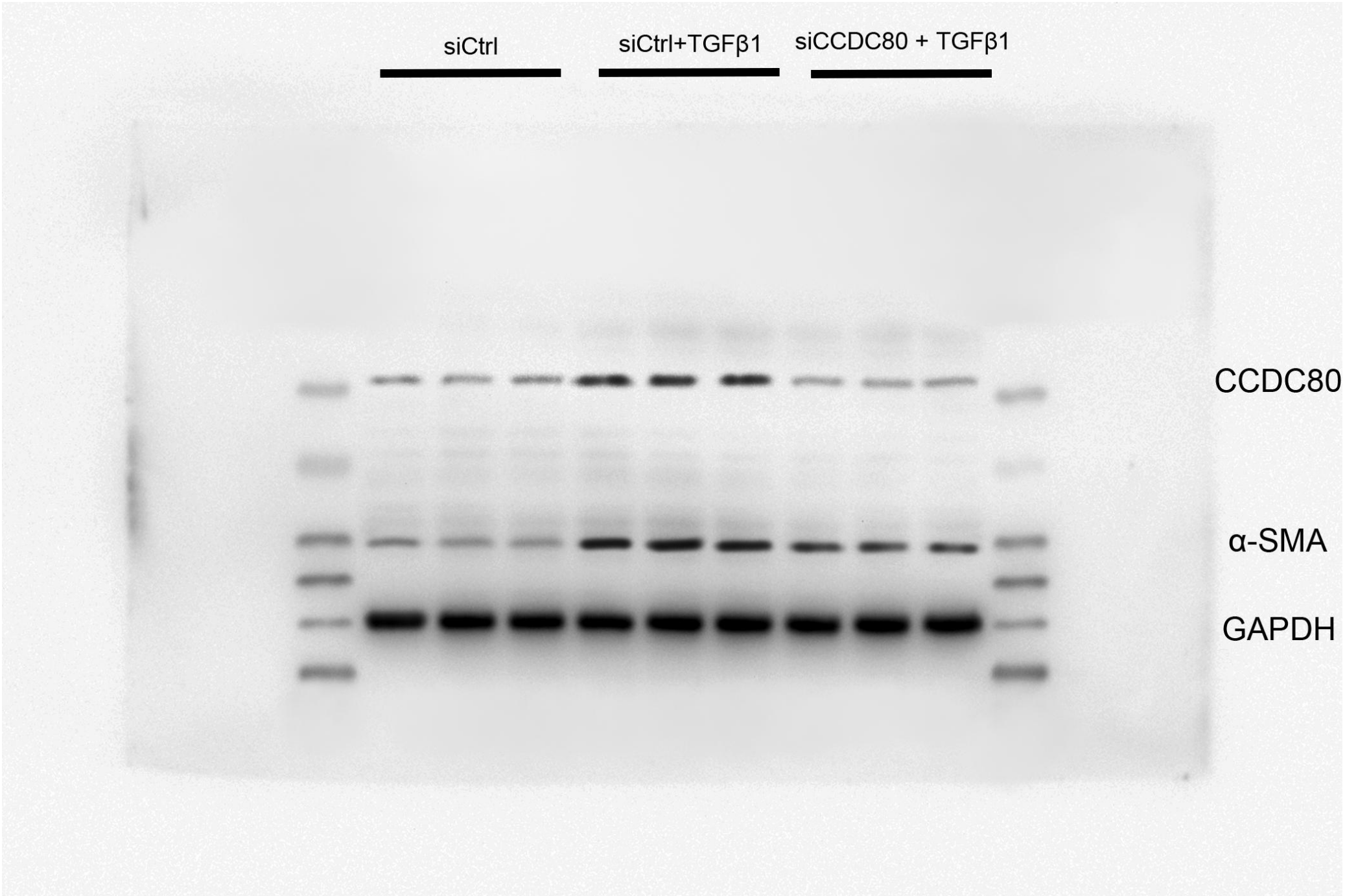
siCtrl
siCtrl+TGFβ1
siCCDC80 + TGFβ1
CCDC80
α-SMA
GAPDH
