# Supplementary material for: Integrative Multi-omics and machine learning analysis of sphingolipid-associated molecular stratification identifies diagnostic and prognostic signatures in idiopathic pulmonary fibrosis
Source: Orphanet J Rare Dis. 2026 Aug 25;21:384. doi: 10.1186/s13023-026-04563-4 (PMC13733085; doi:10.1186/s13023-026-04563-4)
Supplement: Supplementary file 2 [file 13023_2026_4563_MOESM2_ESM.pdf]

R Code for Diagnostic and Prognostic Machine Learning

Workflow Overview

Workflow	Model combinations	Training and validation strategy	Main outputs
Diagnostic workflow	113 algorithm combinations	Training: GSE47460; Validation: GSE32537, GSE53845, GSE150910	AUC, predicted probability, predicted class, selected features
Prognostic workflow	101 algorithm combinations	Training: GSE28042; Validation: GSE27957, GSE93606	C-index, risk score, selected features

Part I. Diagnostic Machine Learning Main Script

```
# Code summary: algorithm combinations for variable selection and predictive model construction.
#
# Algorithms include LASSO, Ridge, elastic net, Stepglm/StepCox, SVM, boosting, PLS, random forest, GBM/XGBoost, Naive Bayes, and related survival learners.
#
# Main workflow:
# A two-stage machine-learning framework is used: one algorithm performs feature selection and another builds the prediction model.
# Model combinations are evaluated in external cohorts by AUC, and the results are visualized using heatmaps.
#
# Required input files:
# Training expression matrix: rows are features and columns are samples. Feature identifiers should be consistent with the validation matrix.
# Training class information: rows are samples and at least one binary outcome column coded as 0/1 is required.
# Validation expression matrix: rows are features and columns are samples. Feature identifiers should match the training matrix.
# Validation label/survival information: rows are samples and a cohort indicator column is required for cohort-specific evaluation.
# Note: prefiltering the expression matrix to a focused candidate feature set is recommended to reduce computation time.
#
# Use case:
# This script is suitable when many candidate variables require simultaneous feature selection, model selection, and classifier construction.
#
# Main outputs:
# The outputs include models from 113 algorithm combinations, AUC matrices, prediction scores, predicted classes, selected features, and heatmaps.

# Set project directory.
work.path <- file.path("<PROJECT_DIR>", "PredictiveML_3.0")
setwd(work.path)

# Set subdirectories.
code.path <- file.path(work.path, "Codes")
data.path <- file.path(work.path, "InputData")
res.path <- file.path(work.path, "Results")
fig.path <- file.path(work.path, "Figures")

# Create directories if they do not exist.
if (!dir.exists(data.path)) dir.create(data.path)
if (!dir.exists(res.path)) dir.create(res.path)
if (!dir.exists(fig.path)) dir.create(fig.path)
if (!dir.exists(code.path)) dir.create(code.path)

# install.packages("randomForestSRC")
# install.packages("snowfall")
# install.packages("xgboost")

# Load required R packages.
library(openxlsx)
library(seqinr)
library(plyr)
library(randomForestSRC)
library(glmnet)
library(plsRglm)
library(gbm)
library(caret)
library(mboost)
library(e1071)
library(BART)
library(MASS)
library(snowfall)
library(xgboost)
library(ComplexHeatmap)
library(RColorBrewer)
library(pROC)

# Load the model-training and model-evaluation functions.
source(file.path(code.path, "ML.R"))
# Choose the final model type: panML keeps algorithm-specific models; multiLogistic/multiCox refits selected variables using multivariable logistic/Cox regression.
FinalModel <- c("panML", "multiLogistic")[2]

## Training Cohort ----------------------------------------------------------
# Training expression matrix: rows are features and columns are samples. Feature identifiers should be consistent with the validation matrix.
Train_expr <- read.table(file.path(data.path, "Training_expr.txt"), header = T, sep = "\t", row.names = 1,check.names = F,stringsAsFactors = F)
```

```
# Rows are samples; at least one binary outcome column coded as 0/1 is required.
Train_class <- read.table(file.path(data.path, "Training_class.txt"), header = T, sep = "\t", row.names = 1,check.names = F,stringsAsFactors = F)
# Keep samples shared by the training expression matrix and labels.
comsam <- intersect(rownames(Train_class), colnames(Train_expr))
Train_expr <- Train_expr[,comsam]; Train_class <- Train_class[comsam,,drop = F]

## Validation Cohort -------------------------------------------------------
# Validation expression matrix: rows are features and columns are samples. Feature identifiers should match the training matrix.
Test_expr <- read.table(file.path(data.path, "Testing_expr.txt"), header = T, sep = "\t", row.names = 1,check.names = F,stringsAsFactors = F)
# Rows are samples; at least one binary outcome column coded as 0/1 is required.
Test_class <- read.table(file.path(data.path, "Testing_class.txt"), header = T, sep = "\t", row.names = 1,check.names = F,stringsAsFactors = F)
# Keep samples shared by the validation expression matrix and labels.
comsam <- intersect(rownames(Test_class), colnames(Test_expr))
Test_expr <- Test_expr[,comsam]; Test_class <- Test_class[comsam,,drop = F]

# Keep shared genes/features between training and validation matrices.
comgene <- intersect(rownames(Train_expr),rownames(Test_expr))
Train_expr <- t(Train_expr[comgene,]) # Convert the expression matrix to samples as rows and genes/features as columns for modeling.
Test_expr <- t(Test_expr[comgene,]) # Convert the expression matrix to samples as rows and genes/features as columns for modeling.

# Perform cohort-specific scaling if required.
## data: expression data with samples as rows and genes/features as columns.
## cohort: sample cohort indicator; if omitted, all samples are treated as one cohort.
## centerFlags/scaleFlags: options controlling mean-centering and variance scaling.
## NULL means no centering or scaling by default.
## A single TRUE/FALSE value applies to all cohorts.
## A TRUE/FALSE vector can be supplied to specify cohort-wise scaling settings.
## Example: centerFlags = c(FALSE, FALSE, FALSE, TRUE, TRUE) scales only the fourth and fifth cohorts.
## Named vectors are matched by cohort name rather than by order.
Train_set = scaleData(data = Train_expr, centerFlags = F, scaleFlags = F)
names(x = split(as.data.frame(Test_expr), f = Test_class$Cohort)) # Ensure that validation cohort scaling follows this cohort order.
Test_set = scaleData(data = Test_expr, cohort = Test_class$Cohort, centerFlags = F, scaleFlags = F)
# summary(apply(Train_set, 2, var))
# summary(apply(Test_set, 2, var))
# Check the scaling results.

# Model training and validation -------------------------------------------

## method list --------------------------------------------------------
# The method list records algorithm combinations in the format feature-selection method plus modeling method.
# Only Stepglm and Enet accept method parameters in this diagnostic script.
methods <- read.xlsx(file.path(code.path, "methods.xlsx"), startRow = 2)
methods <- methods$Model
methods <- gsub("-| ", "", methods)

## Train the model --------------------------------------------------------

classVar = "outcome" # Set the binary outcome variable name.
min.selected.var = 5 # Set the minimum number of predictors required for a valid model.

## Pre-training: aggregate feature-selection steps across all methods to avoid redundant computation.
Variable = colnames(Train_set)
preTrain.method =  strsplit(methods, "\\+") # Identify whether each method requires a pre-training feature-selection step.
preTrain.method = lapply(preTrain.method, function(x) rev(x)[-1]) # Remove the final modeling algorithm and retain only feature-selection algorithms.
preTrain.method = unique(unlist(preTrain.method)) # Collect unique feature-selection algorithms to avoid repeated computation.

preTrain.var <- list() # Store variables retained by each feature-selection algorithm.
set.seed(seed = 777) # Set the random seed to ensure reproducibility.
for (method in preTrain.method){
  preTrain.var[[method]] = RunML(method = method, # Machine-learning method used for feature selection.
                                 Train_set = Train_set, # Candidate predictors in the training cohort.
                                 Train_label = Train_class, # Training labels.
                                 mode = "Variable", # Running mode: Variable for feature selection and Model for model fitting.
                                 classVar = classVar) # Training class variable; it must be present in Train_class.
}
preTrain.var[["simple"]] <- colnames(Train_set) # Record the unfiltered feature set as the simple baseline feature-selection option.

## Model training
model <- list() # Initialize the model list.
set.seed(seed = 777) # Set the random seed to ensure reproducibility.
```

```r
Train_set_bk = Train_set # Back up the training matrix because one function requires object names to be preserved.
for (method in methods){
  cat(match(method, methods), ":", method, "\n")
  method_name = method # Name of the current algorithm combination.
  method <- strsplit(method, "\\+")[[1]] # Split the combination into feature-selection and modeling algorithms.

  if (length(method) == 1) method <- c("simple", method) # If no feature-selection method is specified, use the simple unfiltered feature set.

  Variable = preTrain.var[[method[1]]] # Retrieve variables selected by the first-stage method.
  Train_set = Train_set_bk[, Variable] # Subset the training matrix to the selected variables.
  Train_label = Train_class # Use these object names to avoid function-specific object lookup errors.
  model[[method_name]] <- RunML(method = method[2], # Fit the final model using the second-stage algorithm.
                                Train_set = Train_set, # Candidate predictors in the training cohort.
                                Train_label = Train_label, # Training labels.
                                mode = "Model", # Running mode: Variable for feature selection and Model for model fitting.
                                classVar = classVar) # Training class variable; it must be present in Train_class.

# Discard models retaining fewer predictors than the predefined threshold.
  if(length(ExtractVar(model[[method_name]])) <= min.selected.var) {
    model[[method_name]] <- NULL
  }
}
Train_set = Train_set_bk; rm(Train_set_bk) # Restore the original training matrix and remove the backup object.
saveRDS(model, file.path(res.path, "model.rds")) # Save all fitted model objects and intermediate outputs.

if (FinalModel == "multiLogistic"){
  logisticmodel <- lapply(model, function(fit){ # Refit a multivariable logistic model using the variables selected by each algorithm to calculate sample-level classification probabilities.
    tmp <- glm(formula = Train_class[[classVar]] ~ .,
               family = "binomial",
               data = as.data.frame(Train_set[, ExtractVar(fit)]))
    tmp$subFeature <- ExtractVar(fit) # Store predictors used in the final logistic model.
    return(tmp)
  })
}
saveRDS(logisticmodel, file.path(res.path, "logisticmodel.rds")) # Save the final multivariable logistic models.

## Evaluate the model ----------------------------------------------------

# Load saved model objects.
model <- readRDS(file.path(res.path, "model.rds"))
# Save the final multivariable logistic models.
methodsValid <- names(model)

# Calculate sample-level prediction scores from expression data.
# Predicted probability.
RS_list <- list()
for (method in methodsValid){
  RS_list[[method]] <- CalPredictScore(fit = model[[method]],
                                       new_data = rbind.data.frame(Train_set,Test_set)) # Version update.
}
RS_mat <- as.data.frame(t(do.call(rbind, RS_list)))
write.table(RS_mat, file.path(res.path, "RS_mat.txt"),sep = "\t", row.names = T, col.names = NA, quote = F) # Write the prediction/risk-score matrix.

# Predict binary class labels from expression data.
Class_list <- list()
for (method in methodsValid){
  Class_list[[method]] <- PredictClass(fit = model[[method]],
                                       new_data = rbind.data.frame(Train_set,Test_set)) # Version update.
}
Class_mat <- as.data.frame(t(do.call(rbind, Class_list)))
# Optional: merge predicted classes with validation sample information.
write.table(Class_mat, file.path(res.path, "Class_mat.txt"), # Write binary class predictions from each algorithm.
            sep = "\t", row.names = T, col.names = NA, quote = F)

# Extract selected features as a list.
fea_list <- list()
for (method in methodsValid) {
  fea_list[[method]] <- ExtractVar(model[[method]])
}
```

```r
# Extract selected features as a data frame.
fea_df <- lapply(model, function(fit){
  data.frame(ExtractVar(fit))
})
fea_df <- do.call(rbind, fea_df)
fea_df$algorithm <- gsub("(.+)\\.(.+$)", "\\1", rownames(fea_df))
colnames(fea_df)[1] <- "features"
write.table(fea_df, file.path(res.path, "fea_df.txt"), # The data frame contains algorithm names and selected features.
            sep = "\t", row.names = F, col.names = T, quote = F)

# Evaluate model performance by C-index or cohort-specific metrics.
AUC_list <- list()
for (method in methodsValid){
  AUC_list[[method]] <- RunEval(fit = model[[method]], # Diagnostic classification model.
                                Test_set = Test_set, # Validation predictors; all features used by the model must be available.
                                Test_label = Test_class, # Training class labels; all required variables must be available.
                                Train_set = Train_set, # Training expression matrix: rows are features and columns are samples. Feature identifiers should be consistent with the validation matrix.
                                Train_label = Train_class, # Training class labels; all required variables must be available.
                                Train_name = "GSE47460", # Provide a training cohort label if the training cohort should also be evaluated; otherwise it is labeled as Training.
                                cohortVar = "Cohort", # Important: specify the cohort indicator variable; this column must exist.
                                classVar = classVar) # Binary outcome variable used for evaluation.
}
AUC_mat <- do.call(rbind, AUC_list)
write.table(AUC_mat, file.path(res.path, "AUC_mat.txt"),
            sep = "\t", row.names = T, col.names = T, quote = F)

# Plot ----------------------------------------------------------------------
## =========================================================
## Load required R packages.
## =========================================================


library(ComplexHeatmap)
library(circlize)
library(RColorBrewer)
library(grid)

## =========================================================
## Read and prepare the performance matrix.
## =========================================================
AUC_mat <- read.table(file.path(res.path, "AUC_mat.txt"),
                      sep = "\t", row.names = 1, header = TRUE,
                      check.names = FALSE, stringsAsFactors = FALSE)

avg_AUC <- apply(AUC_mat, 1, mean, na.rm = TRUE)
avg_AUC <- sort(avg_AUC, decreasing = TRUE)
AUC_mat <- AUC_mat[names(avg_AUC), ]

AUC_mat_num <- as.matrix(AUC_mat)
text_mat <- matrix(sprintf("%.3f", AUC_mat_num),
                   nrow = nrow(AUC_mat_num),
                   dimnames = dimnames(AUC_mat_num))

## =========================================================
## Publication-style color palette.
## =========================================================
col_fun <- colorRamp2(
  c(0.50, 0.60, 0.70, 0.80),
  c("#f7f4e9", "#cfe6de", "#6ba48f", "#d94f45")
)

if (ncol(AUC_mat) < 3) {
  CohortCol <- c("#4c8dae", "#c19a3f")[1:ncol(AUC_mat)]
} else {
  CohortCol <- brewer.pal(n = max(3, ncol(AUC_mat)), name = "Paired")[1:ncol(AUC_mat)]
}
names(CohortCol) <- colnames(AUC_mat)

top_ha <- HeatmapAnnotation(
  cohort = colnames(AUC_mat),
```

```r
  col = list(cohort = CohortCol),
  annotation_name_side = "left",
  annotation_legend_param = list(title = "Cohort")
)

## ============================================================
## Left AUC panel with fixed-width bar and white values.
## Remove column_title to avoid alignment issues.
## ============================================================
AUC_left_mat <- matrix(1,
                       nrow = length(avg_AUC),
                       ncol = 1,
                       dimnames = list(names(avg_AUC), "AUC"))

ht_left <- Heatmap(
  AUC_left_mat,
  name = "AUC_left",
  col = "#6f7a4b",
  show_heatmap_legend = FALSE,
  cluster_rows = FALSE,
  cluster_columns = FALSE,
  width = unit(22, "mm"),
  show_row_names = FALSE,
  column_title = NULL, # Remove the title to avoid layout misalignment.
  cell_fun = function(j, i, x, y, width, height, fill) {
    grid.text(
      sprintf("%.3f", avg_AUC[i]),
      x = x, y = y,
      gp = gpar(col = "white",
                fontsize = 17,
                fontface = "bold")
    )
  }
)

## ============================================================
## Main heatmap showing cohort-specific values.
## ============================================================
ht_right <- Heatmap(
  AUC_mat_num,
  name = "AUC",
  col = col_fun,
  cluster_rows = FALSE,
  cluster_columns = FALSE,
  rect_gp = gpar(col = "white", lwd = 0.5),

  row_names_side = "right",
  row_names_gp   = gpar(fontsize = 17),

## Place column names at the top, horizontally and centered.
  column_names_side     = "top",
  column_names_gp       = gpar(fontsize = 13),
  column_names_rot      = 0, # Horizontal labels.
  column_names_centered = TRUE, # Center-align labels.

  top_annotation = top_ha,
  heatmap_legend_param = list(
    title = "AUC",
    at = seq(0.50, 0.80, 0.05),
    labels = sprintf("%.2f", seq(0.50, 0.80, 0.05)),
    legend_height = unit(48, "mm")
  ),
  cell_fun = function(j, i, x, y, width, height, fill) {
    grid.text(
      text_mat[i, j],
      x = x, y = y,
      gp = gpar(fontsize = 17)
    )
  }
)
```

```
## ========================================================
## Export the PDF figure.
## ========================================================
cellwidth  <- 0.68
cellheight <- 0.38

pdf_width  <- cellwidth * ncol(AUC_mat) + 9
pdf_height <- cellheight * nrow(AUC_mat) + 4

pdf(file.path(fig.path, "AUC_CNstyle_AUCleft_bigFont.pdf"),
    width  = pdf_width,
    height = pdf_height,
    useDingbats = FALSE)

draw(ht_left + ht_right,
     heatmap_legend_side = "left",
     annotation_legend_side = "left",
     padding = unit(c(6, 6, 6, 6), "mm"))

dev.off()
```

Part II. Diagnostic Machine Learning Functions

```
# all ML algorithms
RunML <- function(method, Train_set, Train_label, mode = "Model", classVar){
  # for example: Enet [alpha=0.4]
  method = gsub(" ", "", method) # Remove spaces from method parameters, e.g., Enet [alpha=0.4].
  method_name = gsub("(\\w+)\\[(.+)\\]", "\\1", method)  # get name of ML algorithm, e.g., Enet
  method_param = gsub("(\\w+)\\[(.+)\\]", "\\2", method) # get parameter of ML algorithm, e.g., alpha=0.4

  method_param = switch(
    EXPR = method_name,
    "Enet" = list("alpha" = as.numeric(gsub("alpha=", "", method_param))),
    "Stepglm" = list("direction" = method_param),
    NULL
  )
  message("Run ", method_name, " algorithm for ", mode, "; ",
          method_param, ";",
          " using ", ncol(Train_set), " Variables")

  args = list("Train_set" = Train_set,
              "Train_label" = Train_label,
              "mode" = mode,
              "classVar" = classVar)
  args = c(args, method_param)

  obj <- do.call(what = paste0("Run", method_name),
                 args = args)

  if(mode == "Variable"){
    message(length(obj), " Variables retained;\n")
  }else{message("\n")}
  return(obj)
}

RunEnet <- function(Train_set, Train_label, mode, classVar, alpha){
  cv.fit = cv.glmnet(x = Train_set,
                     y = Train_label[[classVar]],
                     family = "binomial", alpha = alpha, nfolds = 10)
  fit = glmnet(x = Train_set,
               y = Train_label[[classVar]],
               family = "binomial", alpha = alpha, lambda = cv.fit$lambda.min)
  fit$subFeature = colnames(Train_set)
  if (mode == "Model") return(fit)
  if (mode == "Variable") return(ExtractVar(fit))
}

RunLasso <- function(Train_set, Train_label, mode, classVar){
  RunEnet(Train_set, Train_label, mode, classVar, alpha = 1)
}

RunRidge <- function(Train_set, Train_label, mode, classVar){
  RunEnet(Train_set, Train_label, mode, classVar, alpha = 0)
}

RunStepglm <- function(Train_set, Train_label, mode, classVar, direction){
  fit <- step(glm(formula = Train_label[[classVar]] ~ .,
                  family = "binomial",
                  data = as.data.frame(Train_set)),
              direction = direction, trace = 0)
  fit$subFeature = colnames(Train_set)
  if (mode == "Model") return(fit)
  if (mode == "Variable") return(ExtractVar(fit))
}

RunSVM <- function(Train_set, Train_label, mode, classVar){
  data <- as.data.frame(Train_set)
  data[[classVar]] <- as.factor(Train_label[[classVar]])
  fit = svm(formula = eval(parse(text = paste(classVar, "~."))),
            data= data, probability = T)
  fit$subFeature = colnames(Train_set)
```

```
  if (mode == "Model") return(fit)
  if (mode == "Variable") return(ExtractVar(fit))
}

RunLDA <- function(Train_set, Train_label, mode, classVar){
  data <- as.data.frame(Train_set)
  data[[classVar]] <- as.factor(Train_label[[classVar]])
  fit = train(eval(parse(text = paste(classVar, "~."))),
              data = data,
              method="lda",
              trControl = trainControl(method = "cv"))
  fit$subFeature = colnames(Train_set)
  if (mode == "Model") return(fit)
  if (mode == "Variable") return(ExtractVar(fit))
}

RunglmBoost <- function(Train_set, Train_label, mode, classVar){
  data <- cbind(Train_set, Train_label[classVar])
  data[[classVar]] <- as.factor(data[[classVar]])
  fit <- glmboost(eval(parse(text = paste(classVar, "~."))),
                  data = data,
                  family = Binomial())

  cvm <- cvrisk(fit, papply = lapply,
                folds = cv(model.weights(fit), type = "kfold"))
  fit <- glmboost(eval(parse(text = paste(classVar, "~."))),
                  data = data,
                  family = Binomial(),
                  control = boost_control(mstop = max(mstop(cvm), 40)))

  fit$subFeature = colnames(Train_set)
  if (mode == "Model") return(fit)
  if (mode == "Variable") return(ExtractVar(fit))
}

RunplsRglm <- function(Train_set, Train_label, mode, classVar){
  cv.plsRglm.res = cv.plsRglm(formula = Train_label[[classVar]] ~ .,
                              data = as.data.frame(Train_set),
                              nt=10, verbose = FALSE)
  fit <- plsRglm(Train_label[[classVar]],
               as.data.frame(Train_set),
               modele = "pls-glm-logistic",
               verbose = F, sparse = T)
  fit$subFeature = colnames(Train_set)
  if (mode == "Model") return(fit)
  if (mode == "Variable") return(ExtractVar(fit))
}

RunRF <- function(Train_set, Train_label, mode, classVar){
  rf_nodesize = 5 # may modify
  Train_label[[classVar]] <- as.factor(Train_label[[classVar]])
  fit <- rfsrc(formula = formula(paste0(classVar, "~.")),
               data = cbind(Train_set, Train_label[classVar]),
               ntree = 1000, nodesize = rf_nodesize,
               importance = T,
               proximity = T,
               forest = T)
  fit$subFeature = colnames(Train_set)
  if (mode == "Model") return(fit)
  if (mode == "Variable") return(ExtractVar(fit))
}

RunGBM <- function(Train_set, Train_label, mode, classVar){
  fit <- gbm(formula = Train_label[[classVar]] ~ .,
             data = as.data.frame(Train_set),
             distribution = 'bernoulli',
             n.trees = 10000,
             interaction.depth = 3,
             n.minobsinnode = 10,
             shrinkage = 0.001,
```

```r
             cv.folds = 10,n.cores = 6)
  best <- which.min(fit$cv.error)
  fit <- gbm(formula = Train_label[[classVar]] ~ .,
             data = as.data.frame(Train_set),
             distribution = 'bernoulli',
             n.trees = best,
             interaction.depth = 3,
             n.minobsinnode = 10,
             shrinkage = 0.001, n.cores = 8)
  fit$subFeature = colnames(Train_set)
  if (mode == "Model") return(fit)
  if (mode == "Variable") return(ExtractVar(fit))
}

RunXGBoost <- function(Train_set, Train_label, mode, classVar){
  indexes = createFolds(Train_label[[classVar]], k = 5, list=T)
  CV <- unlist(lapply(indexes, function(pt){
    dtrain = xgb.DMatrix(data = Train_set[-pt, ],
                         label = Train_label[-pt, ])
    dtest = xgb.DMatrix(data = Train_set[pt, ],
                        label = Train_label[pt, ])
    watchlist <- list(train=dtrain, test=dtest)

    bst <- xgb.train(data=dtrain,
                     max.depth=2, eta=1, nthread = 2, nrounds=10,
                     watchlist=watchlist,
                     objective = "binary:logistic", verbose = F)
    which.min(bst$evaluation_log$test_logloss)
  }))

  nround <- as.numeric(names(which.max(table(CV))))
  fit <- xgboost(data = Train_set,
                 label = Train_label[[classVar]],
                 max.depth = 2, eta = 1, nthread = 2, nrounds = nround,
                 objective = "binary:logistic", verbose = F)
  fit$subFeature = colnames(Train_set)

  if (mode == "Model") return(fit)
  if (mode == "Variable") return(ExtractVar(fit))
}

RunNaiveBayes <- function(Train_set, Train_label, mode, classVar){
  data <- cbind(Train_set, Train_label[classVar])
  data[[classVar]] <- as.factor(data[[classVar]])
  fit <- naiveBayes(eval(parse(text = paste(classVar, "~."))),
                    data = data)
  fit$subFeature = colnames(Train_set)
  if (mode == "Model") return(fit)
  if (mode == "Variable") return(ExtractVar(fit))
}

# DRF is not suitable for binary classification here and is therefore omitted.
# RunDRF <- function(Train_set, Train_label, mode, classVar){
#   Train_label <- data.frame(
#     "0" = as.numeric(Train_label == 0),
#     "1" = as.numeric(Train_label == 1)
#   )
#   fit <- drf(X = Train_set,
#              Y = Train_label,
#              compute.variable.importance = F)
#   fit$subFeature = colnames(Train_set)
#
#   summary(predict(fit, functional = "mean", as.matrix(Train_set))$mean)
#
#   if (mode == "Model") return(fit)
#   if (mode == "Variable") return(ExtractVar(fit))
# }

quiet <- function(..., messages=FALSE, cat=FALSE){
  if(!cat){
```

```r
    sink(tempfile())
    on.exit(sink())
  }
  out <- if(messages) eval(...) else suppressMessages(eval(...))
  out
}

standarize.fun <- function(indata, centerFlag, scaleFlag) {
  scale(indata, center=centerFlag, scale=scaleFlag)
}

# Version update.
scaleData <- function(data, cohort = NULL, centerFlags = NULL, scaleFlags = NULL){
  samplename = rownames(data)
  if (is.null(cohort)){
    data <- list(data); names(data) = "training"
  }else{
    data <- split(as.data.frame(data), cohort)
  }

  if (is.null(centerFlags)){
    centerFlags = F; message("No centerFlags found, set as FALSE")
  }
  if (length(centerFlags)==1){
    centerFlags = rep(centerFlags, length(data)); message("set centerFlags for all cohort as ", unique(centerFlags))
  }
  if (is.null(names(centerFlags))){
    names(centerFlags) <- names(data); message("match centerFlags with cohort by order\n")
  }

  if (is.null(scaleFlags)){
    scaleFlags = F; message("No scaleFlags found, set as FALSE")
  }
  if (length(scaleFlags)==1){
    scaleFlags = rep(scaleFlags, length(data)); message("set scaleFlags for all cohort as ", unique(scaleFlags))
  }
  if (is.null(names(scaleFlags))){
    names(scaleFlags) <- names(data); message("match scaleFlags with cohort by order\n")
  }

  centerFlags <- centerFlags[names(data)]; scaleFlags <- scaleFlags[names(data)]
  outdata <- mapply(standarize.fun, indata = data, centerFlag = centerFlags, scaleFlag = scaleFlags, SIMPLIFY = F)
  # lapply(out.data, function(x) summary(apply(x, 2, var)))
  outdata <- do.call(rbind, outdata)
  outdata <- outdata[samplename, ]
  return(outdata)
}

ExtractVar <- function(fit){
  Feature <- quiet(switch(
    EXPR = class(fit)[1],
    "lognet" = rownames(coef(fit))[which(coef(fit)[, 1]!=0)], # This model does not directly perform feature selection; features with zero coefficients are removed where applicable.
    "glm" = names(coef(fit)), # Stepwise regression can perform variable selection.
    "svm.formula" = fit$subFeature, # SVM does not perform feature selection, so all input features are retained by default.
    "train" = fit$coefnames, # LDA does not perform feature selection, so all input features are retained by default.
    "glmboost" = names(coef(fit)[abs(coef(fit))>0]), # glmBoost does not directly perform feature selection; variables with nonzero coefficients are retained.
    "plsRglmmodel" = rownames(fit$Coeffs)[fit$Coeffs!=0], # plsRglm does not directly perform feature selection; variables with nonzero coefficients are retained.
    "rfsrc" = var.select(fit, verbose = F)$topvars, # rfsrc can perform variable selection.
    "gbm" = rownames(summary.gbm(fit, plotit = F))[summary.gbm(fit, plotit = F)$rel.inf>0], # GBM performs feature selection by retaining variables with nonzero relative influence.
    "xgb.Booster" = fit$subFeature, # XGBoost does not perform feature selection, so all input features are retained by default.
    "naiveBayes" = fit$subFeature # Naive Bayes does not perform feature selection, so all input features are retained by default.
# The DRF coefficient extraction function returns NA, so all variables would be retained by default.
  ))

  Feature <- setdiff(Feature, c("(Intercept)", "Intercept"))
  return(Feature)
}


CalPredictScore <- function(fit, new_data, type = "lp"){
```

```
  new_data <- new_data[, fit$subFeature]
  RS <- quiet(switch(
    EXPR = class(fit)[1],
    "lognet"      = predict(fit, type = 'response', as.matrix(new_data)), # response
    "glm"         = predict(fit, type = 'response', as.data.frame(new_data)), # response
    "svm.formula" = predict(fit, as.data.frame(new_data), probability = T), #
    "train"       = predict(fit, new_data, type = "prob")[[2]],
    "glmboost"    = predict(fit, type = "response", as.data.frame(new_data)), # response
    "plsRglmmodel" = predict(fit, type = "response", as.data.frame(new_data)), # response
    "rfsrc"        = predict(fit, as.data.frame(new_data))$predicted[, "1"],
    "gbm"          = predict(fit, type = 'response', as.data.frame(new_data)), # response
    "xgb.Booster" = predict(fit, as.matrix(new_data)),
    "naiveBayes" = predict(object = fit, type = "raw", newdata = new_data)[, "1"]
    # "drf" = predict(fit, functional = "mean", as.matrix(new_data))$mean
  ))
  RS = as.numeric(as.vector(RS))
  names(RS) = rownames(new_data)
  return(RS)
}

PredictClass <- function(fit, new_data){
  new_data <- new_data[, fit$subFeature]
  label <- quiet(switch(
    EXPR = class(fit)[1],
    "lognet"      = predict(fit, type = 'class', as.matrix(new_data)),
    "glm"         = ifelse(test = predict(fit, type = 'response', as.data.frame(new_data))>0.5,
                           yes = "1", no = "0"), # glm does not return class labels; probabilities greater than 0.5 are assigned to class 1.
    "svm.formula" = predict(fit, as.data.frame(new_data), decision.values = T), #
    "train"       = predict(fit, new_data, type = "raw"),
    "glmboost"    = predict(fit, type = "class", as.data.frame(new_data)),
    "plsRglmmodel" = ifelse(test = predict(fit, type = 'response', as.data.frame(new_data))>0.5,
                            yes = "1", no = "0"), # plsRglm cannot use factor outcomes directly; class labels are assigned using a probability threshold of 0.5.
    "rfsrc"        = predict(fit, as.data.frame(new_data))$class,
    "gbm"          = ifelse(test = predict(fit, type = 'response', as.data.frame(new_data))>0.5,
                            yes = "1", no = "0"), # GBM does not directly return class labels; probabilities greater than 0.5 are assigned to class 1.
    "xgb.Booster" = ifelse(test = predict(fit, as.matrix(new_data))>0.5,
                           yes = "1", no = "0"), # XGBoost does not directly return class labels; probabilities greater than 0.5 are assigned to class 1.
    "naiveBayes" = predict(object = fit, type = "class", newdata = new_data)
    # "drf" = predict(fit, functional = "mean", as.matrix(new_data))$mean
  ))
  label = as.character(as.vector(label))
  names(label) = rownames(new_data)
  return(label)
}

RunEval <- function(fit,
                    Test_set = NULL,
                    Test_label = NULL,
                    Train_set = NULL,
                    Train_label = NULL,
                    Train_name = NULL,
                    cohortVar = "Cohort",
                    classVar){

  if(!is.element(cohortVar, colnames(Test_label))) {
    stop(paste0("There is no [", cohortVar, "] indicator, please fill in one more column!"))
  }

  if((!is.null(Train_set)) & (!is.null(Train_label))) {
    new_data <- rbind.data.frame(Train_set[, fit$subFeature],
                                 Test_set[, fit$subFeature])

    if(!is.null(Train_name)) {
      Train_label$Cohort <- Train_name
    } else {
      Train_label$Cohort <- "Training"
    }
    colnames(Train_label)[ncol(Train_label)] <- cohortVar
    Test_label <- rbind.data.frame(Train_label[,c(cohortVar, classVar)],
                                  Test_label[,c(cohortVar, classVar)])
```

```
    Test_label[,1] <- factor(Test_label[,1],
                            levels = c(unique(Train_label[,cohortVar]), setdiff(unique(Test_label[,cohortVar]),unique(Train_label[,cohortVar]))))
  } else {
    new_data <- Test_set[, fit$subFeature]
  }

  RS <- suppressWarnings(CalPredictScore(fit = fit, new_data = new_data))

  Predict.out <- Test_label
  Predict.out$RS <- as.vector(RS)
  Predict.out <- split(x = Predict.out, f = Predict.out[,cohortVar])
  unlist(lapply(Predict.out, function(data){
    as.numeric(auc(suppressMessages(roc(data[[classVar]], data$RS))))
  }))
}

SimpleHeatmap <- function(Cindex_mat, avg_Cindex,
                          CohortCol, barCol,
                          cellwidth = 1, cellheight = 0.5,
                          cluster_columns, cluster_rows){
  col_ha = columnAnnotation("Cohort" = colnames(Cindex_mat),
                            col = list("Cohort" = CohortCol),
                            show_annotation_name = F)

  row_ha = rowAnnotation(bar = anno_barplot(avg_Cindex, bar_width = 0.8, border = FALSE,
                                            gp = gpar(fill = barCol, col = NA),
                                            add_numbers = T, numbers_offset = unit(-10, "mm"),
                                            axis_param = list("labels_rot" = 0),
                                            numbers_gp = gpar(fontsize = 9, col = "white"),
                                            width = unit(3, "cm")),
                         show_annotation_name = F)

  Heatmap(as.matrix(Cindex_mat), name = "AUC",
          right_annotation = row_ha,
          top_annotation = col_ha,
# Yellow-green palette.
          col = c("#4195C1", "#FFFFFF", "#CB5746"), # Blue-red palette.
          rect_gp = gpar(col = "black", lwd = 1), # Use black cell borders.
          cluster_columns = cluster_columns, cluster_rows = cluster_rows, # No clustering is performed because it is not meaningful here.
          show_column_names = FALSE,
          show_row_names = TRUE,
          row_names_side = "left",
          width = unit(cellwidth * ncol(Cindex_mat) + 2, "cm"),
          height = unit(cellheight * nrow(Cindex_mat), "cm"),
          column_split = factor(colnames(Cindex_mat), levels = colnames(Cindex_mat)),
          column_title = NULL,
          cell_fun = function(j, i, x, y, w, h, col) { # add text to each grid
            grid.text(label = format(Cindex_mat[i, j], digits = 3, nsmall = 3),
                      x, y, gp = gpar(fontsize = 10))
          }
  )
}
```

Part III. Prognostic Machine Learning Main Script

```
# Use case:
# This script is suitable when many candidate variables require simultaneous feature selection, model selection, and prognostic model construction.
#
# Main outputs:
# The outputs include models from 101 algorithm combinations, C-index matrices, risk scores, selected features, and heatmaps.

# Set project directory.
work.path <- file.path("<PROJECT_DIR>", "PrognosticML_4.0")
setwd(work.path)

# Set subdirectories.
code.path <- file.path(work.path, "Codes") # Directory for scripts.
data.path <- file.path(work.path, "InputData") # Directory for input data; modify as needed.
res.path <- file.path(work.path, "Results") # Directory for result files.
fig.path <- file.path(work.path, "Figures") # Directory for output figures.

# Create directories if they do not exist.
if (!dir.exists(data.path)) dir.create(data.path)
if (!dir.exists(res.path)) dir.create(res.path)
if (!dir.exists(fig.path)) dir.create(fig.path)
if (!dir.exists(code.path)) dir.create(code.path)

# BiocManager::install("mixOmics")
# BiocManager::install("survcomp")
# devtools::install_github("binderh/CoxBoost")
# install.packages("randomForestSRC")
# install.packages("snowfall")

# Load required R packages.
library(openxlsx)
library(seqinr)
library(plyr)
library(survival)
library(randomForestSRC)
library(glmnet)
library(plsRcox)
library(superpc)
library(gbm)
library(mixOmics)
library(survcomp)
library(CoxBoost)
library(survivalsvm)
library(BART)
library(snowfall)
library(ComplexHeatmap)
library(RColorBrewer)

# Load the model-training and model-evaluation functions.
source(file.path(code.path, "ML.R"))

# Choose the final model type: panML keeps algorithm-specific models; multiLogistic/multiCox refits selected variables using multivariable logistic/Cox regression.
FinalModel <- c("panML", "multiCox")[2]

## Training Cohort ---------------------------------------------------------
# Training expression matrix: rows are features and columns are samples. Feature identifiers should be consistent with the validation matrix.
Train_expr <- read.table(file.path(data.path, "Training_expr.txt"), header = T, sep = "\t", row.names = 1,check.names = F,stringsAsFactors = F)

# Training survival data: rows are samples and outcome columns must include valid survival time and status.
Train_surv <- read.table(file.path(data.path, "Training_surv.txt"), header = T, sep = "\t", row.names = 1,check.names = F,stringsAsFactors = F)
comsam <- intersect(rownames(Train_surv), colnames(Train_expr))
Train_expr <- Train_expr[,comsam]; Train_surv <- Train_surv[comsam,,drop = F]

## Validation Cohort -------------------------------------------------------
# Validation expression matrix: rows are features and columns are samples. Feature identifiers should match the training matrix.
Test_expr <- read.table(file.path(data.path, "Testing_expr.txt"), header = T, sep = "\t", row.names = 1,check.names = F,stringsAsFactors = F)

# Validation survival data: rows are samples and outcome columns must include valid survival time and status.
Test_surv <- read.table(file.path(data.path, "Testing_surv.txt"), header = T, sep = "\t", row.names = 1,check.names = F,stringsAsFactors = F)
```

```r
comsam <- intersect(rownames(Test_surv), colnames(Test_expr))
Test_expr <- Test_expr[,comsam]; Test_surv <- Test_surv[comsam,,drop = F]

# Keep shared genes/features between training and validation matrices.
comgene <- intersect(rownames(Train_expr),rownames(Test_expr))
Train_expr <- t(Train_expr[comgene,]) # Convert the expression matrix to samples as rows and genes/features as columns for modeling.
Test_expr <- t(Test_expr[comgene,]) # Convert the expression matrix to samples as rows and genes/features as columns for modeling.

# Perform cohort-specific scaling if required.
## data: expression data with samples as rows and genes/features as columns.
## cohort: sample cohort indicator; if omitted, all samples are treated as one cohort.
## centerFlags/scaleFlags: options controlling mean-centering and variance scaling.
## NULL means no centering or scaling by default.
## A single TRUE/FALSE value applies to all cohorts.
## A TRUE/FALSE vector can be supplied to specify cohort-wise scaling settings.
## Example: centerFlags = c(FALSE, FALSE, FALSE, TRUE, TRUE) scales only the fourth and fifth cohorts.
## Named vectors are matched by cohort name rather than by order.
Train_set = scaleData(data = Train_expr, centerFlags = F, scaleFlags = F)
names(x = split(as.data.frame(Test_expr), f = Test_surv$Cohort)) # Ensure that validation cohort scaling follows this cohort order.
Test_set = scaleData(data = Test_expr, cohort = Test_surv$Cohort, centerFlags = F, scaleFlags = F)
# summary(apply(Train_set, 2, var))
# summary(apply(Test_set, 2, var))
# Check the scaling results.

# Model training and validation -------------------------------------------

## method list --------------------------------------------------------
# The method list records algorithm combinations in the format feature-selection method plus modeling method.
# Only StepCox and Enet accept method parameters in this prognostic script.
methods <- read.xlsx(file.path(code.path, "41467_2022_28421_MOESM4_ESM.xlsx"), startRow = 2)$Model
methods <- gsub("-| ", "", methods)

## Train the model --------------------------------------------------------
min.selected.var <- 5 # Set the minimum number of predictors required for a valid model.
timeVar = "OS.time"; statusVar = "OS" # Define the survival time and event-status variables.

## Pre-training
Variable = colnames(Train_expr)
preTrain.method =  strsplit(methods, "\\+")
preTrain.method = lapply(preTrain.method, function(x) rev(x)[-1])
preTrain.method = unique(unlist(preTrain.method))
preTrain.method

set.seed(seed = 123) # Set the random seed to ensure reproducibility.
preTrain.var <- list()
for (method in preTrain.method){
  preTrain.var[[method]] = RunML(method = method, # Machine-learning method.
                                 Train_expr = Train_set, # Candidate predictors in the training cohort.
                                 Train_surv = Train_surv, # Training survival data: rows are samples and outcome columns must include valid survival time and status.
                                 mode = "Variable", # Running mode: Variable for feature selection and Model for model fitting.
                                 classVar = classVar) # Survival variables used for training; they must be present in Train_surv.
}
preTrain.var[["simple"]] <- colnames(Train_expr)

model <- list() # Initialize the model list.
set.seed(seed = 123) # Set the random seed to ensure reproducibility.
for (method in methods){ # Loop through each algorithm combination.
# Example for debugging: assign the failing method and rerun from this step rather than restarting all methods.
  cat(match(method, methods), ":", method, "\n") # Print the current method.
  method_name = method # Name of the current algorithm combination.
  method <- strsplit(method, "\\+")[[1]] # Split the combination into feature-selection and modeling algorithms.

  if (length(method) == 1) method <- c("simple", method)

  selected.var = preTrain.var[[method[1]]]
# Discard this algorithm combination if the selected feature set is smaller than the minimum threshold.
  if (length(selected.var) <= min.selected.var) {
    model[[method_name]] <- NULL
  } else {
    model[[method_name]] <- RunML(method = method[2], # Machine-learning method.
```

```
                                  Train_expr = Train_expr[, selected.var], # Candidate predictors in the training cohort.
                                  Train_surv = Train_surv, # Training survival data: rows are samples and outcome columns must include valid survival time and status.
                                  mode = "Model", # Running mode: Variable for feature selection and Model for model fitting.
                                  classVar = classVar) # Survival variables used for training; they must be present in Train_surv.
  }

# Discard the fitted model if its final feature set is smaller than the minimum threshold.
  if(length(ExtractVar(model[[method_name]])) <= min.selected.var) {
    model[[method_name]] <- NULL
  }
}
saveRDS(model, file.path(res.path, "model.rds")) # Save all model outputs.

# If multiCox is selected, refit models as multivariable Cox models.
if (FinalModel == "multiCox"){
  coxmodel <- lapply(model, function(fit){ # Refit a multivariable Cox model using the variables selected by each algorithm to calculate sample-level risk scores.
    tmp <- coxph(formula = Surv(Train_surv[[timeVar]], Train_surv[[statusVar]]) ~ .,
                 data = as.data.frame(Train_set[, ExtractVar(fit)]))
    tmp$subFeature <- ExtractVar(fit) # Store final predictors after second-stage dimensionality reduction.
    return(tmp)
  })
}
saveRDS(coxmodel, file.path(res.path, "coxmodel.rds")) # Save the final multivariable Cox models fitted with selected variables.

## Evaluate the model -----------------------------------------------------

# Load saved model objects.
model <- readRDS(file.path(res.path, "model.rds")) # Use this line to calculate scores from the original algorithm-specific models.
# Use this line to calculate scores from the multivariable refitted model if needed.

methodsValid <- names(model) # Extract valid models; models with too few predictors are considered invalid.

# Calculate sample-level prediction scores from expression data.
RS_list <- list()
for (method in methodsValid){
  RS_list[[method]] <- CalRiskScore(fit = model[[method]],
                                    new_data = rbind.data.frame(Train_set,Test_set), # Version update.
                                    type = "lp") # Translated note from the original script.

}
RS_mat <- as.data.frame(t(do.call(rbind, RS_list)))
write.table(RS_mat, file.path(res.path, "RS_mat.txt"),sep = "\t", row.names = T, col.names = NA, quote = F) # Write the prediction/risk-score matrix.

# Extract selected features as a list.
fea_list <- list()
for (method in methodsValid) {
  fea_list[[method]] <- ExtractVar(model[[method]]) # Store final predictors after second-stage dimensionality reduction.
}

# Extract selected features as a data frame.
fea_df <- lapply(model, function(fit){ data.frame(ExtractVar(fit)) }) # Store final predictors after second-stage dimensionality reduction.
fea_df <- do.call(rbind, fea_df)
fea_df$algorithm <- gsub("(.+)\\.(.+$)", "\\1", rownames(fea_df))
colnames(fea_df)[1] <- "features" # The data frame contains algorithm names and selected features.
write.table(fea_df, file.path(res.path, "fea_df.txt"),sep = "\t", row.names = F, col.names = T, quote = F)

# Evaluate model performance by C-index or cohort-specific metrics.
Cindexlist <- list()
for (method in methodsValid){
  Cindexlist[[method]] <- RunEval(fit = model[[method]], # Prognostic model.
                                  Test_expr = Test_set, # Translated note from the original script.
                                  Test_surv = Test_surv, # Training survival data: rows are samples and outcome columns must include valid survival time and status.
# Training expression matrix: rows are features and columns are samples. Feature identifiers should be consistent with the validation matrix.
# Training survival data: rows are samples and outcome columns must include valid survival time and status.
# Provide a training cohort label if the training cohort should also be evaluated; otherwise it is labeled as Training.
                                  Train_expr = NULL,
                                  Train_surv = NULL,
                                  cohortVar = "Cohort", # Important: specify the cohort indicator variable; this column must exist.
                                  timeVar = timeVar, # Survival time variable used for evaluation.
                                  statusVar = statusVar) # Survival event-status variable used for evaluation.
```

```r
}
Cindex_mat <- do.call(rbind, Cindexlist)
write.table(Cindex_mat, file.path(res.path, "cindex_mat.txt"),sep = "\t", row.names = T, col.names = T, quote = F)

# Plot -------------------------------------------------------------------



## =========================================================
## Load required R packages.
## =========================================================
## Load required R packages.
## =========================================================
library(ComplexHeatmap)
library(circlize)
library(RColorBrewer)
library(grid)

## =========================================================
## Read and prepare the performance matrix.
## =========================================================
Cindex_mat <- read.table(file.path(res.path, "cindex_mat.txt"),
                         sep = "\t",
                         row.names = 1,
                         header = TRUE,
                         check.names = FALSE,
                         stringsAsFactors = FALSE)

# Calculate the average C-index across cohorts and sort algorithms in descending order.
avg_Cindex <- sort(apply(Cindex_mat, 1, mean, na.rm = TRUE),
                   decreasing = TRUE)

# Reorder the matrix according to the average performance.
Cindex_mat <- Cindex_mat[names(avg_Cindex), ]

# Format the average C-index to three decimal places.
avg_Cindex <- as.numeric(format(avg_Cindex,
                                digits = 3,
                                nsmall = 3))

# Retain selected features from the best-performing model.
fea_sel <- fea_list[[rownames(Cindex_mat)[1]]]

# Create numeric and text matrices for heatmap cell labels.
Cindex_mat_num <- as.matrix(Cindex_mat)
text_mat <- matrix(sprintf("%.3f", Cindex_mat_num),
                   nrow = nrow(Cindex_mat_num),
                   dimnames = dimnames(Cindex_mat_num))

## =========================================================
## Publication-style color palette.
## =========================================================
# Main heatmap color scale.
col_fun <- colorRamp2(
  c(0.50, 0.60, 0.70, 0.80),
  c("#f7f4e9", # warm ivory
    "#cfe6de", # light cyan green
    "#6ba48f", # cyan green
    "#e3a23b") # cinnabar yellow
)

# Cohort colors based on the Paired palette.
if (ncol(Cindex_mat) <= 12) {
  CohortCol <- brewer.pal(n = max(3, ncol(Cindex_mat)), name = "Paired")[1:ncol(Cindex_mat)]
} else {
  CohortCol <- colorRampPalette(brewer.pal(12, "Paired"))(ncol(Cindex_mat))
}
names(CohortCol) <- colnames(Cindex_mat)

top_ha <- HeatmapAnnotation(
```

```r
  cohort = colnames(Cindex_mat),
  col = list(cohort = CohortCol),
  annotation_name_side = "left",
  annotation_name_gp   = gpar(fontsize = 13, fontface = "bold"),
  annotation_legend_param = list(
    title     = "Cohort",
    title_gp  = gpar(fontsize = 3, fontface = "bold"),
    labels_gp = gpar(fontsize = 1)
  )
)

## ========================================================
## Left C-index panel with a uniform bar and large white values.
## ========================================================
Cindex_left_mat <- matrix(
  1,
  nrow = length(avg_Cindex),
  ncol = 1,
  dimnames = list(rownames(Cindex_mat), "C-index")
)

ht_left <- Heatmap(
  Cindex_left_mat,
  name = "Cindex_left",
  col = "#6f7a4b", # Uniform dark green.
  show_heatmap_legend = FALSE,
  cluster_rows = FALSE,
  cluster_columns = FALSE,
  width = unit(24, "mm"), # Left-panel width.
  show_row_names = FALSE, # Display row names on the right-side main heatmap.
  column_title = NULL, # Remove the title to avoid layout misalignment.
  cell_fun = function(j, i, x, y, width, height, fill) {
# Write the average value inside the bar in large white text.
    grid.text(
      sprintf("%.3f", avg_Cindex[i]),
      x = x, y = y,
      gp = gpar(col = "white",
                fontsize = 15,
                fontface = "bold")
    )
  }
)

## ========================================================
## Main heatmap showing cohort-specific values.
## ========================================================
ht_right <- Heatmap(
  Cindex_mat_num,
  name = "C-index",
  col = col_fun,
  cluster_rows = FALSE,
  cluster_columns = FALSE,
  rect_gp = gpar(col = "white", lwd = 0.5),
  show_heatmap_legend = FALSE, # Hide the C-index legend.

# Display row names on the right with larger font.
  row_names_side = "right",
  row_names_gp   = gpar(fontsize = 15),

# Horizontal labels.
  column_names_side     = "top",
  column_names_gp       = gpar(fontsize = 13, fontface = "bold"),
  column_names_rot      = 0,
  column_names_centered = TRUE,

  top_annotation = top_ha,

  heatmap_legend_param = list(
    title     = "C-index",
    title_gp  = gpar(fontsize = 14, fontface = "bold"),
```

```r
    labels_gp = gpar(fontsize = 14),
    at        = seq(0.50, 0.80, 0.05),
    labels    = sprintf("%.2f", seq(0.50, 0.80, 0.05)),
    legend_height = unit(52, "mm")
  ),

# Write the C-index value in each heatmap cell.
  cell_fun = function(j, i, x, y, width, height, fill) {
    grid.text(
      text_mat[i, j],
      x = x, y = y,
      gp = gpar(fontsize = 14, fontface = "bold")
    )
  }
)

## ========================================================
## Export the PDF figure.
## ========================================================
# Slightly enlarge cell size to accommodate larger font.
cellwidth  <- 0.72
cellheight <- 0.42

pdf_width  <- cellwidth * ncol(Cindex_mat) + 7.5
pdf_height <- cellheight * nrow(Cindex_mat) + 3.6

pdf(file.path(fig.path, "Cindex_CNstyle_leftBar_bigFont.pdf"),
    width  = pdf_width,
    height = pdf_height,
    useDingbats = FALSE)

draw(
  ht_left + ht_right,
  heatmap_legend_side     = "right",
  annotation_legend_side  = "right",
# Add margins around the plot.
  padding = unit(c(6, 6, 6, 6), "mm") # Top, right, bottom, left.
)

dev.off()





## ========================================================
## Load required R packages.
## ========================================================
library(ComplexHeatmap)
library(circlize)
library(RColorBrewer)
library(grid)

## ========================================================
## Read and prepare the performance matrix.
## ========================================================
Cindex_mat <- read.table(file.path(res.path, "cindex_mat.txt"),
                         sep = "\t",
                         row.names = 1,
                         header = TRUE,
                         check.names = FALSE,
                         stringsAsFactors = FALSE)

# Calculate the average C-index across cohorts and sort algorithms in descending order.
avg_Cindex <- sort(apply(Cindex_mat, 1, mean, na.rm = TRUE),
                   decreasing = TRUE)

# Reorder the matrix according to the average performance.
Cindex_mat <- Cindex_mat[names(avg_Cindex), ]
```

```r
# Format the average C-index to three decimal places.
avg_Cindex <- as.numeric(format(avg_Cindex,
                                digits = 3,
                                nsmall = 3))

# Retain selected features from the best-performing model.
fea_sel <- fea_list[[rownames(Cindex_mat)[1]]]

# Create numeric and text matrices for heatmap cell labels.
Cindex_mat_num <- as.matrix(Cindex_mat)
text_mat <- matrix(sprintf("%.3f", Cindex_mat_num),
                   nrow = nrow(Cindex_mat_num),
                   dimnames = dimnames(Cindex_mat_num))

## ========================================================
## Publication-style color palette.
## ========================================================
# Main heatmap color scale.
col_fun <- colorRamp2(
  c(0.50, 0.60, 0.70, 0.80),
  c("#f7f4e9", # warm ivory
    "#cfe6de", # light cyan green
    "#6ba48f", # cyan green
    "#e3a23b") # cinnabar yellow
)

# Cohort colors based on the Paired palette.
if (ncol(Cindex_mat) <= 12) {
  CohortCol <- brewer.pal(n = max(3, ncol(Cindex_mat)), name = "Paired")[1:ncol(Cindex_mat)]
} else {
  CohortCol <- colorRampPalette(brewer.pal(12, "Paired"))(ncol(Cindex_mat))
}
names(CohortCol) <- colnames(Cindex_mat)

top_ha <- HeatmapAnnotation(
  cohort = colnames(Cindex_mat),
  col = list(cohort = CohortCol),
  annotation_name_side = "left",
  annotation_name_gp   = gpar(fontsize = 13, fontface = "bold"),
# Hide the cohort legend.
  show_legend = FALSE
)

## ========================================================
## Left C-index panel with a uniform bar and large white values.
## ========================================================
Cindex_left_mat <- matrix(
  1,
  nrow = length(avg_Cindex),
  ncol = 1,
  dimnames = list(rownames(Cindex_mat), "C-index")
)

ht_left <- Heatmap(
  Cindex_left_mat,
  name = "Cindex_left",
  col = "#6f7a4b", # Uniform dark green.
  show_heatmap_legend = FALSE, # No legend is required for this panel.
  cluster_rows = FALSE,
  cluster_columns = FALSE,
  width = unit(24, "mm"), # Left-panel width.
  show_row_names = FALSE, # Display row names on the right-side main heatmap.
  column_title = NULL, # Remove the title to avoid layout misalignment.
  cell_fun = function(j, i, x, y, width, height, fill) {
# Write the average value inside the bar in large white text.
    grid.text(
      sprintf("%.3f", avg_Cindex[i]),
      x = x, y = y,
      gp = gpar(col = "white",
                fontsize = 15,
```

```r
                fontface = "bold")
    )
  }
)

## ========================================================
## Main heatmap showing cohort-specific values.
## ========================================================
ht_right <- Heatmap(
  Cindex_mat_num,
  name = "C-index",
  col = col_fun,
  show_heatmap_legend = FALSE, # Hide the C-index legend.
  cluster_rows = FALSE,
  cluster_columns = FALSE,
  rect_gp = gpar(col = "white", lwd = 0.5),

# Display row names on the right with larger font.
  row_names_side = "right",
  row_names_gp   = gpar(fontsize = 15),

# Place column names at the top, horizontally and centered.
  column_names_side     = "top",
  column_names_gp       = gpar(fontsize = 13, fontface = "bold"),
  column_names_rot      = 0,
  column_names_centered = TRUE,

  top_annotation = top_ha,

# Defined here but not displayed because show_heatmap_legend is FALSE.
  heatmap_legend_param = list(
    title     = "C-index",
    title_gp  = gpar(fontsize = 13, fontface = "bold"),
    labels_gp = gpar(fontsize = 11),
    at        = seq(0.50, 0.80, 0.05),
    labels    = sprintf("%.2f", seq(0.50, 0.80, 0.05)),
    legend_height = unit(52, "mm")
  ),

# Write the C-index value in each heatmap cell.
  cell_fun = function(j, i, x, y, width, height, fill) {
    grid.text(
      text_mat[i, j],
      x = x, y = y,
      gp = gpar(fontsize = 14, fontface = "bold")
    )
  }
)

## ========================================================
## Export the PDF figure.
## ========================================================
# Cell size.
cellwidth  <- 0.72
cellheight <- 0.42

pdf_width  <- cellwidth * ncol(Cindex_mat) + 7.5
pdf_height <- cellheight * nrow(Cindex_mat) + 3.6

pdf(file.path(fig.path, "Cindex_CNstyle_leftBar_noLegend.pdf"),
    width  = pdf_width,
    height = pdf_height,
    useDingbats = FALSE)

draw(
  ht_left + ht_right,
# Do not manually specify legend placement.
  padding = unit(c(6, 6, 6, 6), "mm") # Top, right, bottom, left.
)
```

```
dev.off()
```

Part IV. Prognostic Machine Learning Functions

```
# all ML algorithms
RunML <- function(method, Train_expr, Train_surv, mode = "Model", timeVar = "OS.time", statusVar = "OS", ...){
  # for example: Enet [alpha=0.4]
  method = gsub(" ", "", method) # Remove spaces from method parameters, e.g., Enet [alpha=0.4].
  method_name = gsub("(\\w+)\\[(.+)\\]", "\\1", method)  # get name of ML algorithm, e.g., Enet
  method_param = gsub("(\\w+)\\[(.+)\\]", "\\2", method) # get parameter of ML algorithm, e.g., alpha=0.4

  method_param = switch(
    EXPR = method_name,
    "Enet" = list("alpha" = as.numeric(gsub("alpha=", "", method_param))),
    "StepCox" = list("direction" = method_param),
    NULL
  )
  message("Run ", method_name, " algorithm for ", mode, "; ",
          method_param, ";",
          " using ", ncol(Train_expr), " Variables")

  args = list("Train_expr" = Train_expr,
              "Train_surv" = Train_surv,
              "mode" = mode,
              "timeVar" = timeVar, "statusVar" = statusVar)
  args = c(args, method_param)

  obj <- do.call(what = paste0("Run", method_name),
                 args = args)

  if(mode == "Variable"){
    message(length(obj), " Variables retained;\n")
  }else{message("\n")}
  return(obj)
}

RunEnet <- function(Train_expr, Train_surv, mode, timeVar, statusVar, alpha){
  cv.fit = cv.glmnet(x = Train_expr,
                     y = Surv(Train_surv[[timeVar]], Train_surv[[statusVar]]),
                     family = "cox", alpha = alpha, nfolds = 10)
  fit = glmnet(x = Train_expr,
               y = Surv(Train_surv[[timeVar]], Train_surv[[statusVar]]),
               family = "cox", alpha = alpha, lambda = cv.fit$lambda.min)
  fit$subFeature = colnames(Train_expr)
  if (mode == "Model") return(fit)
  if (mode == "Variable") return(ExtractVar(fit))
}

RunLasso <- function(Train_expr, Train_surv, mode, timeVar, statusVar){
  RunEnet(Train_expr, Train_surv, mode, timeVar, statusVar, alpha = 1)
}

RunRidge <- function(Train_expr, Train_surv, mode, timeVar, statusVar){
  RunEnet(Train_expr, Train_surv, mode, timeVar, statusVar, alpha = 0)
}

RunStepCox <- function(Train_expr, Train_surv, mode, timeVar, statusVar, direction){
  fit <- step(coxph(formula = Surv(Train_surv[[timeVar]], Train_surv[[statusVar]]) ~ .,
                    data = as.data.frame(Train_expr)),
              direction = direction, trace = 0)
  fit$subFeature = colnames(Train_expr)
  if (mode == "Model") return(fit)
  if (mode == "Variable") return(ExtractVar(fit))
}

RunsurvivalSVM <- function(Train_expr, Train_surv, mode, timeVar, statusVar){
  fit = survivalsvm(formula = Surv(Train_surv[[timeVar]], Train_surv[[statusVar]]) ~ .,
                    data= as.data.frame(Train_expr),
                    gamma.mu = 1, opt.meth = "ipop")
  fit$subFeature = colnames(Train_expr)
  if (mode == "Model") return(fit)
  if (mode == "Variable") return(ExtractVar(fit))
```

```
}

RunCoxBoost <- function(Train_expr, Train_surv, mode, timeVar, statusVar){
  pen <- optimCoxBoostPenalty(time = Train_surv[[timeVar]],
                              status = Train_surv[[statusVar]],
                              x = Train_expr,
                              trace = F, start.penalty=500, parallel = F)
  cv.res <- cv.CoxBoost(time = Train_surv[[timeVar]],
                        status = Train_surv[[statusVar]],
                        x = Train_expr,
                        maxstepno=500, K=10, type="verweij", penalty=pen$penalty)
  fit <- CoxBoost(time = Train_surv[[timeVar]],
                  status = Train_surv[[statusVar]],
                  x = Train_expr,
                  stepno = cv.res$optimal.step,
                  penalty = pen$penalty)
  fit$subFeature = colnames(Train_expr)
  if (mode == "Model") return(fit)
  if (mode == "Variable") return(ExtractVar(fit))
}

RunSuperPC <- function(Train_expr, Train_surv, mode, timeVar, statusVar){
  data <- list(x = t(scale(Train_expr)),
               y = Train_surv[[timeVar]],
               censoring.status = Train_surv[[statusVar]],
               featurenames = colnames(Train_expr))
  fit <- superpc.train(data = data,type = 'survival',s0.perc = 0.5) # default
  cv.fit <- suppressWarnings(superpc.cv(fit, data,
                                        n.threshold = 20, # default
                                        n.fold = 10,
                                        n.components = 3,
                                        min.features = 5,
                                        max.features = nrow(data$x),
                                        compute.fullcv = TRUE,
                                        compute.preval = TRUE))
  fit$threshold <- cv.fit$thresholds[which.max(cv.fit[["scor"]][1,])]
  fit$data <- data
  fit$subFeature = colnames(Train_expr)
  if (mode == "Model") return(fit)
  if (mode == "Variable") return(ExtractVar(fit))
}

RunplsRcox <- function(Train_expr, Train_surv, mode, timeVar, statusVar){
  data <- list(x = Train_expr,
               time = Train_surv[[timeVar]],
               status = Train_surv[[statusVar]])
  cv.plsRcox.res = cv.plsRcox(data = data,
                              nt=10, verbose = FALSE)
  fit <- plsRcox(Xplan = data$x,
                 time = data$time,
                 event = data$status,
                 nt = as.numeric(cv.plsRcox.res[5]),
                 verbose = F, sparse = T)
  fit$subFeature = colnames(Train_expr)
  if (mode == "Model") return(fit)
  if (mode == "Variable") return(ExtractVar(fit))
}

RunRSF <- function(Train_expr, Train_surv, mode, timeVar, statusVar){
  rf_nodesize = 5 # If RSF does not return an effective feature-selection result, tune the parameters iteratively.
  fit <- rfsrc(formula = formula(paste0("Surv(", timeVar, ", ", statusVar, ")", "~.")),
               data = cbind(Train_expr, Train_surv[,c(timeVar, statusVar)]), # Version update.
               ntree = 1000, nodesize = rf_nodesize,
               splitrule = 'logrank',
               importance = T,
               proximity = T,
               forest = T)
  fit$subFeature = colnames(Train_expr)
  if (mode == "Model") return(fit)
  if (mode == "Variable") return(ExtractVar(fit))
```

```
}

RunGBM <- function(Train_expr, Train_surv, mode, timeVar, statusVar){
  fit <- gbm(formula = Surv(Train_surv[[timeVar]], Train_surv[[statusVar]]) ~ .,
             data = as.data.frame(Train_expr),
             distribution = 'coxph',
             n.trees = 10000,
             interaction.depth = 3,
             n.minobsinnode = 10,
             shrinkage = 0.001,
             cv.folds = 10,n.cores = 6)
  best <- which.min(fit$cv.error)
  fit <- gbm(formula = Surv(Train_surv[[timeVar]], Train_surv[[statusVar]]) ~ .,
             data = as.data.frame(Train_expr),
             distribution = 'coxph',
             n.trees = best,
             interaction.depth = 3,
             n.minobsinnode = 10,
             shrinkage = 0.001, n.cores = 8)
  fit$subFeature = colnames(Train_expr)
  if (mode == "Model") return(fit)
  if (mode == "Variable") return(ExtractVar(fit))
}

quiet <- function(..., messages=FALSE, cat=FALSE){
  if(!cat){
    sink(tempfile())
    on.exit(sink())
  }
  out <- if(messages) eval(...) else suppressMessages(eval(...))
  out
}

ExtractVar <- function(fit){
  Feature <- quiet(switch(
    EXPR = class(fit)[1],
    "coxnet" = rownames(coef(fit))[which(coef(fit)[, 1] != 0)], # This model does not directly perform feature selection; features with zero coefficients are removed where applicable.
    "coxph" = names(coef(fit)), # Stepwise regression can perform variable selection.
    "survivalsvm" = fit$var.names, # SVM does not perform feature selection, so all input features are retained by default.
    "CoxBoost" = names(coef(fit)[abs(coef(fit)) > 0]), # CoxBoost does not directly perform feature selection; variables with nonzero coefficients are retained.
    "superpc" = names(fit$feature.scores)[abs(fit$feature.scores) > 0.5], # SuperPC does not directly perform feature selection; features with absolute scores greater than 0.5 are retained.
    "plsRcoxmodel" = rownames(fit$Coeffs)[fit$Coeffs != 0], # plsRcox does not directly perform feature selection; variables with nonzero coefficients are retained.
    "rfsrc" = var.select(fit, verbose = F)$topvars, # rfsrc can perform variable selection.
    "gbm" = rownames(summary.gbm(fit, plotit = FALSE))[summary.gbm(fit, plotit = FALSE)$rel.inf > 0] # GBM performs feature selection by retaining variables with nonzero relative influence.
  ))
  return(Feature)
}

CalRiskScore <- function(fit, new_data, type = "lp"){
  new_data <- new_data[, fit$subFeature]
  RS <- quiet(switch(
    EXPR = class(fit)[1],
    "coxnet"      = predict(fit, type = 'link', as.matrix(new_data)),
    "coxph"       = predict(fit, type = 'lp', as.data.frame(new_data)),
    "survivalsvm" = predict(fit, as.data.frame(new_data))$predicted,
    "CoxBoost"    = predict(fit, type = "lp", as.data.frame(new_data)),
    "superpc"     = superpc.predict(object = fit,
                                    data = fit$data,
                                    newdata = list(x = t(scale(as.matrix(new_data)))),
                                    threshold = fit$threshold,
                                    n.components = 1)$v.pred,
    "plsRcoxmodel" = predict(fit, type = "lp", as.data.frame(new_data)),
    "rfsrc"        = predict(fit, as.data.frame(new_data))$predicted,
    "gbm"          = predict(fit, type = 'link', as.data.frame(new_data))
  ))
  RS = as.vector(RS)
  names(RS) = rownames(new_data)
  return(RS)
}
```

```
RunEval <- function(fit,
                    Test_expr = NULL,
                    Test_surv = NULL,
                    Train_expr = NULL,
                    Train_surv = NULL,
                    Train_name = NULL,
                    cohortVar = "Cohort",
                    timeVar = "OS.time",
                    statusVar = "OS"){

  if(!is.element(cohortVar, colnames(Test_surv))) {
    stop(paste0("There is no [", cohortVar, "] indicator, please fill in one more column!"))
  }

  if((!is.null(Train_expr)) & (!is.null(Train_surv))) {
    new_data <- rbind.data.frame(Train_expr[, fit$subFeature],
                                 Test_expr[, fit$subFeature])

    if(!is.null(Train_name)) {
      Train_surv$Cohort <- Train_name
    } else {
      Train_surv$Cohort <- "Training"
    }
    colnames(Train_surv)[ncol(Train_surv)] <- cohortVar
    Test_surv <- rbind.data.frame(Train_surv[,c(cohortVar, timeVar, statusVar)],
                                  Test_surv[,c(cohortVar, timeVar, statusVar)])
    Test_surv[,1] <- factor(Test_surv[,1],
                            levels = c(unique(Train_surv[,cohortVar]), setdiff(unique(Test_surv[,cohortVar]),unique(Train_surv[,cohortVar]))))
  } else {
    new_data <- Test_expr[, fit$subFeature]
  }

  RS <- CalRiskScore(fit = fit, new_data = new_data)

  Predict.out <- Test_surv
  Predict.out$RS <- as.vector(RS)
  Predict.out <- split(x = Predict.out, f = Predict.out[,cohortVar])
  f <- as.formula(paste0("Surv(", timeVar,",",statusVar,")~RS"))
  unlist(lapply(Predict.out, function(data){
    unname(summary(coxph(formula = f,
                         data = data))$concordance["C"])
  }))
}

SimpleHeatmap <- function(Cindex_mat = NULL,
                          avg_Cindex = NULL,
                          CohortCol = NULL,
                          barCol = NULL,
                          col = c("#4195C1", "#FFFFFF", "#CB5746"), # Blue-red palette.
                          cellwidth = 1, cellheight = 0.5,
                          cluster_columns, cluster_rows){
  col_ha = columnAnnotation("Cohort" = colnames(Cindex_mat),
                            col = list("Cohort" = CohortCol),
                            show_annotation_name = F)

  row_ha = rowAnnotation(bar = anno_barplot(avg_Cindex, bar_width = 0.8, border = FALSE,
                                            gp = gpar(fill = barCol, col = NA),
                                            add_numbers = T, numbers_offset = unit(-10, "mm"),
                                            axis_param = list("labels_rot" = 0),
                                            numbers_gp = gpar(fontsize = 9, col = "white"),
                                            width = unit(3, "cm")),
                         show_annotation_name = F)

  Heatmap(as.matrix(Cindex_mat), name = "C-index",
          right_annotation = row_ha,
          top_annotation = col_ha,
          col = col,
          rect_gp = gpar(col = "black", lwd = 1), # Use black cell borders.
          cluster_columns = cluster_columns, cluster_rows = cluster_rows, # No clustering is performed because it is not meaningful here.
          show_column_names = FALSE,
```

```
          show_row_names = TRUE,
          row_names_side = "left",
          width = unit(cellwidth * ncol(Cindex_mat) + 2, "cm"),
          height = unit(cellheight * nrow(Cindex_mat), "cm"),
          column_split = factor(colnames(Cindex_mat), levels = colnames(Cindex_mat)),
          column_title = NULL,
          cell_fun = function(j, i, x, y, w, h, col) { # add text to each grid
            grid.text(label = format(Cindex_mat[i, j], digits = 3, nsmall = 3),
                      x, y, gp = gpar(fontsize = 10))
          }
  )
}

# Version update.
standarize.fun <- function(indata, centerFlag, scaleFlag) {
  scale(indata, center=centerFlag, scale=scaleFlag)
}

# Version update.
scaleData <- function(data, cohort = NULL, centerFlags = NULL, scaleFlags = NULL){
  samplename = rownames(data)
  if (is.null(cohort)){
    data <- list(data); names(data) = "training"
  }else{
    data <- split(as.data.frame(data), cohort)
  }

  if (is.null(centerFlags)){
    centerFlags = F; message("No centerFlags found, set as FALSE")
  }
  if (length(centerFlags)==1){
    centerFlags = rep(centerFlags, length(data)); message("set centerFlags for all cohort as ", unique(centerFlags))
  }
  if (is.null(names(centerFlags))){
    names(centerFlags) <- names(data); message("match centerFlags with cohort by order\n")
  }

  if (is.null(scaleFlags)){
    scaleFlags = F; message("No scaleFlags found, set as FALSE")
  }
  if (length(scaleFlags)==1){
    scaleFlags = rep(scaleFlags, length(data)); message("set scaleFlags for all cohort as ", unique(scaleFlags))
  }
  if (is.null(names(scaleFlags))){
    names(scaleFlags) <- names(data); message("match scaleFlags with cohort by order\n")
  }

  centerFlags <- centerFlags[names(data)]; scaleFlags <- scaleFlags[names(data)]
  outdata <- mapply(standarize.fun, indata = data, centerFlag = centerFlags, scaleFlag = scaleFlags, SIMPLIFY = F)
  # lapply(out.data, function(x) summary(apply(x, 2, var)))
  outdata <- do.call(rbind, outdata)
  outdata <- outdata[samplename, ]
  return(outdata)
}
```

Reproducible Machine-Learning Workflow and Parameter Summary

Diagnostic model workflow

Item	Description
Input feature source	Candidate genes from subtype-associated DEGs intersected with fibroblast-specific hdWGCNA hub genes; input matrices were restricted to shared genes between training and validation cohorts.
Training cohort	GSE47460.
Independent validation cohorts	GSE32537, GSE53845, and GSE150910. External validation cohorts were not used for feature selection, tuning, model fitting, or model selection.
Outcome variable	Binary diagnostic outcome coded as 0/1 (classVar = "outcome").
Algorithm-combination strategy	Two-stage pipeline: first-stage feature selection followed by second-stage diagnostic classifier construction. A total of 113 combinations were evaluated.
Minimum feature threshold	Models retaining <=5 predictors were excluded to avoid unstable signatures.
Internal cross-validation and tuning	Performed inside the training cohort where supported by each package: glmnet used 10-fold CV for lambda; plsRglm used cross-validation with nt = 10; GBM used 10-fold CV to select the optimal number of trees; XGBoost used 5-fold CV to select nrounds; glmBoost used cross-validated risk for mstop; LDA used caret CV.
Nested cross-validation	Not applied in the original workflow. Instead, overfitting was controlled using training-only feature selection, internal CV-based tuning, minimum-feature filtering, parsimonious model selection, and strictly independent external validation.
Final model type	The script can preserve algorithm-specific models (panML) or refit selected variables using multivariable logistic regression (multiLogistic).
Primary performance metric	Area under the receiver operating characteristic curve (AUC).
Main result files	model.rds, logisticmodel.rds, RS_mat.txt, Class_mat.txt, fea_df.txt, AUC_mat.txt, and AUC heatmap PDF.
Random seed	777.

Prognostic model workflow

Item	Description
Input feature source	Candidate genes from subtype-associated DEGs intersected with fibroblast-specific hdWGCNA hub genes; input matrices were restricted to shared genes between training and validation cohorts.
Training cohort	GSE28042.
Independent validation cohorts	GSE27957 and GSE93606. External validation cohorts were not used for feature selection, tuning, model fitting, or model selection.
Outcome variable	Survival time and status (timeVar = "OS.time"; statusVar = "OS").
Algorithm-combination strategy	Two-stage pipeline: first-stage feature selection followed by second-stage survival-model construction. A total of 101 combinations were evaluated.
Minimum feature threshold	Models retaining <=5 predictors were excluded to avoid unstable signatures.
Internal cross-validation and tuning	Performed inside the training cohort where supported by each package: glmnet used 10-fold CV for lambda; CoxBoost used penalty optimization and 10-fold CV for the optimal boosting step; SuperPC used 10-fold CV; plsRcox used cross-validation with nt = 10; GBM used 10-fold CV to select the optimal number of trees.
Nested cross-validation	Not applied in the original workflow. Instead, overfitting was controlled using training-only feature selection, internal CV-based tuning, minimum-feature filtering, parsimonious model selection, and strictly independent external validation.
Final model type	The script can preserve algorithm-specific models (panML) or refit selected variables using multivariable Cox regression (multiCox).
Primary performance metric	Concordance index (C-index).
Main result files	model.rds, coxmodel.rds, RS_mat.txt, fea_df.txt, cindex_mat.txt, and C-index heatmap PDF.
Random seed	123.

Algorithm-Specific Parameter and Tuning Details

Diagnostic algorithms

Algorithm	Implementation and tuning details
LASSO / Ridge / Elastic Net	glmnet binomial model; alpha = 1 for LASSO, alpha = 0 for Ridge, and specified alpha for Enet; lambda selected by 10-fold cv.glmnet.
Stepglm	Stepwise binomial generalized linear model; direction defined in the method list (e.g., both/backward/forward).
SVM	e1071 SVM with probability = TRUE; package defaults used unless otherwise specified.
LDA	caret::train with method = "lda" and cross-validation control.
glmBoost	mboost::glmboost with Binomial family; mstop determined using cross-validated risk and constrained to at least 40.
plsRglm	cv.plsRglm with nt = 10; final model fitted as sparse PLS logistic model.
Random forest	randomForestSRC classification forest with ntree = 1000 and nodesize = 5; variable selection by var.select.
GBM	Bernoulli GBM with n.trees = 10000, interaction.depth = 3, n.minobsinnode = 10, shrinkage = 0.001, and 10-fold CV to select the optimal number of trees.
XGBoost	Binary logistic XGBoost; max.depth = 2, eta = 1, nthread = 2; 5-fold CV used to select nrounds.
Naive Bayes	e1071::naiveBayes using package defaults.

Prognostic algorithms

Algorithm	Implementation and tuning details
LASSO / Ridge / Elastic Net	glmnet Cox model; alpha = 1 for LASSO, alpha = 0 for Ridge, and specified alpha for Enet; lambda selected by 10-fold cv.glmnet.
StepCox	Stepwise Cox proportional hazards model; direction defined in the method list (e.g., both/backward/forward).
survivalSVM	survivalsvm with gamma.mu = 1 and opt.meth = "ipop".
CoxBoost	Penalty optimized using optimCoxBoostPenalty; optimal step selected by 10-fold cv.CoxBoost with maxstepno = 500.
SuperPC	superpc survival model with s0.perc = 0.5; 10-fold CV, n.threshold = 20, n.components = 3, min.features = 5; threshold selected by maximum CV score.
plsRcox	cv.plsRcox with nt = 10; final model fitted as sparse PLS-Cox model using the selected number of components.
RSF	randomForestSRC survival forest with ntree = 1000, nodesize = 5, and splitrule = "logrank"; variable selection by var.select.
GBM	CoxPH GBM with n.trees = 10000, interaction.depth = 3, n.minobsinnode = 10, shrinkage = 0.001, and 10-fold CV to select the optimal number of trees.

Model development was performed using a strictly separated training-validation strategy. For diagnostic modeling, GSE47460 was used as the training cohort, whereas GSE32537, GSE53845, and GSE150910 were used only as independent external validation cohorts. For prognostic modeling, GSE28042 was used as the training cohort, and GSE27957 and GSE93606 were retained as independent external validation cohorts. No samples from the external validation cohorts were used during feature selection, hyperparameter tuning, model fitting, or model selection. Feature selection was performed within the training cohort for each candidate algorithm combination, followed by model fitting using the corresponding selected feature set. Hyperparameter optimization was conducted within the training cohort using internal cross-validation where supported by each algorithm. Nested cross-validation was not applied in the original workflow because the study design prioritized independent external validation across multiple GEO cohorts. To reduce overfitting, dimensionality reduction before modeling, training-only feature selection, internal cross-validation, exclusion of unstable models with very small feature sets, parsimonious model selection, and independent external validation were adopted.
