# Supplementary material for: Integrative Multi-omics and machine learning analysis of sphingolipid-associated molecular stratification identifies diagnostic and prognostic signatures in idiopathic pulmonary fibrosis
Source: Orphanet J Rare Dis. 2026 Aug 25;21:384. doi: 10.1186/s13023-026-04563-4 (PMC13733085; doi:10.1186/s13023-026-04563-4)
Supplement: Supplementary file 3 [file 13023_2026_4563_MOESM3_ESM.docx]

**Supplementary Table 1. Basic characteristics of the included datasets**

| **Dataset** | **Country** | **Platform** | **Sample Source** | **No. of IPF Cases** | **No. of Control Cases** | **Submission date** |
| --- | --- | --- | --- | --- | --- | --- |
| GSE32537 | USA | GPL6244 [HuGene-1_0-st] Affymetrix Human Gene 1.0 ST Array | lung | 119 | 50 | 2011 |
| GSE53845 | USA | GPL6480 Agilent-014850 Whole Human Genome Microarray 4x44K G4112F | lung | 40 | 8 | 2014 |
| GSE150910 | USA | GPL24676 Illumina NovaSeq 6000 (Homo sapiens) | lung | 103 | 103 | 2020 |
| GSE47460 | USA | GPL6480 Agilent-014850 Whole Human Genome Microarray 4x44K G4112F (Probe Name version) and GPL14550 Agilent-028004 SurePrint G3 Human GE 8x60K Microarray (Probe Name Version) | lung | 160 | 108 | 2013 |
| GSE70866 | Germany | GPL14550 Agilent-028004 SurePrint G3 Human GE 8x60K Microarray and GPL17077 Agilent-039494 SurePrint G3 Human GE v2 8x60K Microarray 039381 | bronchoalveolar lavage | 176 | 20 | 2015 |
| GSE28042 | USA | GPL6480 Agilent-014850 Whole Human Genome Microarray 4x44K G4112F (Probe Name version) | peripheral blood mononuclear cells | 75 | 0 | 2013 |
| GSE27957 | USA | GPL5175 [HuEx-1_0-st] Affymetrix Human Exon 1.0 ST Array [transcript (gene) version] | peripheral blood mononuclear cells | 45 | 0 | 2013 |
| GSE93606 | United Kingdom | GPL11532 [HuGene-1_1-st] Affymetrix Human Gene 1.1 ST Array | whole blood | 57 | 0 | 2017 |
| GSE110147 | Canada | GPL6244 [HuGene-1_0-st] Affymetrix Human Gene 1.0 ST Array [transcript (gene) version] | lung | 22 | 11 | 2018 |
| GSE24206 | USA | GPL570 [HG-U133_Plus_2] Affymetrix Human Genome U133 Plus 2.0 Array | lung | 17 | 6 | 2010 |
| GSE68239 | Germany | GPL1708 Agilent-012391 Whole Human Genome Oligo Microarray G4112A (Feature Number version) | lung | 10 | 10 | 2015 |
| GSE92592 | USA | GPL11154 Illumina HiSeq 2000 (Homo sapiens) | lung | 20 | 19 | 2016 |

Note: The sample numbers refer to the samples used in this study. The added external transcriptomic datasets used for expression meta-analysis were GSE110147, GSE24206, GSE68239, and GSE92592.
