# Supplementary material for: Integrative Multi-omics and machine learning analysis of sphingolipid-associated molecular stratification identifies diagnostic and prognostic signatures in idiopathic pulmonary fibrosis
Source: Orphanet J Rare Dis. 2026 Aug 25;21:384. doi: 10.1186/s13023-026-04563-4 (PMC13733085; doi:10.1186/s13023-026-04563-4)
Supplement: Supplementary file 4 [file 13023_2026_4563_MOESM5_ESM.docx]

Supplementary Table 3. Sphingolipid-associated genes selected from GeneCards (Relevance score > 7)

| **No.** | **Gene symbol** | **Description** | **Category** | **UniProt ID** | **GIFtS** | **GeneCards ID** | **Relevance score** | **GeneCards link** |
| --- | --- | --- | --- | --- | --- | --- | --- | --- |
| 1 | PSAP | Prosaposin | Protein Coding | P07602 | 60 | GC10M071816 | 90.781 | https://www.genecards.org/cgi-bin/carddisp.pl?gene=PSAP |
| 2 | DEGS1 | Delta 4-Desaturase, Sphingolipid 1 | Protein Coding | O15121 | 53 | GC01P224175 | 32.028 | https://www.genecards.org/cgi-bin/carddisp.pl?gene=DEGS1 |
| 3 | DEGS2 | Delta 4-Desaturase, Sphingolipid 2 | Protein Coding | Q6QHC5 | 47 | GC14M100143 | 30.214 | https://www.genecards.org/cgi-bin/carddisp.pl?gene=DEGS2 |
| 4 | ORMDL3 | ORMDL Sphingolipid Biosynthesis Regulator 3 | Protein Coding | Q8N138 | 49 | GC17M039921 | 24.209 | https://www.genecards.org/cgi-bin/carddisp.pl?gene=ORMDL3 |
| 5 | SPTLC1 | Serine Palmitoyltransferase Long Chain Base Subunit 1 | Protein Coding | O15269 | 59 | GC09M133501 | 20.957 | https://www.genecards.org/cgi-bin/carddisp.pl?gene=SPTLC1 |
| 6 | ORMDL1 | ORMDL Sphingolipid Biosynthesis Regulator 1 | Protein Coding | Q9P0S3 | 44 | GC02M189764 | 20.390 | https://www.genecards.org/cgi-bin/carddisp.pl?gene=ORMDL1 |
| 7 | SPTLC2 | Serine Palmitoyltransferase Long Chain Base Subunit 2 | Protein Coding | O15270 | 60 | GC14M077505 | 20.349 | https://www.genecards.org/cgi-bin/carddisp.pl?gene=SPTLC2 |
| 8 | ORMDL2 | ORMDL Sphingolipid Biosynthesis Regulator 2 | Protein Coding | Q53FV1 | 42 | GC12P087002 | 19.720 | https://www.genecards.org/cgi-bin/carddisp.pl?gene=ORMDL2 |
| 9 | MFSD2B | MFSD2 Lysolipid Transporter B, Sphingolipid | Protein Coding | A6NFX1 | 41 | GC02P024010 | 19.190 | https://www.genecards.org/cgi-bin/carddisp.pl?gene=MFSD2B |
| 10 | ASAH1 | N-Acylsphingosine Amidohydrolase 1 | Protein Coding | Q13510 | 62 | GC08M018055 | 16.442 | https://www.genecards.org/cgi-bin/carddisp.pl?gene=ASAH1 |
| 11 | SPTLC3 | Serine Palmitoyltransferase Long Chain Base Subunit 3 | Protein Coding | Q9NUV7 | 52 | GC20P013008 | 14.650 | https://www.genecards.org/cgi-bin/carddisp.pl?gene=SPTLC3 |
| 12 | GBA1 | Glucosylceramidase Beta 1 | Protein Coding | P04062 | 62 | GC01M169788 | 13.985 | https://www.genecards.org/cgi-bin/carddisp.pl?gene=GBA1 |
| 13 | SGPP1 | Sphingosine-1-Phosphate Phosphatase 1 | Protein Coding | Q9BX95 | 45 | GC14M063684 | 13.150 | https://www.genecards.org/cgi-bin/carddisp.pl?gene=SGPP1 |
| 14 | ASAH2 | N-Acylsphingosine Amidohydrolase 2 | Protein Coding | Q9NR71 | 55 | GC10M050182 | 13.047 | https://www.genecards.org/cgi-bin/carddisp.pl?gene=ASAH2 |
| 15 | FA2H | Fatty Acid 2-Hydroxylase | Protein Coding | Q7L5A8 | 57 | GC16M074712 | 12.926 | https://www.genecards.org/cgi-bin/carddisp.pl?gene=FA2H |
| 16 | SMPD1 | Sphingomyelin Phosphodiesterase 1 | Protein Coding | P17405 | 61 | GC11P006390 | 12.874 | https://www.genecards.org/cgi-bin/carddisp.pl?gene=SMPD1 |
| 17 | SGPL1 | Sphingosine-1-Phosphate Lyase 1 | Protein Coding | O95470 | 59 | GC10P070815 | 12.864 | https://www.genecards.org/cgi-bin/carddisp.pl?gene=SGPL1 |
| 18 | SPHK1 | Sphingosine Kinase 1 | Protein Coding | Q9NYA1 | 58 | GC17P076376 | 12.701 | https://www.genecards.org/cgi-bin/carddisp.pl?gene=SPHK1 |
| 19 | NPC1 | NPC Intracellular Cholesterol Transporter 1 | Protein Coding | O15118 | 61 | GC18M023506 | 12.386 | https://www.genecards.org/cgi-bin/carddisp.pl?gene=NPC1 |
| 20 | CERS2 | Ceramide Synthase 2 | Protein Coding | Q96G23 | 55 | GC01M150960 | 11.447 | https://www.genecards.org/cgi-bin/carddisp.pl?gene=CERS2 |
| 21 | SPTSSA | Serine Palmitoyltransferase Small Subunit A | Protein Coding | Q969W0 | 46 | GC14M034432 | 11.349 | https://www.genecards.org/cgi-bin/carddisp.pl?gene=SPTSSA |
| 22 | LOC100131127 | Delta 4-Desaturase, Sphingolipid 1 Pseudogene | Pseudogene |  | 4 | GC08P028225 | 11.105 | https://www.genecards.org/cgi-bin/carddisp.pl?gene=LOC100131127 |
| 23 | UGCG | UDP-Glucose Ceramide Glucosyltransferase | Protein Coding | Q16739 | 56 | GC09P111896 | 11.041 | https://www.genecards.org/cgi-bin/carddisp.pl?gene=UGCG |
| 24 | GLA | Galactosidase Alpha | Protein Coding | P06280 | 61 | GC0XM101393 | 10.523 | https://www.genecards.org/cgi-bin/carddisp.pl?gene=GLA |
| 25 | KDSR | 3-Ketodihydrosphingosine Reductase | Protein Coding | Q06136 | 54 | GC18M063327 | 10.496 | https://www.genecards.org/cgi-bin/carddisp.pl?gene=KDSR |
| 26 | SPNS2 | SPNS Lysolipid Transporter 2, Sphingosine-1-Phosphate | Protein Coding | Q8IVW8 | 43 | GC17P004498 | 10.457 | https://www.genecards.org/cgi-bin/carddisp.pl?gene=SPNS2 |
| 27 | GALC | Galactosylceramidase | Protein Coding | P54803 | 57 | GC14M087837 | 9.940 | https://www.genecards.org/cgi-bin/carddisp.pl?gene=GALC |
| 28 | UGT8 | UDP Glycosyltransferase 8 | Protein Coding | Q16880 | 57 | GC04P114598 | 9.900 | https://www.genecards.org/cgi-bin/carddisp.pl?gene=UGT8 |
| 29 | CERS3 | Ceramide Synthase 3 | Protein Coding | Q8IU89 | 52 | GC15M170882 | 9.707 | https://www.genecards.org/cgi-bin/carddisp.pl?gene=CERS3 |
| 30 | ARSA | Arylsulfatase A | Protein Coding | P15289 | 59 | GC22M050622 | 9.483 | https://www.genecards.org/cgi-bin/carddisp.pl?gene=ARSA |
| 31 | ACER3 | Alkaline Ceramidase 3 | Protein Coding | Q9NUN7 | 50 | GC11P076860 | 9.479 | https://www.genecards.org/cgi-bin/carddisp.pl?gene=ACER3 |
| 32 | GM2A | Ganglioside GM2 Activator | Protein Coding | P17900 | 54 | GC05P151212 | 9.385 | https://www.genecards.org/cgi-bin/carddisp.pl?gene=GM2A |
| 33 | CERS1 | Ceramide Synthase 1 | Protein Coding | P27544 | 55 | GC19M018868 | 9.362 | https://www.genecards.org/cgi-bin/carddisp.pl?gene=CERS1 |
| 34 | SMPD2 | Sphingomyelin Phosphodiesterase 2 | Protein Coding | O60906 | 53 | GC06P109440 | 9.140 | https://www.genecards.org/cgi-bin/carddisp.pl?gene=SMPD2 |
| 35 | CERS4 | Ceramide Synthase 4 | Protein Coding | Q9HA82 | 50 | GC19P008206 | 9.103 | https://www.genecards.org/cgi-bin/carddisp.pl?gene=CERS4 |
| 36 | S1PR1 | Sphingosine-1-Phosphate Receptor 1 | Protein Coding | P21453 | 58 | GC01P101236 | 9.101 | https://www.genecards.org/cgi-bin/carddisp.pl?gene=S1PR1 |
| 37 | ACER2 | Alkaline Ceramidase 2 | Protein Coding | Q5QJU3 | 45 | GC09P019409 | 9.066 | https://www.genecards.org/cgi-bin/carddisp.pl?gene=ACER2 |
| 38 | SMPD3 | Sphingomyelin Phosphodiesterase 3 | Protein Coding | Q9NY59 | 53 | GC16M068358 | 8.953 | https://www.genecards.org/cgi-bin/carddisp.pl?gene=SMPD3 |
| 39 | CERS6 | Ceramide Synthase 6 | Protein Coding | Q6ZMG9 | 51 | GC02P168491 | 8.820 | https://www.genecards.org/cgi-bin/carddisp.pl?gene=CERS6 |
| 40 | CERT1 | Ceramide Transporter 1 | Protein Coding | Q9Y5P4 | 52 | GC05M075356 | 8.316 | https://www.genecards.org/cgi-bin/carddisp.pl?gene=CERT1 |
| 41 | NPC2 | NPC Intracellular Cholesterol Transporter 2 | Protein Coding | P61916 | 55 | GC14M074476 | 8.235 | https://www.genecards.org/cgi-bin/carddisp.pl?gene=NPC2 |
| 42 | SGMS1 | Sphingomyelin Synthase 1 | Protein Coding | Q86VZ5 | 50 | GC10M050305 | 8.220 | https://www.genecards.org/cgi-bin/carddisp.pl?gene=SGMS1 |
| 43 | S1PR2 | Sphingosine-1-Phosphate Receptor 2 | Protein Coding | O95136 | 57 | GC19M115645 | 8.120 | https://www.genecards.org/cgi-bin/carddisp.pl?gene=S1PR2 |
| 44 | ELOVL1 | ELOVL Fatty Acid Elongase 1 | Protein Coding | Q9BW60 | 52 | GC01M043363 | 7.784 | https://www.genecards.org/cgi-bin/carddisp.pl?gene=ELOVL1 |
| 45 | GLB1 | Galactosidase Beta 1 | Protein Coding | P16278 | 61 | GC03M032963 | 7.614 | https://www.genecards.org/cgi-bin/carddisp.pl?gene=GLB1 |
| 46 | B4GALT6 | Beta-1,4-Galactosyltransferase 6 | Protein Coding | Q9UBX8 | 50 | GC18M040213 | 7.549 | https://www.genecards.org/cgi-bin/carddisp.pl?gene=B4GALT6 |
| 47 | CERS5 | Ceramide Synthase 5 | Protein Coding | Q8N5B7 | 48 | GC12M050129 | 7.430 | https://www.genecards.org/cgi-bin/carddisp.pl?gene=CERS5 |
| 48 | HEXA | Hexosaminidase Subunit Alpha | Protein Coding | P06865 | 59 | GC15M072340 | 7.423 | https://www.genecards.org/cgi-bin/carddisp.pl?gene=HEXA |
| 49 | SPHK2 | Sphingosine Kinase 2 | Protein Coding | Q9NRA0 | 57 | GC19P048619 | 7.389 | https://www.genecards.org/cgi-bin/carddisp.pl?gene=SPHK2 |
| 50 | ACER1 | Alkaline Ceramidase 1 | Protein Coding | Q8TDN7 | 44 | GC19M006306 | 7.269 | https://www.genecards.org/cgi-bin/carddisp.pl?gene=ACER1 |
| 51 | SGPP2 | Sphingosine-1-Phosphate Phosphatase 2 | Protein Coding | Q8IWX5 | 41 | GC02P222424 | 7.268 | https://www.genecards.org/cgi-bin/carddisp.pl?gene=SGPP2 |
| 52 | CERK | Ceramide Kinase | Protein Coding | Q8TCT0 | 52 | GC22M046684 | 7.223 | https://www.genecards.org/cgi-bin/carddisp.pl?gene=CERK |
