# Supplementary material for: Integrative Multi-omics and machine learning analysis of sphingolipid-associated molecular stratification identifies diagnostic and prognostic signatures in idiopathic pulmonary fibrosis
Source: Orphanet J Rare Dis. 2026 Aug 25;21:384. doi: 10.1186/s13023-026-04563-4 (PMC13733085; doi:10.1186/s13023-026-04563-4)
Supplement: Supplementary file 5 [file 13023_2026_4563_MOESM4_ESM.doc]

Supplemenatry Table 2. PCR primers sequence

|  | Forward Primer | Reverse Primer |
| --- | --- | --- |
| CCDC80 | GACCCCGTTTCACTATGCTGT | GGCGAGCTAGTCTCAACACG |
| siCCDC80 | AATGATGATGATGCTGCTGCT dTdT | AGCAGCAGCATCATCATCATT dTdT |
| α-SMA | GACAATGGCTCTGGGCTCTGTAA | TGTGCTTCGTCACCCACGTA |
| COL1A1 | GAGGGCCAAGACGAAGACATC | CAGATCACGTCATCGCACAAC |
| FN1 | CGGTGGCTGTCAGTCAAAG | AAACCTCGGCTTCCTCCATAA |
| GAPDH | GCACCGTCAAGCTGAGAAC | TGGTGAAGACGCCAGTGGA |
