# Supplementary material for: Integrative Multi-omics and machine learning analysis of sphingolipid-associated molecular stratification identifies diagnostic and prognostic signatures in idiopathic pulmonary fibrosis
Source: Orphanet J Rare Dis. 2026 Aug 25;21:384. doi: 10.1186/s13023-026-04563-4 (PMC13733085; doi:10.1186/s13023-026-04563-4)
Supplement: Supplementary file 6 [file 13023_2026_4563_MOESM6_ESM.doc]

Supplemenatry Table 5. The hub genes identified by hdWGCNA.

| **gene_name** | **module** | **kME** |
| --- | --- | --- |
| DSTN | Fibroblasts-M1 | 0.452319230051467 |
| MYL6 | Fibroblasts-M1 | 0.41887355235724 |
| TPM2 | Fibroblasts-M1 | 0.410150080755624 |
| MYL9 | Fibroblasts-M1 | 0.402417548913722 |
| ACTA2 | Fibroblasts-M1 | 0.402120321119291 |
| TPM1 | Fibroblasts-M1 | 0.401864633264609 |
| ADIRF | Fibroblasts-M1 | 0.38501294010585 |
| TINAGL1 | Fibroblasts-M1 | 0.380333004576165 |
| TAGLN | Fibroblasts-M1 | 0.378618017873701 |
| CRIP2 | Fibroblasts-M1 | 0.375970380693284 |
| CD151 | Fibroblasts-M1 | 0.369960250824965 |
| ATP5B | Fibroblasts-M1 | 0.365607770000208 |
| PPP1R12A | Fibroblasts-M1 | 0.36548432480274 |
| CAV1 | Fibroblasts-M1 | 0.365453216564189 |
| LPP | Fibroblasts-M1 | 0.348802837141641 |
| CALD1 | Fibroblasts-M1 | 0.348267253336453 |
| ACTB | Fibroblasts-M1 | 0.347528128074718 |
| COX7A2 | Fibroblasts-M1 | 0.342485685576195 |
| CALM2 | Fibroblasts-M1 | 0.342136843879779 |
| CAP1 | Fibroblasts-M1 | 0.339175185234791 |
| FLNA | Fibroblasts-M1 | 0.332515638289363 |
| PDLIM7 | Fibroblasts-M1 | 0.330811524009545 |
| ACTN1 | Fibroblasts-M1 | 0.330291849987113 |
| WDR1 | Fibroblasts-M1 | 0.32827628609703 |
| PPP1R14A | Fibroblasts-M1 | 0.32494610148143 |
| PFN1 | Fibroblasts-M2 | 0.518151531253172 |
| GAPDH | Fibroblasts-M2 | 0.506381104501391 |
| ARPC2 | Fibroblasts-M2 | 0.505944818061094 |
| YBX1 | Fibroblasts-M2 | 0.47828473674063 |
| GUK1 | Fibroblasts-M2 | 0.461311735966808 |
| AP2M1 | Fibroblasts-M2 | 0.444632942667678 |
| CFL1 | Fibroblasts-M2 | 0.437716918366368 |
| RAC1 | Fibroblasts-M2 | 0.431392817770208 |
| TCEB2 | Fibroblasts-M2 | 0.425415223861179 |
| NDUFS5 | Fibroblasts-M2 | 0.423995204216493 |
| UQCRQ | Fibroblasts-M2 | 0.423807612885908 |
| PARK7 | Fibroblasts-M2 | 0.423047956964763 |
| RHOA | Fibroblasts-M2 | 0.415567862538686 |
| NDUFB2 | Fibroblasts-M2 | 0.411205421308654 |
| PGAM1 | Fibroblasts-M2 | 0.407776595529206 |
| ALDOA | Fibroblasts-M2 | 0.407558825312425 |
| PSMA7 | Fibroblasts-M2 | 0.407509308896977 |
| UQCR11 | Fibroblasts-M2 | 0.404648404763552 |
| CHCHD2 | Fibroblasts-M2 | 0.402889672553715 |
| NDUFA4 | Fibroblasts-M2 | 0.399080292625282 |
| COX8A | Fibroblasts-M2 | 0.394747125589879 |
| COX5A | Fibroblasts-M2 | 0.392805709768791 |
| PRELID1 | Fibroblasts-M2 | 0.391125670698405 |
| TPI1 | Fibroblasts-M2 | 0.390735684940036 |
| ATP5G3 | Fibroblasts-M2 | 0.390234347743346 |
| COL1A1 | Fibroblasts-M3 | 0.620769599776133 |
| LGALS1 | Fibroblasts-M3 | 0.553719833804282 |
| TIMP1 | Fibroblasts-M3 | 0.548103980127319 |
| SPARC | Fibroblasts-M3 | 0.541330064550362 |
| COL3A1 | Fibroblasts-M3 | 0.537803575164769 |
| KDELR2 | Fibroblasts-M3 | 0.536365699713911 |
| COL1A2 | Fibroblasts-M3 | 0.533970148989403 |
| P4HB | Fibroblasts-M3 | 0.53220201131173 |
| S100A16 | Fibroblasts-M3 | 0.527407342451238 |
| PPIC | Fibroblasts-M3 | 0.526841603838774 |
| SERPINH1 | Fibroblasts-M3 | 0.513293753883912 |
| SH3BGRL3 | Fibroblasts-M3 | 0.513267059427721 |
| ANXA2 | Fibroblasts-M3 | 0.511337538336401 |
| PPIB | Fibroblasts-M3 | 0.502004938196397 |
| S100A11 | Fibroblasts-M3 | 0.488851109827478 |
| TMED3 | Fibroblasts-M3 | 0.472003048528962 |
| PKM | Fibroblasts-M3 | 0.4710249041872 |
| PRDX4 | Fibroblasts-M3 | 0.467660953453826 |
| SERF2 | Fibroblasts-M3 | 0.457803516967251 |
| ARPC1B | Fibroblasts-M3 | 0.45565886253482 |
| CKAP4 | Fibroblasts-M3 | 0.455144666976046 |
| SSR4 | Fibroblasts-M3 | 0.44931642024686 |
| OSTC | Fibroblasts-M3 | 0.447536293100747 |
| LY6E | Fibroblasts-M3 | 0.446929052544287 |
| OST4 | Fibroblasts-M3 | 0.440541943369469 |
| TYROBP | Fibroblasts-M4 | 0.671102880875927 |
| LAPTM5 | Fibroblasts-M4 | 0.656593369054887 |
| MS4A7 | Fibroblasts-M4 | 0.651996554929586 |
| VSIG4 | Fibroblasts-M4 | 0.651271561829756 |
| CD163 | Fibroblasts-M4 | 0.633436894480527 |
| AIF1 | Fibroblasts-M4 | 0.628626580669528 |
| C1QB | Fibroblasts-M4 | 0.616306843063139 |
| LST1 | Fibroblasts-M4 | 0.61351977919266 |
| FCER1G | Fibroblasts-M4 | 0.60579885510892 |
| MARCO | Fibroblasts-M4 | 0.601331012159968 |
| LYZ | Fibroblasts-M4 | 0.595951949039281 |
| SPI1 | Fibroblasts-M4 | 0.595817562352692 |
| MSR1 | Fibroblasts-M4 | 0.589519037232638 |
| HLA-DQB1 | Fibroblasts-M4 | 0.587035442959828 |
| C1QC | Fibroblasts-M4 | 0.582673817357988 |
| TREM2 | Fibroblasts-M4 | 0.578794687716859 |
| C1QA | Fibroblasts-M4 | 0.577099498770454 |
| CD53 | Fibroblasts-M4 | 0.576904277978437 |
| HLA-DMB | Fibroblasts-M4 | 0.57324895233726 |
| HLA-DQA1 | Fibroblasts-M4 | 0.568119281170085 |
| MRC1 | Fibroblasts-M4 | 0.567233095933743 |
| C1orf162 | Fibroblasts-M4 | 0.566924044746346 |
| ITGB2 | Fibroblasts-M4 | 0.560946430325424 |
| PTPN6 | Fibroblasts-M4 | 0.559065179160092 |
| SLC11A1 | Fibroblasts-M4 | 0.555809456407909 |
| RPL13A | Fibroblasts-M5 | 0.761784471386396 |
| RPL13 | Fibroblasts-M5 | 0.757994552195966 |
| RPS18 | Fibroblasts-M5 | 0.750104926675443 |
| EEF1A1 | Fibroblasts-M5 | 0.749417072002781 |
| RPS6 | Fibroblasts-M5 | 0.739787925341286 |
| RPL32 | Fibroblasts-M5 | 0.736970456829494 |
| RPS12 | Fibroblasts-M5 | 0.731049141137924 |
| RPL10 | Fibroblasts-M5 | 0.730673892047588 |
| RPS3A | Fibroblasts-M5 | 0.727093121455897 |
| RPLP1 | Fibroblasts-M5 | 0.723798250237848 |
| RPS4X | Fibroblasts-M5 | 0.722370165200729 |
| RPL18A | Fibroblasts-M5 | 0.717948721288534 |
| RPL7 | Fibroblasts-M5 | 0.717519346134593 |
| RPL27A | Fibroblasts-M5 | 0.708672378722025 |
| RPL12 | Fibroblasts-M5 | 0.702251776516742 |
| RPL26 | Fibroblasts-M5 | 0.701613757009417 |
| RPS14 | Fibroblasts-M5 | 0.701460557294559 |
| RPL3 | Fibroblasts-M5 | 0.700407695766067 |
| RPS24 | Fibroblasts-M5 | 0.695704732015941 |
| RPS15A | Fibroblasts-M5 | 0.690186399787076 |
| RPL34 | Fibroblasts-M5 | 0.687349902023537 |
| RPL10A | Fibroblasts-M5 | 0.687340264286787 |
| RPS27A | Fibroblasts-M5 | 0.685053053000975 |
| RPL15 | Fibroblasts-M5 | 0.681560958805436 |
| RPS23 | Fibroblasts-M5 | 0.681232686146989 |
| JUNB | Fibroblasts-M6 | 0.605557979661882 |
| CEBPB | Fibroblasts-M6 | 0.591280927007047 |
| SOD2 | Fibroblasts-M6 | 0.582313327037227 |
| NAMPT | Fibroblasts-M6 | 0.576956029436322 |
| NFKBIA | Fibroblasts-M6 | 0.575844561990148 |
| PNRC1 | Fibroblasts-M6 | 0.570685189153649 |
| CCNL1 | Fibroblasts-M6 | 0.558340101191726 |
| ZFP36 | Fibroblasts-M6 | 0.543200952585467 |
| PPP1R15A | Fibroblasts-M6 | 0.539415247369756 |
| MYC | Fibroblasts-M6 | 0.534419123469994 |
| MCL1 | Fibroblasts-M6 | 0.526232972228366 |
| C11orf96 | Fibroblasts-M6 | 0.522320454766074 |
| SOCS3 | Fibroblasts-M6 | 0.519666463909618 |
| THBS1 | Fibroblasts-M6 | 0.501908121200904 |
| IRF1 | Fibroblasts-M6 | 0.494154722033722 |
| IER3 | Fibroblasts-M6 | 0.493336403880544 |
| CDKN1A | Fibroblasts-M6 | 0.478940938952248 |
| H3F3B | Fibroblasts-M6 | 0.474996619947602 |
| ICAM1 | Fibroblasts-M6 | 0.465089664545538 |
| SERTAD1 | Fibroblasts-M6 | 0.464391106290796 |
| MAFF | Fibroblasts-M6 | 0.455380491410163 |
| CSRNP1 | Fibroblasts-M6 | 0.454803158610863 |
| IL6 | Fibroblasts-M6 | 0.446498457448995 |
| GEM | Fibroblasts-M6 | 0.445651506264807 |
| CXCL2 | Fibroblasts-M6 | 0.436576509739191 |
| GSN | Fibroblasts-M7 | 0.560847109710023 |
| ALDH1A1 | Fibroblasts-M7 | 0.510643611087004 |
| PLPP3 | Fibroblasts-M7 | 0.496348729315434 |
| ALDH2 | Fibroblasts-M7 | 0.490160614578124 |
| ADD3 | Fibroblasts-M7 | 0.451935620182989 |
| ITM2B | Fibroblasts-M7 | 0.437108712309929 |
| CD55 | Fibroblasts-M7 | 0.430076875903888 |
| ARL6IP5 | Fibroblasts-M7 | 0.429737305493484 |
| LTBP4 | Fibroblasts-M7 | 0.421445441475454 |
| CYBRD1 | Fibroblasts-M7 | 0.41589501469294 |
| CST3 | Fibroblasts-M7 | 0.402290652233315 |
| GPNMB | Fibroblasts-M7 | 0.399352923275723 |
| SPRY1 | Fibroblasts-M7 | 0.392779067651331 |
| ITM2A | Fibroblasts-M7 | 0.391186560536926 |
| EFEMP1 | Fibroblasts-M7 | 0.388350395883718 |
| CCPG1 | Fibroblasts-M7 | 0.388130120031367 |
| SPTBN1 | Fibroblasts-M7 | 0.376344457582023 |
| EID1 | Fibroblasts-M7 | 0.374948203140518 |
| LAPTM4A | Fibroblasts-M7 | 0.374697808417049 |
| TCF4 | Fibroblasts-M7 | 0.363320912976346 |
| MGP | Fibroblasts-M7 | 0.362402906881961 |
| CFH | Fibroblasts-M7 | 0.360382104058446 |
| PLAC9 | Fibroblasts-M7 | 0.355108155278452 |
| IGFBP6 | Fibroblasts-M7 | 0.353640156452195 |
| PAM | Fibroblasts-M7 | 0.352945256146116 |
| SERPINF1 | Fibroblasts-M8 | 0.640506269662088 |
| MMP2 | Fibroblasts-M8 | 0.636887218405549 |
| LUM | Fibroblasts-M8 | 0.633217873702699 |
| VCAN | Fibroblasts-M8 | 0.527362261523158 |
| NBL1 | Fibroblasts-M8 | 0.525815069926586 |
| S100A10 | Fibroblasts-M8 | 0.51865751649932 |
| CCDC80 | Fibroblasts-M8 | 0.50774954350249 |
| S100A13 | Fibroblasts-M8 | 0.490288404317671 |
| PLXDC2 | Fibroblasts-M8 | 0.449498476954677 |
| LGALS3 | Fibroblasts-M8 | 0.432635077280765 |
| PLPP1 | Fibroblasts-M8 | 0.427087583883138 |
| IGFBP4 | Fibroblasts-M8 | 0.426701911035661 |
| TMSB10 | Fibroblasts-M8 | 0.415075970176633 |
| PSAP | Fibroblasts-M8 | 0.399134411463444 |
| PDIA3 | Fibroblasts-M8 | 0.387190698976866 |
| TAGLN2 | Fibroblasts-M8 | 0.38361442189636 |
| ATP6V0E1 | Fibroblasts-M8 | 0.380745385316002 |
| TMEM98 | Fibroblasts-M8 | 0.380320274749398 |
| NPC2 | Fibroblasts-M8 | 0.379275692333687 |
| CFI | Fibroblasts-M8 | 0.375398174496308 |
| LGALS3BP | Fibroblasts-M8 | 0.374955834410478 |
| PLTP | Fibroblasts-M8 | 0.361891009318038 |
| HEXB | Fibroblasts-M8 | 0.354262784217369 |
| ENG | Fibroblasts-M8 | 0.353188376025003 |
| SCARB2 | Fibroblasts-M8 | 0.35072191899245 |
| SEPP1 | Fibroblasts-M9 | 0.65699248648254 |
| FBLN1 | Fibroblasts-M9 | 0.631699231999141 |
| CFD | Fibroblasts-M9 | 0.626596997851105 |
| CYR61 | Fibroblasts-M9 | 0.589167215372245 |
| C7 | Fibroblasts-M9 | 0.586068878996999 |
| DCN | Fibroblasts-M9 | 0.57126011213114 |
| C1S | Fibroblasts-M9 | 0.542555131004867 |
| PMP22 | Fibroblasts-M9 | 0.520370415208258 |
| SAT1 | Fibroblasts-M9 | 0.513787278078002 |
| A2M | Fibroblasts-M9 | 0.506566605021462 |
| PTGDS | Fibroblasts-M9 | 0.506048293363858 |
| DUSP1 | Fibroblasts-M9 | 0.492405362487432 |
| ZFP36L2 | Fibroblasts-M9 | 0.488454262550656 |
| EGR1 | Fibroblasts-M9 | 0.481963509889687 |
| KLF4 | Fibroblasts-M9 | 0.47521846607845 |
| C3 | Fibroblasts-M9 | 0.472544801508454 |
| C1R | Fibroblasts-M9 | 0.472107988893178 |
| JUN | Fibroblasts-M9 | 0.470785905649968 |
| CES1 | Fibroblasts-M9 | 0.465416298763104 |
| EMP1 | Fibroblasts-M9 | 0.455688571524437 |
| MGST1 | Fibroblasts-M9 | 0.443462867992357 |
| NNMT | Fibroblasts-M9 | 0.440766920284809 |
| TNFSF13B | Fibroblasts-M9 | 0.439840324870478 |
| FOSB | Fibroblasts-M9 | 0.432677433434125 |
| DNAJB1 | Fibroblasts-M9 | 0.42795142605117 |
| ZBTB16 | Fibroblasts-M10 | 0.528611423130886 |
| STAT3 | Fibroblasts-M10 | 0.422119449090324 |
| GLUL | Fibroblasts-M10 | 0.417304636425079 |
| BTG2 | Fibroblasts-M10 | 0.410735512546847 |
| TXNIP | Fibroblasts-M10 | 0.409546510346202 |
| FOXO3 | Fibroblasts-M10 | 0.408943159021141 |
| C10orf10 | Fibroblasts-M10 | 0.388872542410587 |
| KLF9 | Fibroblasts-M10 | 0.372009463613079 |
| NFIA | Fibroblasts-M10 | 0.370723566325372 |
| RPS4Y1 | Fibroblasts-M10 | 0.360148535032647 |
| SRSF5 | Fibroblasts-M10 | 0.348526752869017 |
| INTS6 | Fibroblasts-M10 | 0.347187069564277 |
| N4BP2L2 | Fibroblasts-M10 | 0.345707908721938 |
| PNISR | Fibroblasts-M10 | 0.337987798555448 |
| SON | Fibroblasts-M10 | 0.331739919419479 |
| AKAP12 | Fibroblasts-M10 | 0.327457066618586 |
| NR4A1 | Fibroblasts-M10 | 0.322482115572606 |
| ANKRD12 | Fibroblasts-M10 | 0.311438400796894 |
| LEPROT | Fibroblasts-M10 | 0.311347640911606 |
| SRSF7 | Fibroblasts-M10 | 0.307232824270885 |
| CNBP | Fibroblasts-M10 | 0.299154263358052 |
| ARGLU1 | Fibroblasts-M10 | 0.294891992188326 |
| EIF4A2 | Fibroblasts-M10 | 0.281968878426881 |
| DDX24 | Fibroblasts-M10 | 0.279783740207838 |
| CALCOCO2 | Fibroblasts-M10 | 0.260489085511175 |
