# Supplementary material for: Integrative Multi-omics and machine learning analysis of sphingolipid-associated molecular stratification identifies diagnostic and prognostic signatures in idiopathic pulmonary fibrosis
Source: Orphanet J Rare Dis. 2026 Aug 25;21:384. doi: 10.1186/s13023-026-04563-4 (PMC13733085; doi:10.1186/s13023-026-04563-4)
Supplement: Supplementary file 7 [file 13023_2026_4563_MOESM7_ESM.doc]

Supplemenatry Table 6. Intersection gene.

| COL1A1 | MYL9 | MGP | TCEB2 | TMED3 | CAV1 |
| --- | --- | --- | --- | --- | --- |
| SERPINH1 | TPM1 | S100A16 | CYBRD1 | S100A13 | CYR61 |
| IGFBP4 | LST1 | PTGDS | CSRNP1 | SON | C7 |
| C1S | PNRC1 | ZBTB16 | SOD2 | MMP2 | CFI |
| GEM | PRDX4 | CES1 | RPL27A | VCAN | IL6 |
| GSN | PPP1R14A | CFH | AKAP12 | CALCOCO2 | SOCS3 |
| EFEMP1 | CCDC80 | TMEM98 | IER3 | THBS1 | IGFBP6 |
| C3 | LGALS3BP | TINAGL1 | ICAM1 | C10orf10 | EMP1 |
| MSR1 | C1QA | ZFP36 | C1QC | ITM2A | TCF4 |
| PPP1R15A | NFIA | LPP | LUM | TPM2 | CDKN1A |
