# Supplementary material for: Integrative Multi-omics and machine learning analysis of sphingolipid-associated molecular stratification identifies diagnostic and prognostic signatures in idiopathic pulmonary fibrosis
Source: Orphanet J Rare Dis. 2026 Aug 25;21:384. doi: 10.1186/s13023-026-04563-4 (PMC13733085; doi:10.1186/s13023-026-04563-4)
Supplement: Supplementary file 8 [file 13023_2026_4563_MOESM8_ESM.doc]

Supplementary Table 7. Published Prognostic Models

| **Model** | **Author** | **Year** | **DOI** |
| --- | --- | --- | --- |
| Model-1 | Feng et al | 2023 | 10.1186/s12931-023-02582-4 |
| Model-2 | Xia et al | 2021 | 10.1016/j.intimp.2021.107369 |
| Model-3 | Li et al | 2021 | 10.3389/fimmu.2021.629854 |
| Model-4 | Lyu et al | 2022 | 10.3389/fendo.2022.1001563 |
| Model-5 | Zheng et al | 2022 | 10.3389/fgene.2022.865052 |
| Model-6 | Fan et al | 2022 | 10.3389/fimmu.2022.1049361 |
| Model-7 | Qiu et al | 2021 | 10.21037/atm-21-4545 |
| Model-8 | Liao et al | 2024 | 10.1080/1744666X.2024.2315218 |
| Model-9 | Li et al | 2021 | 10.3389/fmed.2021.693959 |
| Model-10 | He et al | 2021 | 10.1186/s12890-021-01799-7 |
| Model-11 | Liu et al | 2022 | 10.3389/fimmu.2022.730186 |
| Model-12 | Guo et al | 2022 | 10.3389/fphar.2022.981604 |
| Model-13 | Zhu et al | 2023 | 10.3389/fimmu.2023.1305025 |
| Model-14 | Zhou et al | 2022 | 10.1186/s12890-022-02269-4 |
| Model-15 | Liu et al | 2021 | 10.1002/cbin.12121 |
| Model-16 | Liu et al | 2025 | 10.2174/0109298673380428250903105810 |
| Model-17 | Wang et al | 2024 | 10.21037/jtd-24-760 |
| Model-18 | Sun et al | 2024 | 10.21037/jtd-2024-2173 |
| Model-19 | Li et al | 2023 | 10.1097/MD.0000000000033330 |
