# Supplementary material for: Integrative Multi-omics and machine learning analysis of sphingolipid-associated molecular stratification identifies diagnostic and prognostic signatures in idiopathic pulmonary fibrosis
Source: Orphanet J Rare Dis. 2026 Aug 25;21:384. doi: 10.1186/s13023-026-04563-4 (PMC13733085; doi:10.1186/s13023-026-04563-4)
Supplement: Supplementary file 9 [file 13023_2026_4563_MOESM9_ESM.doc]

Supplementary Table 4. The DEGs across all clusters.

| IL5 | TCHHL1 | MIA2 | LOC100131129 | FAM71F1 | ALX1 | SLC39A5 | ADD2 | OR1L1 | C3orf45 | LOC157273 | TNNT2 |
| --- | --- | --- | --- | --- | --- | --- | --- | --- | --- | --- | --- |
| LOC149351 | LOC644662 | C1QL1 | XAGE2B | FAM71B | LOC100130348 | LCT | GNG3 | TNP1 | C19orf77 | ODAM | C22orf24 |
| EN1 | LOC283482 | OR5J2 | TCEAL7 | TBX2 | TULP1 | CYCSP52 | FAM196B | PLA2G2F | TBX4 | LOC100128682 | TNN |
| PLEKHN1 | OR2A5 | BARHL2 | CDKN2B | TBX3 | C11orf86 | SLC22A12 | MAGED4B | OR4F21 | OR6X1 | USP2 | LOC440330 |
| DMC1 | ANO7 | LOC100131170 | SDK1 | NRXN3 | ASPN | FLJ45079 | C20orf151 | ITIH3 | TNR | SNAR-B1 | PCDHA13 |
| MYBPC1 | GCM2 | C10orf90 | DGCR10 | SYT12 | OR52E4 | HYALP1 | LOC100133461 | RALYL | CDX2 | MXRA8 | FCN2 |
| MAP1B | SLC24A5 | CHRNB4 | MEPE | OR4F29 | STEAP2 | COL5A3 | OSMR | ZNF774 | LOC389247 | FAM74A3 | OR4M1 |
| HILS1 | LOC100653245 | LOC100126447 | FAM55A | NTSR2 | NRN1 | P2RX6 | CDRT15 | OR2AK2 | ZDHHC22 | WNT2B | GRRP1 |
| LOC255654 | CCDC62 | C15orf55 | LOC100129069 | OR2A12 | GCNT4 | SORCS2 | SELV | LOC440934 | OR6K6 | SP6 | LOC100129935 |
| FGF17 | RNF113B | GPR52 | C1QL2 | MPPED1 | DDX4 | DMP1 | DRGX | DHRS2 | PPFIA4 | FAM182B | MDFI |
| C17orf74 | FLJ33534 | GREB1L | C22orf23 | FAM86B1 | LOC283404 | EFNA5 | SLCO1B1 | MAML3 | LOC100130372 | SPDYE8P | CABP1 |
| CHIC1 | NID2 | LYZL2 | TDGF1 | CHRDL1 | ADAM2 | DNAJB8 | FAM71D | C14orf64 | RLN3 | KIAA0509 | OR2G2 |
| MRGPRD | FAM55D | OR2D3 | GSG1L | LRRTM4 | PDE6C | OR10G3 | VWA5B2 | THEM5 | TTTY11 | LCN8 | SEC62 |
| LOC285943 | LOC90246 | LOC286238 | LDB2 | LOC644242 | WNT8B | RNF180 | VCX2 | LOC284023 | ANKRD33B | PHYHIP | TEX19 |
| ZNF711 | PCDHA4 | CALML6 | LOC100134285 | KLHL22 | L1CAM | SNAR-C3 | OR4E2 | INSC | LOC100132215 | CD300LG | MFSD2B |
| HOOK1 | IL24 | PLA2G12B | OR1F1 | SLC9A10 | FGF3 | OR10A7 | NAG20 | DBX1 | NXPH3 | ROS1 | SLC12A3 |
| RNF17 | LOC100130811 | LOC401180 | TP63 | KCNQ1DN | SCN2A | NKX2-8 | CYP39A1 | C7orf33 | SLC5A5 | C15orf60 | PNMA3 |
| LOC100128787 | TCTE3 | SPACA5 | KAAG1 | NBPF7 | FAT4 | SEC14L2 | FLJ41941 | KIAA0125 | FZD9 | TRIM43 | TXNDC2 |
| C16orf82 | GPM6A | GAGE7 | FMR1NB | FGA | GRK4 | CSRNP3 | GPR149 | C1orf170 | IL21 | LOC100129089 | PHKG1 |
| OR10W1 | BTNL9 | CEMP1 | RCOR2 | C2orf57 | HPD | MYOZ3 | OR4S2 | FAM189A1 | PCK1 | EMID1 | XAGE3 |
| RUNX1T1 | COPG2IT1 | NEUROG1 | LOC283737 | LOC100134368 | PRO1768 | LCE3E | CLEC1B | LOC286083 | CYP3A5 | LOC100131763 | BCL6B |
| KLK14 | CLTC | APLNR | C22orf46 | CNGB3 | MOCS1 | MGC20647 | MEIS1 | OR11H6 | FLJ42392 | CHD5 | BCAM |
| BEND7 | LCN1 | GNAZ | FREM1 | TRIM40 | TPPP2 | CDH4 | OR52W1 | LOC100130458 | MAP3K9 | CRYBB3 | LRRC4C |
| SHROOM1 | DCDC1 | ADCY1 | C2orf84 | FLJ22763 | ZNF483 | GRIK4 | CCNI2 | STS | OR6C6 | CCDC36 | LOC100129917 |
| CECR9 | GAS2L2 | MYH13 | C19orf71 | SLFNL1 | KIAA1984 | LOC729911 | FLJ46552 | MMP21 | OR4D2 | AANAT | TMEM184A |
| ARID3C | SCAMP5 | FLJ42289 | LOC100131242 | SSX2 | HOXD10 | ITIH2 | PHKA1 | SYNGAP1 | PAPPA2 | FAM74A1 | HSPB8 |
| FLJ31356 | CCDC81 | ITGB1BP2 | LOC286190 | LMOD3 | HTR5A | FXYD2 | RGS5 | MYH11 | ARSI | THRSP | OR4F6 |
| LOC440600 | LOC100129072 | CRHBP | TUBAL3 | USH1C | CALCR | SEMA3G | BSND | KCNK3 | ADRA2C | RBMY2EP | PI16 |
| GALNT9 | EPHA1 | OR13G1 | OR9Q2 | FBXO24 | COX4I2 | SPDYA | LOC283728 | OR2Z1 | MYLPF | TDRD5 | LOC149773 |
| LRIT1 | NUP62CL | OR2T12 | HTA | LOC388387 | DEFB103A | PDHA2 | FBLL1 | CCL26 | PIP5KL1 | LOC254057 | TTTY3 |
| SERPINA7 | MAGEB4 | LOC284072 | WNT7B | CACNA2D3 | T | ASB4 | LELP1 | FSIP1 | C9orf169 | LOC100129027 | SLC6A13 |
| PTPN14 | PAX6 | OR4D1 | NEXN | C3orf20 | LOC100128356 | IL12RB2 | PRPF40B | VGLL1 | GATA2 | LOC143666 | FGF8 |
| MMP11 | LOC285000 | CPNE6 | OR52E2 | SLC4A10 | LOC100130453 | DEFA11P | FAM92A3 | FAM163A | SHC3 | UCN3 | SF3B2 |
| SELE | AQP4 | MOV10L1 | SPDYE3 | MAP3K10 | FLJ43390 | EIF4E1B | COL4A1 | CRB2 | GPR61 | LOC729706 | TNFSF11 |
| SP3P | MPPED2 | GLYATL2 | PFN3 | TRIM17 | C9orf79 | CMBL | DRD4 | LOC729970 | DIO3 | OR2T4 | MSLNL |
| TTLL9 | PPBPL2 | UBL4B | NXN | RAET1E | POM121L8P | SOHLH1 | FUT1 | HIST1H2AA | ANO9 | SLC3A1 | SSX5 |
| LOC442459 | HSPA12B | PER4 | NKD1 | C8orf86 | CACNA1D | FGF10 | OR56A5 | TMOD1 | TOE1 | LAMB4 | DKK4 |
| SV2B | LOC402160 | RSPH4A | LOC729626 | PARD3B | NR2E1 | GRXCR2 | LOC100128176 | JSRP1 | CCDC42 | TBX15 | IPO11 |
| HS6ST3 | OR6S1 | LOC653712 | SHANK2 | C17orf64 | MMP23B | SDK2 | LOC100130849 | WDR69 | TNFRSF13B | KIF7 | TBC1D26 |
| CSDC2 | OR2B11 | SNTG1 | ZNF48 | LOC158434 | SLC26A1 | MUC22 | ZNF682 | OR5M3 | MPL | HES1 | LOC100130930 |
| LIPH | BBOX1 | TNNI3K | OR56A1 | TTTY5 | LOC100132859 | RASL10B | CELA3B | SPANXB2 | LOC283483 | GUCY2E | PRSS38 |
| MYH2 | MYL10 | OR4C6 | RNF215 | TMEM196 | OCR1 | GABRG3 | B9D1 | KCNF1 | KLK13 | ACRV1 | LOC730668 |
| SLC22A9 | OR8J1 | CDX4 | OR4K14 | BCRP2 | OR52E6 | MUC19 | DFNB31 | C3P1 | RHOXF1 | SNAR-C4 | OR52J3 |
| OR14A16 | C2CD4B | CES3 | DSG2 | OR5B3 | LOC100131298 | PAQR9 | CLGN | ETV2 | ACTR8 | SLC24A2 | LOC730651 |
| FAM22A | C6orf168 | ANKS1B | ABCA10 | LGALS7 | C2orf62 | PTK6 | PARS2 | BTBD18 | LOC100131650 | RIMS4 | TBX5 |
| CCL25 | GOLGA6L5 | LOC441233 | ZCCHC12 | LOC100129785 | PDE3A | OR11L1 | ADORA1 | SMPX | C11orf36 | GPR26 | ADCK4 |
| RASGRF2 | CNTD2 | SYNPO2L | PRY2 | HSP90AB6P | SPINK4 | LOC285286 | CALCA | FTCD | KBTBD12 | CCDC64B | OR52H1 |
| ZNF221 | EPHA7 | LOC388630 | DHH | OR8B12 | USP12 | ZNF501 | LOC100131232 | KRT16 | ALPL | WNT3 | LOC729040 |
| CDH16 | NKX6-3 | DNAL4 | LOC100132764 | DPP6 | FAM171A1 | SCNN1D | LCE2D | CREB3L3 | PLIN1 | OR51S1 | RHBDL3 |
| PARD6G | PRKCG | ABO | PDZK1IP1 | DKKL1 | SDR42E1 | LOC100131742 | BEND6 | SLC27A6 | C16orf78 | LOC285084 | FGF18 |
| SLC5A10 | DAO | OR2T27 | CXorf61 | AQP7 | DCBLD1 | PAK3 | KIF12 | C12orf34 | CHRND | PLCD4 | QRICH2 |
| LOC339788 | OR5K4 | NR2E3 | TERT | SNAR-G1 | PHLDB3 | OR2M4 | LOC100128668 | CD70 | TRPV4 | RAI2 | ZBTB6 |
| C10orf120 | LOC100131691 | NGEF | OR52A5 | CYP1A1 | C1orf65 | LCE3A | EXOC1 | LOC100134409 | TCEB3C | F9 | C7orf69 |
| ADAMTS1 | CLTCL1 | LOC100128703 | WNT9A | DKFZp547J222 | FLJ46010 | PHF10 | C19orf55 | RPL31P11 | C11orf95 | DIS3 | VCAM1 |
| ABCA4 | NFYA | C17orf82 | LOC100129393 | C3orf36 | FLJ34690 | OR4A47 | CLRN2 | PAK6 | LOC348840 | AMHR2 | OR6C68 |
| SYT2 | TCL6 | MGAT5B | PIWIL2 | FFAR1 | ASAH2 | NACAD | MAGEE2 | ZIC1 | LYPD4 | OR1J2 | MEIS3 |
| KCNU1 | LOC732275 | TCEAL2 | LOC100130009 | PCDH15 | RAVER2 | OR5K2 | COL25A1 | ZBTB48 | LOC147670 | AAA1 | APOF |
| PAK4 | LOC100129973 | ZMYND19 | LOC100129900 | OR2T1 | TFDP3 | GNMT | LOC100128591 | NGFR | SYT13 | SYPL2 | CDKN2AIPNL |
| SYCP3 | IL20 | ZNF687 | UGT1A8 | LOC644919 | C5orf52 | IL17B | TCERG1L | TRIM62 | PDIA2 | LOC730441 | FAM73B |
| LOC284930 | ZBTB46 | EPHA10 | PART1 | USP51 | LRRC10 | DEFB107A | SULT1C3 | MATN2 | OR6N2 | MAGEC3 | PRCC |
| ANGPTL2 | FRMD7 | SPANXN5 | SCG2 | FEV | OR5M9 | OXCT2 | CHGB | LOC100130051 | IPO13 | ADAM3A | FAM83A |
| ERC2 | FERD3L | KLK15 | FLJ46066 | DEFB115 | OR8K1 | GIPC2 | DKFZp451A211 | MPP2 | PHOX2B | CPXCR1 | TEX13A |
| ALDH8A1 | CCDC105 | KIF27 | OR2C3 | EHD2 | OR4C13 | TRIM9 | CCBP2 | ARSE | BMPR1B | TLE6 | ZFP2 |
| ZC3H12D | ACSM4 | CTSL3 | SRD5A2 | APOBEC3D | CCL19 | NPPC | C1orf61 | SH3RF3 | ANGPTL1 | HNF1A | LOC643669 |
| OR5A2 | ZNF835 | B4GALNT4 | KRT36 | MARK4 | CXorf27 | C19orf34 | EFS | OR4L1 | C11orf41 | HIGD1B | GH1 |
| TTC29 | LOC100131107 | RIMBP2 | KCNJ9 | CSHL1 | C15orf33 | LRRIQ4 | ANKRD53 | CCL14 | C6orf81 | CCDC116 | KRT24 |
| GLRA1 | TCF20 | LOC100129297 | LOC100129675 | NLRP5 | SCN1B | TMEFF2 | LRRC6 | PPY2 | SPRR4 | ERBB2 | MYOCD |
| C10orf35 | LOC100131060 | RASSF8 | NEB | CSMD1 | HOXA11 | SLC6A11 | KCNAB3 | C21orf54 | FRMPD3 | LRRC4 | TRPM8 |
| C8orf46 | DPCR1 | C11orf34 | CCDC157 | MPZ | LOC554201 | DLK1 | WISP2 | OR2AE1 | PTPRR | LOC388946 | LOC283665 |
| RFPL1 | NCKIPSD | GATA4 | WBSCR17 | RGS11 | TTC39A | ZNF19 | OR52E8 | ZP2 | CCDC27 | GOLGA6L1 | HRCT1 |
| LRRC55 | GRID1 | GSG1 | PTCH1 | TEKT5 | ONECUT1 | ALLC | ZNF69 | CASP14 | NKAIN4 | OR2D2 | C1orf95 |
| PRAMEF12 | ACCN3 | DPYS | LOC100130778 | RHBG | OR4B1 | POTEA | PRKG1 | SGPP2 | OR4C12 | KCNA7 | LOC100132077 |
| C6orf10 | PYGM | PTHLH | EPM2A | TBR1 | KIF18B | OR13C3 | FLG | SLC26A7 | HECW1 | DMD | ZNF382 |
| ELMO3 | DGKA | FLRT1 | FLJ46120 | KLB | BSX | CSAD | TPRX1 | AMPD1 | BCO2 | PCDHGA5 | LOC283761 |
| CCDC11 | LOC100507421 | FBXL22 | ZBTB7C | CRHR1 | LOC645249 | CYLC2 | WISP3 | ADRA2B | TTLL5 | MSH4 | LOC730139 |
| RPS6KA6 | SNHG4 | EDA | ZCCHC13 | OR10A3 | OR52M1 | NR1I3 | HGC6.3 | HTRA3 | LOC153910 | FNDC1 | HNF4A |
| TRIM15 | ASPHD1 | PTGES | TRIM55 | PTPRN2 | ELOVL3 | KRT25 | SEMA6C | NKX2-6 | ZNF81 | MGAT4C | KNDC1 |
| CETN1 | IRGC | SLITRK2 | LOC440970 | CRTAC1 | LOC100130197 | ABLIM1 | ASCL3 | COL1A1 | LOC100507003 | FOXO4 | PKDREJ |
| GRIA1 | LOC340239 | C15orf34 | PPP1R14D | LRRC43 | OR4P4 | HS3ST6 | TMEM132D | ARHGAP23 | SCTR | C5orf48 | LOC100129515 |
| BTBD19 | MRO | OR2T33 | OR52L1 | C10orf91 | LOC100131023 | RYR2 | LOC727677 | IMP5 | MUC12 | CPLX2 | OR4D9 |
| OR52B6 | CCDC89 | RAB3C | GPRIN1 | MAGEC2 | SIGLEC11 | DKFZp686K1684 | C20orf79 | NXPH4 | LHX1 | CACNA1H | BPY2B |
| GRIA2 | IFNA21 | CCDC70 | RNF151 | FLJ10489 | FBXO44 | OR4C16 | TPH2 | HAB1 | CEACAM6 | KREMEN2 | GDPD2 |
| FLJ13197 | MCCD1 | UGT2B15 | RS1 | CABP2 | TNNT3 | TEX28 | HAUS5 | MAGEA10-MAGEA5 | KLHL10 | PGAM5 | SEMA3F |
| EPHA6 | SLCO1A2 | TCF7L2 | GPR39 | BOC | B3GNT6 | NKX6-1 | PTH | KCNQ4 | PARD3 | HOXC12 | MEP1B |
| TSPAN33 | PLCB3 | OR10G7 | MYL9 | CFC1 | EXO1 | SYNJ2 | SLC5A4 | LOC100131599 | C4orf22 | FLJ45974 | CCL1 |
| CAPN14 | NLRP7 | LACRT | ACSBG1 | KPRP | TTTY9A | DNASE1 | RNASE7 | DSC3 | LOC339568 | CSH2 | GNPNAT1 |
| ROM1 | PLA2G2D | USP29 | BET3L | FNDC4 | KSR1 | SNORA37 | NDST3 | ATCAY | SCARNA4 | MT4 | GAFA3 |
| SIRT5 | MGC34796 | OR9G4 | CCDC135 | GJA10 | OR5AU1 | RGS8 | DBN1 | C10orf112 | SYCE1 | NPAS1 | LOC100132850 |
| RBP5 | PRG2 | SCN7A | CFB | TSKS | OR56A3 | LGALS13 | OR10X1 | MYO3B | FAM48B2 | LOC151475 | LOC339505 |
| C4orf50 | P4HA3 | SLC16A13 | SDCBP2 | FGF5 | NHLH1 | ACSM2A | CADPS | HSPG2 | BAHCC1 | FBLIM1 | NPR1 |
| STC2 | LRRC32 | DCD | LOC100128105 | TRPM6 | C2orf51 | APOA4 | PVRL1 | SLC22A25 | FAM180B | GDEP | TUBA8 |
| MANEAL | NT5DC3 | CSPG5 | POU4F3 | ISL1 | CIB3 | PGBD3 | CD28 | EPHB3 | AP1S1 | AICDA | GPR4 |
| GALP | NT5C1A | LOC283731 | POU1F1 | SLC47A2 | ESYT3 | DAB2IP | LOC285456 | FAM166A | MYPOP | HOXD11 | FAM19A4 |
| SLC4A9 | RARB | PLEKHG7 | LMOD1 | LRFN1 | UCP1 | CACNG8 | NPIP | ADAM30 | KLK12 | BMP4 | LRRC19 |
| LOC730101 | NOX5 | PTPRN | SPANXN4 | CACNA1I | TP53INP2 | MYH15 | IGLV1-44 | LOC400965 | SLC2A7 | KCNA1 | C6orf218 |
| RGS6 | NARG2 | LOC286121 | ENTHD1 | TRABD | LOC100127910 | LOC339685 | OR8I2 | TEKT4 | ABCB1 | KLHL34 | OR9I1 |
| MANSC4 | GKAP1 | CYP2E1 | ADH7 | DNAJC17 | HCG9 | TMOD4 | FLJ33065 | FLJ37035 | FLJ43944 | RUVBL1 | GIP |
| LOC387895 | IGFN1 | AKR1B15 | LY6G6C | GPD1 | HAR1B | C19orf76 | BGN | IQCF6 | SMCR5 | SSC5D | XKR3 |
| OR5L1 | TRPC4 | DSCAM | MGC16142 | SLC1A6 | MGC14436 | GRAP | HOXB7 | MATN4 | DHX9 | PDE6A | CNTNAP4 |
| SLC28A1 | C4orf6 | CAMK2B | NEUROD6 | C6 | NT5DC4 | PRMT8 | GRK7 | YIPF7 | ZNF639 | DMGDH | FAM26E |
| C8orf42 | C3orf79 | LOC100130452 | LOC442497 | TMEM35 | OLAH | LOC100131929 | ARL14 | SSX7 | TSPAN16 | ERN1 | TNFRSF10A |
| LOC284108 | ERMP1 | SLC13A4 | C18orf57 | DEFB109P1 | SPON1 | NAT8B | MYOM3 | NRAP | SCARNA6 | EFNA4 | CCDC73 |
| C7orf45 | ATP8A2 | NT5C1B | KCNS1 | RAPGEFL1 | ADAM20 | HIP1R | KRTAP20-4 | TMEM64 | TTBK1 | CASS4 | GOLGA6L10 |
| MAPK12 | LRRC4B | LOC63930 | C9orf50 | ANKDD1A | CERCAM | FRMPD2 | MAGEA11 | DEFB119 | C16orf86 | LOC100131821 | LOC284570 |
| FLJ42842 | TLL1 | HSD17B2 | CPXM2 | FAM19A3 | C10orf71 | C6orf183 | C10orf111 | FABP6 | LOC391767 | C17orf96 | LRGUK |
| C14orf86 | LOC100132336 | KCNQ5 | IL4 | PRTG | RRP1 | ZNF14 | IFNK | OR5B12 | SLC4A3 | SH2D3A | SUMO1P3 |
| PDGFB | DOC2B | AGAP11 | XKR7 | TFPI2 | HELT | CBLN3 | PRSS27 | ACSL6 | OR10H5 | MAGEA10 | TPTE2 |
| LOC729159 | BCL2L10 | ZNF645 | OR2J3 | LOC100128496 | OR10Z1 | ZSCAN5B | GPR157 | CEND1 | HS6ST2 | VEPH1 | SNCG |
| TMEM53 | FLJ44674 | MAST4 | NTRK3 | LOC100131532 | CELSR3 | ENTPD8 | PGC | KLK5 | SCXA | SLC35B4 | TNFRSF6B |
| H2BFXP | CILP | C15orf59 | IFITM5 | EBI3 | SERPINB9 | LOC100129138 | PRO0471 | PCDHB11 | SYCN | BZRAP1 | CRYGC |
| LOC643711 | HOXA5 | LOC100130587 | GPR139 | CLEC18C | YPEL3 | SERPINA10 | FARP2 | C2orf70 | KCNC3 | LOC100133091 | GPR12 |
| TMC1 | PVALB | C21orf49 | PRTFDC1 | HMGN5 | GIF | ITGB3BP | STL | CUX2 | GYPE | OR51I1 | LOC100130857 |
| GJB1 | FMO9P | DACH1 | LOC643962 | MFSD6L | GABRD | MPP4 | IGF1 | OOSP1 | ELN | ADAMTS6 | OTOP3 |
| LOC100129931 | TAS2R46 | COL13A1 | POMZP3 | FSCN2 | TNFRSF19 | CHRD | LTBP2 | SPANXN2 | XDH | MAGEB6 | DDN |
| CCDC122 | ZNF806 | LOC100129662 | TRPC7 | MEST | C1orf129 | RRP12 | LOC401980 | C1orf141 | LRRC18 | C10orf82 | LOC100130642 |
| LOC100130093 | OR6Q1 | CCDC153 | C6orf138 | LOC151300 | PEX1 | DHRS7C | SHOX2 | ADRA2A | TBC1D29 | SORBS2 | CIDEA |
| RHO | TSPAN7 | CCL27 | PPEF1 | SPTA1 | C8orf12 | EFCAB9 | GGTLC1 | C11orf84 | PRAMEF4 | IGF2BP2 | LOC729121 |
| KLC4 | CBLN4 | LOC645427 | C11orf20 | METTL2B | LOC100130248 | ITGA2 | EFHD1 | LOC644852 | CDH19 | JMJD8 | CPA1 |
| CPNE5 | PALM2 | GALNS | SOX6 | MC1R | SAMD1 | LOC284998 | CARD10 | LMTK3 | F2 | OSR2 | RCVRN |
| ANKRD31 | RSPH10B | FAAH2 | MGC39372 | C14orf81 | JMJD6 | TNNC2 | CCDC33 | FLJ44477 | LOC349196 | ABCA2 | GRIA3 |
| LOC100130148 | LOC100132526 | MECOM | LOC642781 | ATP1A2 | XAGE5 | SFRP1 | GDPD3 | C9orf117 | SYNPO2 | LOC401490 | LOC100133857 |
| TMEM229A | CUL1 | TNFRSF4 | GAS1 | CYP11B1 | HOXC10 | AGSK1 | RTP2 | CAMKV | OR5R1 | TUT1 | MGP |
| TSSK3 | MAMDC2 | AFP | C12orf54 | NPS | NRXN1 | CHRM3 | SEL1L2 | TTTY21 | TNKS1BP1 | UBQLN4 | LOC100132832 |
| NPC1L1 | LEP | DCAKD | B3GNT8 | POM121L2 | POTEB | OR5K1 | SPZ1 | OR2G3 | HOXB8 | OR2K2 | MT1IP |
| SNORA65 | OR6C76 | EMID2 | PPP1R1A | ENPP7 | LOC100129223 | UGT2B4 | SYDE2 | C4orf17 | OC90 | OR10AG1 | FRMPD4 |
| FBXL12 | C7orf61 | GLRA4 | RNF175 | RESP18 | LOC148638 | GUCY2C | PRB4 | LIMS3-LOC440895 | LOC100190939 | CASZ1 | TMC5 |
| ACSM5 | C7orf63 | U2AF1L4 | F2RL2 | TRIM48 | ACTL6B | DKK3 | DSPP | BIK | LOC100130097 | SEMG2 | TP73 |
| LOC553103 | NLGN1 | NFATC2 | IQCC | OR5B2 | NCKAP5 | TUSC5 | RFPL3 | EDNRB | FMN2 | LOC390660 | OR1J1 |
| C12orf52 | FLJ45139 | OR6C1 | FAM47C | BCL2L14 | LRRTM3 | FBP2 | FAM75A3 | MVD | BSN | FABP1 | ACTRT1 |
| OR10K1 | NEUROD2 | PPAPDC1B | HPCAL4 | KCNV2 | IFT57 | CCDC43 | CXCL6 | SEC14L5 | PTN | KRT77 | RCN3 |
| AXIN1 | LOC643355 | PERP | CABP7 | BRSK1 | OR6W1P | WNT8A | ADRA1D | LOC100129399 | PRR16 | ZPBP2 | S100A7 |
| ACMSD | TRAPPC9 | SPAG11B | CALB2 | ASPRV1 | OR9Q1 | COL28A1 | MAPKBP1 | TNIP2 | BHMT | LOC100132167 | OBSCN |
| C5orf50 | BMP10 | IQSEC3 | CYP4A22 | FSTL1 | LOC646976 | CXorf66 | CLCA1 | MEX3A | LOC729032 | OR8B2 | TSNAXIP1 |
| ZNF132 | PRDM14 | CACNG2 | CTXN3 | PCDHAC2 | CDRT7 | LOC100130817 | PTPRF | PENK | LOC729739 | IL9 | C8orf29 |
| KLC3 | C17orf28 | LOC100127940 | HIVEP2 | UCKL1 | VGLL2 | UCK1 | FLJ36848 | PPP1R13B | IL31 | LOC285205 | OR5D14 |
| KRT14 | MTSS1 | LOC440356 | CADM4 | WNT7A | MCC | LOC100132078 | PRRX1 | AVPR2 | CHST2 | H1FNT | LOC100128198 |
| FSCB | ZNF704 | HHATL | KCNC2 | LHX5 | ZP3 | RAB37 | LBH | LOC100133311 | PPP3R2 | SLC25A23 | TSPAN10 |
| AADACL4 | SPRR2B | NBPF3 | SEMA5B | UPB1 | C22orf33 | PHF7 | LOC283914 | C17orf104 | MASP1 | FLJ35946 | WWC1 |
| ITM2C | KRT80 | IL28B | C9orf153 | CCRL1 | CD300LD | RNF25 | XG | ABHD12B | C5orf20 | REM1 | SEC14L3 |
| EPHA2 | FCRL2 | RASGEF1A | FAM43B | GAB4 | DCLK3 | LIMCH1 | CXCL17 | SHC4 | SERPINA12 | C12orf40 | COL6A2 |
| LOC100131067 | HRH2 | NRP2 | C2orf78 | PDGFA | GRPR | LOC729177 | ALS2CR12 | HMGB4 | DTX3 | C1orf172 | CHSY3 |
| IFNA4 | LYPD1 | MXRA5 | OR5M11 | FAM71A | GMEB2 | PAFAH1B3 | MACROD2 | D21S2090E | C20orf20 | LOC283922 | MAL2 |
| GABRA1 | FBLN2 | EIF3IP1 | KIRREL | MICALL1 | FLJ41649 | PRAMEF13 | PCA3 | SGCD | MIB2 | CAMK4 | NPAS3 |
| DYNC2LI1 | EDA2R | AHI1 | CCDC151 | AMPD3 | UNKL | ADSSL1 | PGM5 | MYCL1 | FAM41C | FAM3A | CDH6 |
| WNK2 | DRD3 | ANXA13 | SNAPC1 | LOC641365 | FLJ31183 | ESR1 | ATE1 | WWOX | LOC100130370 | MAMSTR | LOC399708 |
| ZNF556 | NS3BP | LOC100132474 | KIF6 | RDH13 | CENPI | TSPY3 | EXOSC2 | IL1F10 | FATE1 | OLFM1 | C1QTNF9 |
| SPN | ZNF182 | CROT | CBLN2 | RAB33A | LOC440117 | LOC728254 | TMEM200C | SPACA3 | C19orf73 | MMEL1 | FAM123C |
| CCNJL | OR52N2 | IL29 | LOC219690 | LOC100130573 | LOC728739 | LIFR | PLXNB1 | FAM161B | SH2D6 | LOC286059 | TTTY18 |
| BMPER | TSTA3 | PLVAP | AGXT2L1 | CCDC83 | ST6GALNAC5 | GALNTL2 | FAM27L | GFRAL | SYP | IFT74 | P4HTM |
| MYH10 | TYR | FABP9 | KCNH5 | LOC553137 | SPATA24 | ENPP1 | LOC219347 | RNF39 | TFAP2B | OR5D18 | FOXR1 |
| KLHL35 | TRIML1 | OR4C11 | DEFB130 | PAX3 | DISC2 | RBMY3AP | SLC24A3 | NTF3 | SURF2 | PGK2 | PRDM1 |
| ABLIM2 | SGK494 | LOC284191 | PRB1 | TAAR9 | DNAH9 | FADS6 | HDHD3 | FAM25A | CHRM5 | LAPTM4B | OR7G1 |
| FAM70B | SPDEF | C22orf31 | POU5F1 | TNFRSF11B | WFDC5 | SBK1 | KRT23 | LOC100130741 | SFRP2 | CDRT1 | ZNF496 |
| SAA3P | CLDN17 | HIST1H1T | MRAP | GGT6 | SLC35F1 | RHOJ | LOC727982 | CT62 | LOC151171 | LOC150197 | PARG |
| ACOT6 | C14orf45 | LOC100268168 | BANF2 | HRASLS | BTNL2 | LCE3D | SSH3 | C20orf54 | TRH | KIF26A | FLJ16734 |
| SPDYE2 | TAL2 | TRIM31 | OVOS | LOC100129363 | FGF14 | PAGE2B | COL6A4P2 | LOC730961 | DKFZP547J0410 | CDC14B | IL10 |
| ZNF287 | ATP2B2 | FILIP1 | LAMA3 | DSCR8 | TTLL12 | GBX1 | LRTM2 | HSPB3 | DDX49 | MIXL1 | GYPB |
| LRRN4 | NKAIN2 | CDH10 | CTTN | RHOXF2 | RAB9B | PHF21B | LOC100130476 | MAGEB10 | SLC2A1 | PNPLA1 | KCNJ4 |
| PPP1R9A | NUDT13 | XYLT1 | SNCAIP | LOC100652851 | ADCY5 | SNRNP25 | TMEM202 | OR2AT4 | NLRP10 | FAM185A | ZRANB3 |
| ZNF705A | ZNF395 | WDR78 | PRSS37 | BHLHA15 | AKNAD1 | ANKRD1 | ATG4D | AMBP | CORO6 | C4BPB | FAM171A2 |
| LOC286094 | GAST | C12orf24 | MGC45800 | THNSL2 | LOC283174 | CYP21A2 | SMARCA2 | FLJ25328 | C2orf53 | CACNA2D2 | C2orf27B |
| CARD11 | SLC26A10 | PAGE3 | LOC727775 | REG3A | KIAA1661 | CYB5D2 | BPESC1 | ANKRD56 | C15orf58 | CHMP6 | OR5T1 |
| EXOSC5 | ACSBG2 | POLR3G | C9orf152 | DCAF8L1 | NEK5 | C1orf100 | RNF20 | NRG3 | LOC100130890 | LYPD6 | LRP5 |
| PCDHA3 | C1orf226 | PSRC1 | KBTBD10 | LOC100507362 | OR51A7 | ST7 | MRAP2 | RNF152 | SLC25A15 | F3 | DDX50 |
| ABCC6P1 | WIPF3 | LOC100499183 | ZFYVE9 | WFDC12 | SNX21 | GGN | C16orf89 | LOC200261 | THTPA | OSBP2 | C12orf42 |
| NMNAT1 | RECQL4 | SCRN2 | PLA2G2E | CNNM1 | ARSK | KRT42P | EXOC3L2 | DUSP7 | LOC283481 | SYCP2L | C9orf84 |
| DNAH6 | KRTAP19-4 | KIAA1841 | SELP | ERCC3 | LOC100130480 | OR10AD1 | ZNF319 | MBD3L2 | ANKRD24 | HIST3H2BB | GNAT1 |
| ZNF256 | XKR5 | OR10Q1 | EPCAM | C7orf51 | LOC728684 | UPF3A | ACAP1 | LOC283112 | ROBO4 | STK31 | E2F1 |
| PRSS23 | MRE11A | TGM6 | AP3M1 | FAM83H | COX7B2 | MGAT1 | UBTFL1 | MICAL2 | FLJ41130 | PODXL | SIX3 |
| TACC2 | CADM1 | LOC100128437 | GALNTL5 | SLC41A1 | RBPMS2 | FOXA3 | GADL1 | ABCG8 | DUSP27 | LOC100132111 | LTC4S |
| PRLH | DLL3 | LOC283692 | SPATA19 | GPR89B | USP44 | C11orf21 | TM6SF2 | LOC388692 | NT5DC2 | ACTL9 | KLHDC7B |
| CD300LB | C19orf26 | LOC728573 | ELFN2 | OR13C8 | CHRNB1 | AZGP1 | TET3 | FGFBP2 | PALM | GRB14 | APEX1 |
| CNTN4 | OR1M1 | C3orf22 | C2orf74 | PARP3 | P2RX2 | TMEM30B | WFDC10B | WNK3 | MORN4 | FLJ43903 | LOC400958 |
| DHTKD1 | CNTNAP3 | OBSL1 | ADNP2 | KIAA1217 | PLCXD3 | LOC286135 | SCD5 | TMEM71 | OR52B4 | SRRM3 | TMEM215 |
| LOC282980 | COL18A1 | CGREF1 | FLJ32955 | RGS7 | FAM40B | ZNF543 | NTNG2 | DKFZp434J0226 | ZNF519 | SLC37A1 | OR14I1 |
| CHAC2 | OR6C65 | OR13J1 | TMEM145 | ANO4 | C17orf98 | MRPL38 | TXN2 | NPY2R | LOC441177 | ALDH3B1 | PRAMEF11 |
| RALGPS2 | PRDM9 | ZNF225 | CLDN4 | EPAG | IQCH | HOXC8 | KRTAP1-1 | IL22RA2 | GFAP | TIMP4 | SLCO6A1 |
| SEMG1 | NR0B2 | C11orf85 | CD7 | PPARGC1B | KCTD9 | ASB2 | ARHGAP11A | LOC100287225 | PHGR1 | OR4K2 | LUZP4 |
| KLHL38 | ABI3BP | C2orf48 | TLL2 | CGN | TWIST1 | APOD | RASL11B | ST6GAL2 | KCNK18 | HOXD12 | TACSTD2 |
| EPO | C8orf74 | OR8D4 | SFRP5 | SOBP | GABRR2 | AMOTL2 | DYNC1I1 | UTP14A | UGDH | KCNIP2 | PHYHD1 |
| CCDC104 | OR2B3 | CSAG1 | FAM55B | IL26 | ULBP2 | C17orf39 | ANGPT4 | LRIG3 | TTTY22 | CDH1 | SAMD14 |
| LOC648044 | KCNH6 | LOC643327 | KCTD13 | C19orf69 | GJC2 | RNF186 | TAS1R2 | GPR87 | C1QTNF8 | NKAIN3 | DNAL1 |
| CD200R1 | SULT1E1 | TMEM136 | ZNF214 | AIG1 | SATB1 | FSTL3 | FOXA1 | ANXA10 | DUSP14 | LOC286467 | TELO2 |
| KIR2DS3 | AKD1 | TEF | ANXA3 | BMP3 | OR8K5 | CACNB2 | DRD2 | SLC25A18 | RUNDC3A | SMTNL1 | DAZ2 |
| IL12B | NBLA00301 | LOC145837 | CSF3 | NFIX | C14orf180 | MAOA | PTGER1 | SMPDL3B | MARVELD3 | PNLIPRP3 | F13B |
| FNBP1L | CCNT1 | LRRC42 | ATXN3L | OR6F1 | SLC7A5 | POU3F3 | WFDC3 | LOC285878 | GABRE | TMC3 | FAM166B |
| SOX4 | THY1 | GSTTP1 | PRR23A | LOC440104 | FLJ45832 | GRIN2A | FAM101B | PAX8 | UNC93A | OR52N1 | TTC9 |
| LOC100289200 | EFNA3 | LOC644649 | MLF1 | RNASE9 | LOC100132368 | ARGFX | FLJ37638 | LOC440149 | KANK1 | SERPINA4 | LOC100216001 |
| GABRA5 | GPR135 | PDE1C | POLE | LOC100129413 | PRH2 | FNDC8 | KCNA6 | LOC647020 | PSMB11 | MYADML | ZBTB8B |
| LOC285696 | PLEKHA1 | LOC100129311 | GMEB1 | PGBD1 | LOC100131034 | SIDT1 | RARRES2 | CHRNE | PLA2G4F | DMRT3 | B3GAT3 |
| PTPRH | MYOZ1 | HIST1H2BN | ART3 | NODAL | TRIM6 | SPEF1 | ARMC7 | C8orf33 | UBE2E4P | C6orf132 | OR5AN1 |
| NPDC1 | C2orf71 | MMP28 | SIRPD | OR2T34 | FLJ34503 | DEFB113 | PASD1 | BICC1 | F10 | GALR1 | TAF1 |
| CNKSR1 | CCR6 | COQ5 | LIPI | DDI1 | ENO2 | PTPN7 | LIPK | PYDC2 | LOC339260 | C1orf227 | OLFM3 |
| MUC15 | DNAJC4 | SYT8 | ATP6V0A4 | C5orf45 | TP53INP1 | FOXL2 | C1orf81 | ANKLE2 | OR7A5 | CMTM8 | LCA5L |
| MORN1 | ODZ2 | LOC284412 | GPRC5D | LCE4A | NRG2 | ELK3 | SDC4P | HYAL1 | TAF7L | VWC2L | SMOC2 |
| OR4N4 | ERMN | LOC100130701 | TNP2 | FOLH1B | PELI3 | SYCP1 | CCND1 | ZDHHC15 | EPHA4 | LOC100132071 | CCDC67 |
| C6orf15 | MAT1A | GDF10 | XPNPEP3 | HAGHL | ZNF16 | OR5H14 | C19orf53 | MYBPH | SALL4 | LCE2C | LOC100130345 |
| MYH7B | BHMT2 | FHL3 | LOC84856 | CASD1 | SLC30A10 | SSX8 | OR5B21 | PRR5L | PCDHB17 | LPPR2 | TMPRSS11A |
| ACCN2 | PRKCSH | TMEM37 | PNCK | LOC100129196 | RNF5 | TSHZ3 | LAMP3 | ALPP | OLFML2B | PXN | C6orf191 |
| RNF157 | TEX15 | CSMD2 | PTH1R | ECHDC3 | SSTR2 | KCNS2 | ATP2A3 | C9orf43 | OR7E14P | CCDC126 | DLL4 |
| SUPV3L1 | LIM2 | C3orf74 | C12orf33 | NUBP2 | HAS3 | GPR27 | HFE | SGSM1 | TTTY1 | C2CD4D | LINGO1 |
| KRT13 | RGL1 | LOC100652922 | CUZD1 | PMEPA1 | LOC255411 | ARL13A | PADI6 | C19orf75 | SPRY3 | OR52I1 | OR5M10 |
| TRIM49 | TNNI1 | TDRD12 | LOC157627 | TSG1 | GYG2 | SFTA3 | C21orf63 | LOC283435 | C4orf26 | PODNL1 | FKBP10 |
| FLJ44511 | IL17F | CPSF2 | UGT2B11 | SPAG16 | C11orf16 | RBBP5 | C7orf71 | MIPOL1 | OBP2A | LOC100128727 | EIF4E3 |
| MAP7D2 | DEFB112 | BNC1 | DLEU1 | FLJ21408 | PTP4A3 | KCNK1 | AGPAT1 | KCNN1 | MARVELD2 | ANKRD30A | KDM6B |
| SMYD5 | RNU105C | METTL11B | FOLH1 | DUSP26 | REM2 | LOC100128529 | THAP8 | PAGE4 | OR10G8 | STMN2 | DGKK |
| KLF5 | CYP24A1 | TSEN15 | SKIL | LOC642924 | SRPX2 | KRTAP5-5 | TULP3 | SELL | ZFYVE19 | MYCNOS | MRGPRX2 |
| RIMS2 | PTF1A | DHX57 | SOX14 | CLDN18 | PTCRA | GNRHR2 | SYT1 | LOC151760 | MUC21 | LAIR2 | PRR23C |
| LOC388588 | ACPT | FFAR2 | APOA5 | TPCN1 | UPK2 | C17orf72 | LRRC66 | IRF3 | ZNF780B | MUSK | SDCCAG8 |
| PLCE1 | GRIP1 | C17orf51 | HINFP | TRIM50 | ARHGAP10 | UROC1 | FOXA2 | RHOF | LOC284276 | LOC100129884 | DBC1 |
| TCP11 | TRAPPC2P1 | RHPN2 | WDR20 | LOC100132987 | KIF17 | PRR18 | ISL2 | SPRR2A | DNAH10 | FGL2 | KANK2 |
| TBCCD1 | C6orf201 | ZNF454 | ANAPC7 | MFSD2A | OR13F1 | ZNF786 | MDM1 | C10orf131 | WBSCR28 | PBX2 | FCRL3 |
| NRM | SIPA1L3 | MAGEB18 | KNG1 | COL16A1 | RHBDD1 | F11 | MYPN | LOC401463 | FAM158A | PIGR | NPY1R |
| NKX2-1 | FAM171B | UBL4A | CHST15 | TGFBR3 | GTF2H1 | PDYN | CCKBR | SEMA6B | GPR68 | CCDC63 | LAMA2 |
| CIT | C4orf36 | FAM71E2 | TTC1 | PVRL4 | FAM66A | MEP1A | UNC119 | GPR101 | OR8U8 | SMCR8 | ACYP2 |
| ASPDH | OR51Q1 | NEK10 | CNTNAP2 | LOC100130800 | KCND1 | KRTAP27-1 | ABCC8 | INGX | LOC145783 | GP9 | ZNF620 |
| LOC338963 | FAM186B | CD300C | C9orf72 | ISX | HCRP1 | WT1 | GPR18 | TSSK2 | NUDT16L1 | GNB5 | TNKS |
| LYPD6B | RGN | LOC100130354 | FBXO2 | OR1G1 | PRR15 | LOC641467 | B3GALT2 | LOC255025 | OR2T6 | LAD1 | VRK2 |
| SYCE1L | DLX1 | MIP | MTNR1B | CREB5 | JAKMIP2 | ABTB2 | LRTOMT | C8orf45 | LOC729451 | RAX | CYP4F12 |
| PRDM16 | CSN3 | LBP | C4orf45 | FTSJ2 | IQCF3 | IRGM | MYO18B | LRG1 | ZBTB12 | C10orf122 | TC2N |
| CDCA4 | HHIPL2 | SLC13A3 | SLC7A14 | DKFZp434L192 | GTF2IRD2 | CYP2A7 | ASCL1 | BASP1 | GTF2A1 | LYVE1 | C17orf103 |
| TTLL13 | DHRS11 | ABCC1 | OTC | PPP2R1B | OR2C1 | ROBO2 | NLRP13 | PNLIPRP2 | TMEM200B | TBX6 | BRIP1 |
| PSG11 | BTC | EPS8L2 | TRNP1 | FOSL2 | PCDHA6 | ZNF45 | ASB14 | GBX2 | HGD | SLC6A20 | KIAA1644 |
| SLC17A4 | CHST10 | LILRA1 | DSP | C13orf30 | TMPPE | TUBA3C | ADAMTS15 | S100G | SLC10A1 | HLX | MYBBP1A |
| TECRL | SCN2B | SCAPER | TRMT2A | ITGA11 | G6PD | MAGI1 | LOC158960 | SVOP | MSGN1 | HIST1H2BA | LOC284240 |
| PARD6A | C1orf70 | CAMTA2 | FAM108C1 | KCTD8 | EXOSC6 | OMP | IGFL1 | LOC100131654 | BCORL1 | MRPL12 | PRO1596 |
| EFNA1 | STON2 | SEPT4 | LOC100272216 | HEATR7B2 | OPN3 | DNAJB3 | HSD3B1 | FAM69C | TNRC6B | PRM1 | ZNF804B |
| PCDHGC3 | RELN | LOC729603 | TNNI2 | VAMP1 | FZD3 | H1F0 | AP4M1 | BSPH1 | LOC100129198 | TGM4 | LOC283486 |
| SPRR2G | DEFB114 | C10orf99 | GABRR3 | FCAMR | LOC285547 | SLC23A1 | TNNC1 | FZD10 | PROKR1 | TTYH2 | S100A12 |
| ARL10 | PPYR1 | GALNT5 | HUNK | DAND5 | SDR9C7 | LOC644100 | EREG | FAM41AY1 | OTOP2 | LOC100132724 | C1orf101 |
| LRRC16B | OR51M1 | C19orf23 | TCEB2 | ASB6 | TRMT2B | TCTN2 | SAMD3 | FABP7 | STAP2 | CXorf49B | LDLRAD1 |
| B4GALNT3 | TMEM114 | SLC10A2 | GMCL1 | PPP1R2P9 | TGM1 | CDC42BPG | SCN3A | MARCKSL1 | OR4X1 | OR10V1 | GRAMD3 |
| ASTL | TNFSF14 | RP1L1 | HOXD4 | OR2H2 | CCDC147 | TMEM154 | NFU1 | DOK6 | CCDC61 | C1orf185 | IL22RA1 |
| LOC100289509 | LOC100652791 | GPR172B | FGFR2 | PWRN2 | HSD17B3 | LIMD1 | LOC285370 | ETAA1 | ZC3H18 | ESM1 | KRTAP20-1 |
| SNORA58 | LIMS3L | OR51V1 | LOC649133 | HOXC9 | ALDOAP2 | LOC343052 | LOC100128401 | SGK2 | RPS6KL1 | MAGEA4 | RAX2 |
| CXorf1 | CRYBA2 | DCDC2B | CENPH | SERPINI1 | CNTROB | SLC12A8 | LRRC30 | APOBEC4 | LOC644248 | TSPYL2 | GPATCH3 |
| LOC100129520 | HOXA9 | LOC283663 | WNT10A | ST8SIA4 | TTLL2 | C8orf39 | ACTR5 | OR5B17 | OR6T1 | PIWIL1 | LOC284244 |
| HCG22 | ADD1 | HAPLN1 | LOC283089 | TTTY8 | ZNF804A | DYNC1LI1 | EGLN3 | PANX2 | GATA5 | LRRC56 | ARHGEF9 |
| C16orf11 | TNMD | ZMYND12 | GLIPR1L1 | OR51E1 | SNORD9 | SMPDL3A | ADAMTS16 | PNLIPRP1 | INS-IGF2 | OR6B2 | CITED1 |
| PRDM8 | TNFRSF13C | XGPY2 | ZNF560 | LOC100131840 | ELSPBP1 | C1orf116 | TSPYL4 | CA11 | C6orf94 | OXT | DLG3 |
| C6orf154 | ECM2 | LOC100131000 | FAM122C | OR7C1 | APLP1 | TAF3 | PRODH2 | GNRH2 | CPB2 | FHIT | OCIAD2 |
| HPX | ZC3H7B | SMOC1 | C19orf46 | SLC25A21 | LOC100134256 | VTCN1 | RAB3D | TEAD2 | HOXC13 | DGKE | OR6C3 |
| ZNF425 | FLJ46906 | SLC39A14 | MED11 | PLIN5 | IL1RAPL1 | SLC30A3 | HEPACAM2 | PHOSPHO2-KLHL23 | HNF4G | ZNF805 | C6orf203 |
| MEF2C | AOX1 | FLJ16341 | SEPT1 | C1orf115 | RSF1 | CTCFL | LOC340094 | MUC17 | DIRC3 | LOC649201 | TSGA13 |
| IFNA6 | CDH3 | FLJ41278 | AGR3 | SLC7A11 | LOC339539 | MYO1B | MUC7 | TRIM24 | ZNF419 | KBTBD8 | FOXS1 |
| ZNF609 | S100A2 | CCDC114 | MYBPC2 | C1orf53 | SPESP1 | KIRREL3 | LOC728316 | CLDN20 | GREM2 | IL23A | FOXF2 |
| HSP90AA5P | MMP13 | C8orf28 | F7 | SIM2 | MUCL1 | MMRN1 | TMEM161A | CD19 | SPNS3 | LOC338667 | AIM1L |
| KEL | CWH43 | KIF19 | NTNG1 | SEZ6 | ZNF761 | NRG1 | ZNF433 | DLX6 | CACNG7 | GHRL | EBF1 |
| MAPK10 | LGALS14 | FAM123A | PCDHGA3 | C16orf93 | CCT6B | ZNF511 | LOC100129186 | LHX8 | CDH11 | FANCG | CMYA5 |
| LOC100505719 | XKRY2 | KCNB1 | C5orf22 | PHF21A | SCN11A | FLT3 | LOC388780 | RAP1GAP | C9orf11 | C19orf21 | OTUB2 |
| ZNF660 | LOC641518 | PPP1R3A | PUS1 | IGSF10 | LOC730102 | SIRPA | CALML5 | LOC51145 | TUBGCP5 | ALOX12P2 | C1orf111 |
| LOC157931 | C5orf34 | ERBB3 | ATP2B1 | MAK | VAX1 | CDH26 | BOLA1 | OSTalpha | ST6GAL1 | PCDH10 | C1orf49 |
| SLC34A2 | SNTG2 | SPATA4 | KIAA1324 | DKFZP564C152 | HGF | HAPLN3 | OPRM1 | LHFPL1 | LOC100128075 | BTG4 | CPNE9 |
| ATP6V1G2 | KIAA2018 | CINP | NTHL1 | C4orf34 | OR4K5 | BIRC8 | HIST2H2BA | TSPAN11 | TREML3 | TAL1 | CA6 |
| LPO | FBXO15 | LPAR4 | LOC100134937 | TMEM132C | LOC389332 | FLJ38379 | SORCS1 | MEIG1 | UNC13B | GCNT6 | RIPK4 |
| GPR32 | CCL8 | TKTL2 | C9orf96 | ASNS | DLG2 | IGKV1D-13 | EDN3 | CHFR | NUAK2 | ELAVL2 | HIST2H2BE |
| TFAP2C | BRWD3 | INPP5B | SLC22A11 | GOLGB1 | UBXN7 | DERL1 | KIAA1919 | FAM150A | LOC286370 | SART3 | LOC100129427 |
| THAP11 | FHL5 | IRF4 | OR2Y1 | SVOPL | FOXB2 | ANKRD20A1 | DUX3 | ABCC12 | MAP1LC3A | ADPRH | MAG |
| FLJ26850 | PAF1 | C10orf47 | ERF | LOC100129119 | LOC100509378 | KLHL5 | MRPL28 | PCDH18 | DCAF12L1 | SLC39A6 | LOC100130880 |
| LOC100128788 | CELA2A | LOC100131910 | POU2AF1 | IGFALS | PRKAR2B | GSTA3 | APOBEC3A | GJD2 | FCAR | MGC12982 | TTC23L |
| TRAF3 | CC2D1A | CDH17 | CD27 | ACTR3C | TF | OR7E24 | IGFL3 | CIB2 | C1QL3 | ADAMTS12 | TAS1R1 |
| TUBB3 | IKZF3 | MERTK | MYLK2 | SPSB1 | MFHAS1 | CHRNA2 | ALAD | C3orf52 | TCF21 | C12orf63 | GPR19 |
| NCR1 | SLC10A6 | KRTDAP | DNAI2 | LHX4 | FLJ13224 | C18orf62 | LOC729424 | PIGN | LOC285033 | EPGN | MED29 |
| LOC347411 | C6orf141 | C9orf86 | ATP6V1G3 | EFHC2 | C8orf77 | COMP | B4GALNT1 | ITPR3 | TESC | EPHX4 | HEATR4 |
| SYF2 | FAM9C | OR8B4 | ZFYVE28 | CHD3 | RIPPLY2 | ETFDH | ITPRIPL1 | C7orf66 | LRRC1 | AEN | GAS7 |
| ZNF35 | PPAN-P2RY11 | AK5 | WDR93 | TMEM63C | ZER1 | GSTTP2 | DEFB1 | NLGN2 | OR52K1 | FDXACB1 | ADAM12 |
| LOC100287704 | CLEC4E | ZNF224 | HPSE | RERE | C10orf128 | C14orf91 | LYZL6 | GDF6 | SCARNA2 | NKIRAS2 | ZNF787 |
| ANKK1 | DDAH2 | TEDDM1 | DEFB123 | DNMT3A | TRIM46 | FOXQ1 | THSD4 | UPK3B | PCCA | PTH2 | DGCR5 |
| LOC100128019 | RCSD1 | KIAA0664 | TTC25 | ZNF530 | KIAA1274 | TMEM86A | FMO4 | HDAC4 | INSL6 | C2orf42 | LOC338620 |
| TNFSF9 | ADAMTS20 | LOC100507634 | PHF14 | DHRS7B | BANK1 | LOC100128905 | TPH1 | TMED3 | LOC619207 | TCEA3 | SERPINB10 |
| MED31 | ATP2C2 | NR0B1 | LMF1 | LOC440173 | AFM | OR4F4 | C17orf81 | LOC144742 | KIAA1908 | C20orf166 | SLITRK1 |
| NR5A1 | MYO1D | LEFTY2 | LOC285095 | C2orf83 | HSD17B8 | CLCA3P | LOC100506459 | LRFN2 | GALNTL4 | LOC340512 | ATP2A1 |
| FLJ46361 | MCF2L | EFCAB4A | SCGB3A2 | FBXW8 | GLCCI1 | TMEM104 | STX18 | AASS | ROBO3 | EEFSEC | NUCB1 |
| RP1-177G6.2 | NAF1 | C1orf187 | MGC24103 | C11orf67 | KCNN3 | DZIP3 | KRTAP19-5 | MARCKS | CCDC142 | CAPZA3 | WDR52 |
| DEFB134 | VPS13D | SLC25A27 | DEFB104B | SLC9A7P1 | LOC344967 | INTS8 | TGIF2 | BIRC3 | FAM198B | RXFP2 | KY |
| UCN2 | KIAA1244 | LOC647983 | LOC651337 | RCE1 | HEATR6 | POF1B | GSDMD | SLC22A18AS | KCNK17 | LRRC36 | PRKACG |
| KLK2 | LOC678655 | LOC100131354 | TMEM225 | LENEP | GTSF1L | RGPD1 | SLC4A7 | WFDC8 | SFTPB | DMPK | TJP1 |
| TMPRSS5 | C13orf16 | HMHB1 | DLL1 | VSTM2A | B3GNTL1 | GRM4 | PCDH9 | FBXL13 | GPR148 | NEIL3 | LOC285593 |
| PSG1 | APBA3 | FLNC | PRDM12 | C7orf52 | TRPV6 | ALS2CR11 | TSPAN5 | FGF12 | MARCH11 | LOC282997 | LOC728342 |
| CAV1 | CACNB4 | TMEM57 | NRCAM | LOC100130342 | PMF1 | SERPINH1 | MTUS1 | ATP10B | ARL4C | PIH1D2 | ITCH |
| LOC388882 | CMTM2 | LOC646168 | FSHB | MPV17L | ITGB6 | SYT10 | RBPJL | UBE2U | LOC728178 | GEMIN8 | C1orf195 |
| ZNF629 | LMNB2 | SMOX | DEFB136 | C9orf41 | C3orf71 | BMP15 | CYB561 | TSSK4 | SIK3 | TAS2R14 | MGC39545 |
| CHCHD5 | LOC387646 | WDR64 | STAC3 | TTTY6 | PDCL2 | PFDN6 | HRASLS2 | C1QL4 | SLC8A3 | PODN | LOC400464 |
| PON3 | OXCT1 | TMEM174 | OR2V2 | WWTR1 | TADA1 | CT45A1 | LOC100131043 | TPM1 | NUDT21 | DGKG | CYSLTR1 |
| BBS10 | SMCP | RGS20 | RFC3 | PLAC1L | SCARF1 | SPNS2 | DUSP16 | LOC100133957 | IFFO2 | USP30 | ERC1 |
| SCLT1 | HMGCLL1 | C10orf11 | RHOBTB3 | LOC1720 | PPAPDC2 | C17orf67 | FLT4 | GMPR | GDF3 | PIGU | TES |
| NLE1 | LOC221814 | FAM83D | IFT81 | PSG8 | LOC253573 | FGF4 | KLK7 | PXT1 | ACPL2 | PAR4 | MESP1 |
| REXO4 | LOC285375 | SLC38A1 | KLRC4 | TMEM45A | MAGEC1 | EXOSC9 | TGM5 | KRTCAP3 | KLF12 | C15orf29 | PNLDC1 |
| TAPT1 | PRKAR1B | TTYH1 | CDCA3 | AXIN2 | IL6R | AOX2P | AOAH | PROM2 | PIGQ | CTTNBP2 | C9orf3 |
| GPSM2 | SESTD1 | CLCN2 | STMN3 | LOC285796 | IRGQ | UBE3B | HAR1A | C14orf119 | RNF41 | CYP7B1 | EPHA5 |
| RABIF | KRT39 | LRRC2 | SAC3D1 | SARM1 | RGS12 | RBM38 | CNGA4 | LOC285103 | SPANXA1 | PPP4R4 | WNT9B |
| TLE1 | ARHGAP28 | LOC100128792 | SLC12A5 | AGTR2 | LOC100128988 | CD93 | STEAP1 | LOC100129620 | LHFP | LOC283861 | ADAMTS2 |
| NUBPL | GNRH1 | LOC645949 | LAMC2 | LOC441025 | MYOT | GPC6 | LOC339926 | SLC15A1 | SCNN1G | MMP12 | SNRPD1 |
| ALKBH2 | HABP2 | KDM5B | NPTX2 | C12orf39 | SLC22A4 | ADAD2 | CSPP1 | CPN1 | UNC13D | FAM19A1 | ZNF275 |
| TMEM194B | C9orf9 | LMTK2 | ENAH | LOC439950 | STRBP | FAM104B | MAGEB1 | NLRC3 | LOC100128262 | CD1E | LOC100127946 |
| PPM1J | GUCA1C | AK1 | LOC100128482 | LOC642335 | GABRB2 | NID1 | PCDHGB1 | SNORA50 | LIPT1 | ATP4B | LIPJ |
| C9orf171 | OR6B1 | AADACL3 | MTHFR | CA4 | LOC643201 | ITGB3 | SLC2A3 | WNT5B | SPEF2 | SLIT1 | TIGD7 |
| HIF1A | PCDHA8 | FLJ40453 | FAM138D | CD38 | ZNF343 | IFNA14 | LOC100653515 | ARL11 | PKP3 | LOC643401 | DYNLRB1 |
| KLRG1 | SPANXN3 | RAB34 | LOC100506748 | CDK6 | LOXHD1 | PKP4 | PLS3 | INE1 | AGPAT9 | OR5H2 | NLRP4 |
| BDNF | ANAPC2 | SIGLEC6 | DEC1 | GJD3 | LMOD2 | RARG | S100A16 | PHC1 | KIF3A | CYLC1 | KIAA0090 |
| DEFA8P | CTAG1A | CNN1 | PRG3 | MAP6 | CA9 | TMEM171 | BCL9L | UNC45A | LOC285281 | RBM19 | CRB3 |
| REG3G | CTRC | LOC100270710 | SRPK2 | IGSF21 | IGSF1 | DCBLD2 | NUDT9P1 | OR51L1 | NUDT17 | SMC1B | TEAD1 |
| ID1 | TNFSF18 | DPPA3 | TNFRSF10C | CEACAM16 | KRTAP6-1 | FLJ25758 | ANKRD35 | LIG1 | TMEM132A | TGFB2 | WFIKKN2 |
| DBF4B | STK32A | GPR150 | RASSF2 | ABCG4 | PELI2 | TMEM63B | NOBOX | KIF4A | SPIC | FABP12 | CSNK1G1 |
| LRRC61 | TPD52L3 | GPR123 | GSC | SPIB | S1PR3 | PCYT2 | CRYM | MID1 | LOC100131510 | PREX2 | CD180 |
| TP53TG5 | NLRP3 | TMEM106A | KCNJ10 | ZSCAN23 | LZTS1 | FBXL14 | IFT122 | C1QTNF6 | TMEM30C | GPR20 | KCNE1L |
| SCUBE3 | ZC3HAV1L | VPS33B | RASGRF1 | C11orf65 | IL12RB1 | RMND1 | ST3GAL4 | TREML2 | FGF21 | EIF5B | RHOH |
| ZNF599 | C9orf66 | GNGT1 | MMP24 | PAX2 | SCRT2 | LOC100189589 | CCDC152 | ITSN1 | FAM182A | PGP | OR7G2 |
| CCDC40 | CNTN1 | C8G | LOC100131608 | NEDD4L | ADH6 | SLC39A7 | ZNF417 | OR1S2 | TLE3 | PRSS1 | GOLGA6L6 |
| ARHGEF4 | ABCA6 | LOC401497 | LMNB1 | CPZ | TWISTNB | ZSCAN22 | ZNF518B | CYP26B1 | C4orf37 | MFSD4 | LOC647107 |
| NLRP6 | NPR3 | PRAMEL | NTSR1 | SPINT4 | LOC389676 | TEPP | SYT5 | PRPF3 | CD160 | CCDC57 | EEPD1 |
| WFDC1 | C7orf41 | FAM174B | LOC100128699 | USP17L2 | TXNDC15 | OR52D1 | LOC284561 | EDAR | HIVEP3 | C12orf68 | ST6GALNAC1 |
| SMEK3P | CASC2 | PRB3 | SERPIND1 | LOC100128573 | NPHS1 | NHLRC1 | FGF7 | CBX8 | LOC100131719 | PDE6B | S100A14 |
| MOGAT1 | METTL9 | C3orf25 | MN1 | LOC100130705 | LOC642852 | JHDM1D | FAT1 | NEK2 | RARRES3 | AURKB | RASEF |
| C17orf69 | WDR4 | IDI2 | PPP3R1 | TMEM217 | ST8SIA1 | SUFU | BDH1 | CDKL4 | OVCH2 | C15orf27 | ARHGDIG |
| MRS2P2 | PCDHGB3 | NUMBL | ZNF764 | DNHD1 | GUCA1B | GNG4 | CA3 | LENG1 | AGER | SNPH | HOXB1 |
| CEP70 | GPR110 | CYB561D1 | CYLD | LIF | LOC100131381 | METTL11A | CYP2F1 | OR4A5 | PINX1 | POM121L9P | CHKA |
| CYP46A1 | SH3TC1 | EFR3B | ZNF608 | EPHB4 | PRPF19 | TCFL5 | DEFB105B | CEACAM1 | YY2 | ABCF2 | LOC100133308 |
| AARSD1 | PLCH1 | ANKRD34B | EOMES | LYPD3 | LOC100133286 | OR1L8 | C9orf131 | C15orf40 | STEAP4 | IL22 | TSGA10IP |
| STX1A | SLC27A2 | N4BP3 | SRD5A1 | DEFB118 | ATOH8 | CD300A | DNAJB7 | CLCA4 | TMEM26 | MUM1L1 | RLBP1 |
| PCDHA10 | MTBP | PHC3 | ARSA | SLC35D2 | PLOD2 | ARNT2 | CACNG1 | FAM129C | GIN1 | KIAA0087 | SPEM1 |
| DLX5 | LOC100132234 | AGK | PMPCB | FAM92B | COBLL1 | FAM18B2-CDRT4 | VWA3A | COL4A3 | NR2F1 | TACC3 | IHH |
| VENTXP1 | SLA2 | RPL13P5 | RPE65 | ESRRB | ZBP1 | HPVC1 | AQP12A | ACVR1B | C6orf57 | FOXP3 | SHOX |
| OXER1 | HBE1 | LRFN3 | DDX41 | MYH16 | RAB14 | ZNF366 | SPOCK3 | MAMLD1 | FLJ40536 | FANK1 | ANGPTL7 |
| ZNF582 | RNASE10 | GHRH | LOC340074 | OSTN | LOC653581 | C10orf95 | ANKRD29 | ZNF565 | C18orf34 | LRRC57 | ZCCHC7 |
| C20orf152 | FGF22 | VPS8 | INO80C | GABRQ | C8orf59 | CLDN8 | PLD6 | ACAD8 | HOMEZ | LOC283674 | PDCD11 |
| VGLL3 | ACTC1 | DNAH3 | STK33 | MAFK | ZNF385B | C14orf23 | BMF | AKR1CL1 | ROGDI | DOT1L | SNORA2B |
| NTRK1 | SCRT1 | A2LD1 | SLC38A11 | LOC340073 | ITGA4 | TRANK1 | TAC3 | FZD5 | TRPA1 | VPS37D | CXCL14 |
| FBF1 | GGT1 | KLK1 | PRR11 | YPEL1 | GABRP | C3orf37 | NCOA3 | PMAIP1 | WNT2 | FIGN | DDX19B |
| BNIP3 | LOC727915 | C19orf22 | NOX3 | CACNB3 | POU3F2 | OR5P3 | GALNT8 | A2ML1 | DLX3 | USP26 | TSHZ1 |
| CYMP | SLC39A4 | ZNF676 | LOC339975 | GAFA1 | BCL11A | OR4K15 | SP8 | OR13C2 | LOC643542 | TMPRSS6 | SYTL5 |
| FBN3 | MORC1 | CKS1B | GH2 | STC1 | OR14C36 | GPR137 | SLC9A2 | LOC100129042 | MYH1 | AEBP1 | NCAPG |
| C5orf28 | HTRA1 | LOC100128252 | HOXA7 | RRAD | KIF21B | SLC40A1 | SLC26A8 | RALA | LIN52 | HOXD8 | C4orf44 |
| FBXL16 | ZNF138 | LRRC17 | EMCN | C19orf51 | LOC613266 | SMYD1 | LOC253044 | UBASH3A | EPDR1 | TOMM40 | ZC3H10 |
| RYBP | WDR87 | LOC100653275 | KRT222 | SLC34A3 | LTBP1 | VDR | STK36 | LOC100128281 | C17orf76 | CCDC24 | HCG26 |
| ZNF853 | ZNF90 | LGI4 | OR8S1 | ACHE | PRAMEF20 | LOC284801 | NRXN2 | HOXA3 | HIST1H4G | MTMR12 | SH3KBP1 |
| HORMAD2 | MVK | IQCG | MDC1 | GRIN1 | C16orf55 | NINL | CD177 | REG1P | ETV3 | OR51A4 | LOC100009676 |
| MACROD1 | ABCE1 | JPH4 | GDF11 | SPIN3 | WDR31 | FAM156B | KDM4A | PIK3CB | LOC100129461 | NAALAD2 | LOC646498 |
| EML2 | WBP5 | SCEL | LOC100129722 | BDKRB2 | AFF3 | RAB25 | LOC728114 | CCDC102A | EP400 | CYBRD1 | ZNF793 |
| BOLL | TBC1D24 | OR6C75 | GDF1 | IQCF1 | VPS39 | GATA3 | DGKH | PRKCB | SLCO4A1 | PHF20 | FAM159B |
| PTGES2 | CRISP1 | LOC652119 | LOC647323 | OR2T5 | OTUD7B | ZNF197 | TMEM14A | C9orf47 | MRPS34 | TMEM179 | TMEM55A |
| ZNF619 | CALN1 | LOC414300 | S100A13 | FREM3 | TSPAN6 | MYT1L | CHRNA4 | TEX13B | SH3BP2 | CLEC2L | C19orf50 |
| FANCA | RHOV | KLK6 | LAMA5 | DBH | BIVM | PKD1L1 | SERPINB4 | STX2 | GPR56 | LOC100653060 | GP5 |
| FAM20A | OR52N4 | DMRTC2 | NUDT2 | OR2L2 | SLITRK6 | BMP6 | TXNRD2 | PDE1A | ZNF536 | FZD7 | ADC |
| MYSM1 | LRRK2 | RRP1B | CX3CR1 | CAND2 | DKFZp566F0947 | MYL4 | DEPDC7 | BTBD17 | SLC30A2 | UBE2QL1 | GJB2 |
| ATF7IP2 | GDAP1L1 | OR5AR1 | LOC285548 | LOC541471 | KRTAP15-1 | LOC400706 | EPYC | ZNF544 | XAGE1A | ARL5B | EPX |
| CD300LF | EPHB1 | KTI12 | LOC100130433 | C1orf94 | CAMK2A | CYR61 | FOSL1 | CBY3 | POLR3F | HP1BP3 | KCNK13 |
| STT3A | DUSP4 | PKIG | LRRC37A3 | SLC35A3 | MAGIX | EMB | ANXA9 | MTRF1 | LOC646736 | MYH6 | GNAL |
| HAO2 | LOC387720 | P2RY8 | HNRNPUL2 | FLJ36000 | EGLN2 | PDAP1 | CCDC3 | NMUR1 | ADAMTS18 | DLG5 | NR6A1 |
| FNIP1 | C7orf13 | ARHGAP24 | MAFA | APOA2 | SFXN5 | DUOX1 | THEM4 | CD24 | C9orf139 | SH2D1B | GPR182 |
| LOC283214 | CLYBL | LOC100506310 | DLX4 | LRSAM1 | LRRC23 | COL9A1 | LOC100129648 | VPS54 | LOC400794 | HIC1 | MAP3K13 |
| WDR86 | SGCZ | MDGA2 | LOC283332 | PDLIM2 | CCND2 | SYNE2 | KIAA1967 | DPF1 | LOC100131907 | ZFHX4 | KCNJ2 |
| RNASE11 | ACACB | RAB17 | DKFZP434K028 | GARNL3 | NARS2 | BLOC1S3 | TMEM116 | C1orf68 | NUDT10 | ATXN7 | LOC100132593 |
| CRKL | ARL9 | P2RY13 | IQSEC2 | NOL9 | LOC400568 | TFF2 | LOC100505890 | APOL4 | LGI2 | ZBTB20 | PMM1 |
| SERGEF | SLPI | OR10S1 | FAM175A | ZSWIM4 | ABCD1 | ZKSCAN2 | CREBZF | KIF13A | MAGEA2B | PRAMEF7 | FEN1 |
| VN1R2 | REC8 | INSL4 | HEYL | IL1R2 | LONP2 | ESRP2 | ARHGEF16 | LOC100130373 | EID2B | RIC8A | LOC388849 |
| ARID5A | PCDHGB8P | MED13L | GPATCH1 | HDAC9 | GAL3ST3 | SPAG17 | IGFBPL1 | WDR66 | SST | DPP10 | P2RY2 |
| LOC723809 | LOC257358 | MAL | NOTCH1 | ADHFE1 | HIPK2 | CMTM4 | HSF2BP | SCARNA22 | GRID2IP | GLT1D1 | FITM2 |
| MTMR7 | GAMT | PLA2G2A | HIPK4 | LOC284661 | ASCL4 | DNAJB13 | MEOX2 | A4GNT | PROK2 | TMF1 | ACCN5 |
| NAP1L5 | ARSH | MC4R | MYLIP | NPHP4 | INA | TBC1D10C | AGR2 | AOC2 | TPST2 | SLC45A1 | PAX7 |
| FEZF2 | FAM189B | CENPC1 | KCNA2 | CCDC108 | SLC44A4 | HSPC072 | C8orf22 | PLEKHH3 | SPTLC3 | CAPRIN2 | ARL15 |
| EFNB1 | RHOBTB2 | XCL1 | ZIM3 | PDZD3 | BIN1 | DEFA10P | B3GALT1 | RAMP3 | VSIG10 | CMTM1 | C18orf26 |
| KIAA1324L | C12orf59 | ACVR1C | RANBP6 | CCDC137 | FAM160B1 | DDX53 | CLUAP1 | IGFBP4 | SLC30A1 | JPH2 | LOC200772 |
| CRCP | ENAM | SEMA3B | COQ3 | SHISA4 | PRAMEF2 | MC3R | IGFBP7 | CKMT2 | NLN | TSSK6 | C15orf5 |
| PHKA2 | TMIGD2 | TMCO6 | LY6G6F | SUCNR1 | GPR116 | P2RY14 | NADK | ATF5 | C12orf74 | TRIP13 | EMR1 |
| INTS2 | ZFP41 | TECTA | PNPLA2 | CYP11A1 | PRAMEF3 | VENTXP7 | ROR2 | LOC100131432 | MUC5B | PABPC1L2B | TRPM4 |
| TEX11 | ZNF498 | PLB1 | SLC22A7 | RTDR1 | CCDC48 | HTN1 | OR10R2 | LOC100128869 | DPEP3 | EML1 | C5orf60 |
| FAM69A | FAM83B | STAC2 | ALB | DTX1 | TLK1 | F13A1 | LOC100130580 | LOC147804 | OR7E156P | PXDNL | MESTIT1 |
| SLC38A8 | PGAM2 | VPREB3 | INPP5J | VWC2 | MATN3 | FAM149A | LOC100286979 | UNC13C | C1orf105 | SLITRK3 | ETV5 |
| DSEL | BRF1 | XPC | PTGR2 | C8orf44 | CIB4 | RNF2 | KCNJ5 | CDH24 | NKX1-2 | C3orf33 | ZNF516 |
| RRBP1 | SNHG8 | KCNJ12 | CEACAM5 | TMEM74 | TESK2 | OR2T2 | LOC254100 | HOOK2 | PPP1R3G | UBA5 | DEFB131 |
| HIVEP1 | ZNF771 | LPHN1 | GOLGA8F | SELENBP1 | ZSWIM3 | LOC100132735 | ANKLE1 | ADRA1A | SYT16 | CAMLG | SLC13A2 |
| PF4 | TMEM200A | DDX43 | AWAT1 | GK2 | HTR3D | C1orf55 | OR7D4 | SLC6A3 | CAB39L | ARL3 | LOC728671 |
| GPHB5 | HHLA1 | CDR2 | GPR111 | KIAA1024L | ZNF142 | TMEM222 | C11orf40 | C12orf73 | GPR162 | LOC100134713 | MXD1 |
| GPR22 | NFE2L2 | NT5C3L | PLAGL1 | FAM151B | LOC388152 | PI4KAP2 | WRNIP1 | ATXN8OS | RAG2 | C12orf29 | LRRC31 |
| ACTL7A | VRK1 | C17orf85 | POM121L4P | TMEM125 | MRPL43 | CCBE1 | GPC2 | TMED1 | DPY19L3 | FCF1 | CAMK2N1 |
| OR5AC2 | C7orf31 | DKK1 | PKIB | C6orf136 | OR4N5 | LRP12 | LOC285422 | PKP1 | SH3BP1 | NAV1 | ZC3H12C |
| DKFZp451B082 | PNOC | DBF4 | SMG6 | LOC284939 | CPB1 | OR4D10 | SFN | HIST1H2AE | KCNRG | MTNR1A | ILDR2 |
| BOK | CXCL13 | WHSC2 | ZBTB22 | ZP1 | HDAC11 | KRT71 | FBXO18 | GANAB | OR1L4 | COL4A4 | IL33 |
| INTS5 | ORAOV1 | HERPUD2 | SLAMF6 | HKDC1 | PKD1L3 | KCNC1 | C12orf75 | GUCA1A | CEP72 | ZNF41 | NPW |
| S100B | USH2A | LOC100128881 | NCR2 | TTC31 | SRF | OTOP1 | NTN4 | MRPL23 | PPP2R5D | HMCN1 | LOC100128126 |
| OLIG1 | PRSS3 | OPTC | KIAA0564 | PLSCR5 | DYRK4 | ZNF276 | TLX2 | PTCHD1 | ANP32D | ARID3A | UBXN11 |
| TIGD5 | OR1I1 | XRRA1 | RAD9B | WDR27 | OXNAD1 | SPATA18 | CSGALNACT1 | FTSJ3 | FAM117A | KCNJ3 | SEPT3 |
| FAM57B | LOC728084 | KIAA1161 | LOC100130931 | MAST1 | COX15 | FAM65B | RBM42 | TRIP10 | FAM71C | PVRIG | LCN6 |
| ASGR2 | LOC284395 | HES3 | IL8 | ALDH6A1 | ERP44 | LOC284751 | ZNF180 | LOC100129324 | SLC36A4 | TBC1D25 | BTN2A1 |
| UTS2 | CDH15 | SHISA2 | CCDC7 | CAPN7 | LOC729668 | TNXB | KPNA7 | C17orf58 | FLOT1 | VAT1L | C8orf50 |
| COL15A1 | PCDHB2 | DEFB103B | PSAT1 | FHDC1 | RIPPLY1 | ZNF618 | LOC440700 | LRP3 | EFNB2 | RSPO3 | FRMD4B |
| TMEM158 | TSR1 | CERK | LRCH3 | SDC1 | PNPLA7 | NOL3 | SH3BGR | LOC100128160 | RAD54L2 | LOC100128950 | AQP1 |
| HMGCL | CD72 | CABYR | CDR1 | IGSF5 | NPBWR1 | TJP2 | PIK3R4 | CCDC148 | FCRL1 | ADNP | FGD1 |
| ZNF329 | SUSD4 | CASP10 | ALOX12B | LY6G5C | FER1L6 | PRAME | ARMC9 | NEIL2 | ZCCHC16 | PABPC5 | TPTE |
| LRRC8E | NIPAL1 | HIST2H3A | NUDC | C1orf133 | ANTXR2 | ITPK1 | NDUFB10 | ZNF615 | NINJ1 | FCER2 | DCST2 |
| LST1 | TANC1 | CST7 | MNX1 | UCK2 | NKPD1 | ZNF282 | SLC35B3 | CD79B | LY6H | CDC42EP3 | LOC503519 |
| AP4S1 | TTN | GPC4 | POU3F4 | LCE5A | SLC1A7 | USF1 | MGC16703 | POSTN | ZNF134 | LOC100132207 | LMX1A |
| NRF1 | FBXO36 | FLJ33630 | SCLY | BRPF3 | HTR1F | CLDN10 | ATP1B4 | LOC286177 | LRP2BP | SLC4A1 | GPR78 |
| LOC100652742 | LOC283501 | GLT8D2 | HIST1H2AB | ADAM8 | SLC37A3 | ADAMTSL2 | ATXN8 | GPR179 | SERPINB5 | FAM134C | ZGLP1 |
| C8orf37 | AQP6 | SERPINB11 | FAM25C | C17orf105 | SEMA7A | GLYCAM1 | TBXA2R | LOC728065 | MGC2752 | CAMK1G | FAM161A |
| FYB | PLXNA4 | DEAF1 | DLX2 | SLC1A4 | MS4A2 | GLRA2 | GNG11 | KCNJ6 | R3HDM1 | TRIM42 | FOXN1 |
| DCLK2 | HIST1H2AC | EXPH5 | C14orf135 | RANBP1 | PHLDA3 | INTU | C14orf34 | KCNN2 | PTPN13 | KIAA1407 | SMAD6 |
| LOC646626 | RAD17 | TFEB | LOC221122 | TIMM10 | GRIK3 | LOC284513 | MAT2A | EMP2 | LILRP2 | CCDC54 | C16orf80 |
| TCEB3B | CHST6 | SLFN14 | FAM110B | FLJ43860 | APOH | RWDD2A | FJX1 | HIST1H4K | PLAT | F2RL1 | CD247 |
| SGOL2 | SYT6 | SPRR2C | LOC283140 | DAZL | HSPA13 | LOC729291 | XKRX | LOC644277 | LOC100130920 | ZNF280D | MIF4GD |
| TLX1 | LOC727869 | TMEM65 | CAPN13 | OR5AS1 | ZCCHC23 | LEPREL1 | GNAT3 | LOC730974 | PRAP1 | MTA3 | FAM110C |
| EIF2C2 | MIR155HG | TNKS2 | GOLM1 | BIRC7 | LENG8 | FBXW9 | TFAP2A | DAGLA | DQX1 | HAMP | CCDC76 |
| SARDH | MRGPRF | CENPL | KCNIP1 | LOC100130264 | CCDC141 | LY6D | PRKCA | S100A1 | GABRA2 | FAM47A | GTF2IRD1 |
| SLC12A1 | BCAS4 | HK3 | ELOVL2 | C3orf55 | KCNC4 | SERPINA5 | OR2M7 | ITGBL1 | CPA6 | CNTFR | GPR146 |
| MET | LSM14B | ALDH18A1 | LOC729870 | ARC | KRT34 | TDRD10 | DIP2C | HTR7 | LOC100130657 | C11orf51 | LOC100132273 |
| ZBBX | LGALS16 | INSL3 | OTOG | MRPS22 | PHKG2 | LTB | SLC45A2 | GSTM4 | CCNY | MED15 | C9orf140 |
| ZFP3 | C4orf21 | TACR2 | FGD5 | DEXI | CBLB | ZNF512B | LOC100128242 | PROP1 | SLC30A6 | ADAM6 | PRR12 |
| MIMT1 | SAMD8 | OR6A2 | LOC100652762 | PYCR1 | ERLIN2 | CDY2A | PITPNM1 | MGC15885 | PRAMEF22 | FAM169A | C21orf56 |
| NRG4 | OR4N2 | SIN3A | MEF2D | CCDC65 | RIT1 | CTDSPL2 | CLCF1 | KLK8 | IFLTD1 | DMBT1 | KRTAP13-3 |
| ROR1 | CEP57 | LOC284551 | CCT5 | STAP1 | APOC3 | NOL12 | C6orf145 | PLCL1 | LOC100129894 | EML6 | NANOS3 |
| C18orf21 | SMYD4 | IPO9 | PDE4DIP | LOC730811 | GPX6 | TMC4 | HSFY2 | DTX4 | NDUFA6 | NFATC4 | LRRC33 |
| HEMGN | C2orf49 | MLXIPL | C9orf71 | ZNF10 | LOC285819 | LOC202181 | C9orf106 | ADAM32 | PTTG3P | TRIM72 | MXD4 |
| C12orf12 | YAF2 | MT1M | GSTA5 | FBXO38 | PPP4R1L | LOC100133145 | FAM151A | GNA14 | C2orf81 | CLEC1A | MAGI2 |
| RFTN1 | NOTCH4 | CER1 | SPDYC | RAB43P1 | ATP8A1 | HSPA1L | MYLK3 | MARCH9 | GUCA2A | PLLP | ZCCHC24 |
| LOC283867 | HIST1H2BK | LOC100131195 | MUC3A | KATNAL2 | SLC35F4 | BCR | DNAH7 | ERI2 | RBMY1B | ZDHHC1 | ZNF192 |
| CHAC1 | RASD1 | PPFIBP2 | SPR | ZNF876P | HUS1 | PLA2G7 | PLP1 | CPSF4L | GRAMD4 | AKAP4 | NDN |
| LOC100128640 | MMP7 | HOTAIR | C1QTNF1 | CLEC12B | ENHO | SLC25A31 | LOC201651 | NLGN4Y | FLJ40712 | NMBR | RTN4RL2 |
| ANKAR | C11orf9 | SHC2 | C4orf46 | AASDH | B4GALT6 | C1QTNF3 | SH2D7 | SEC61A2 | ZC3H4 | MARCH2 | MON1B |
| LOC401400 | PCDHA12 | KRTAP17-1 | AP1S3 | RASGRP1 | FFAR3 | GPR6 | OR5T3 | CDC20 | FLJ46320 | C3orf58 | CEBPE |
| DOCK2 | ACAP3 | FAM99A | FGFR4 | CSAG2 | ZNF785 | POMP | OMD | APOBEC2 | BEGAIN | LOC100128822 | MYO10 |
| CNGA1 | CLCNKA | NEK11 | LOC728073 | THEMIS | LOC100129888 | ANXA6 | HLA-L | SAMD12 | C10orf81 | STOML3 | IL23R |
| POLD2 | SYN3 | WWP1 | DACT1 | CCDC136 | TRIM16L | CD8A | CHIA | CENPBD1 | HTR3B | RIC8B | DHX16 |
| CREM | TNIP3 | KLRB1 | C1QTNF5 | QRICH1 | SRGAP1 | CANT1 | IL4I1 | THPO | TMEM229B | MCCC2 | YJEFN3 |
| TSPAN9 | C17orf107 | TAAR2 | AGBL2 | KRTAP10-11 | AMZ2 | FGF23 | LPPR3 | NDFIP2 | ZNF71 | TCTEX1D2 | ARRDC2 |
| LOC399715 | ENTPD4 | LOH12CR2 | RNF125 | UCHL1 | P2RY4 | SDC3 | C12orf50 | SESN3 | ZNF148 | S100A7A | SYNGR1 |
| UNCX | SEC14L1 | RHAG | LOC284263 | FAM190B | OR14J1 | SUCLA2 | DPEP1 | IRAK2 | C6orf221 | LOC100130761 | BTRC |
| ZNF776 | TNFSF8 | ZNF554 | CSF3R | KRTAP2-2 | KCNJ8 | HMHA1 | KCTD14 | TRIM58 | FRMD3 | STARD13 | ATXN7L1 |
| DHX33 | LOC96610 | SLC35D3 | NELF | MYOM1 | C7orf26 | KIAA1751 | FGF6 | JARID2 | TOPORS | SCGB1C1 | C9orf80 |
| EGFR | ZNF333 | FOXK1 | TOX2 | ZNF695 | KRT83 | BST1 | PTGDS | PGBD2 | HMX3 | MAP4K4 | TMEM192 |
| IL16 | HADHA | TMEM63A | CSRNP1 | C1orf229 | LRRN3 | LOC100144603 | MYO1H | TAS2R7 | TUBBP5 | IL18BP | GSR |
| C14orf142 | TRIM29 | SOX30 | ENTPD5 | DUSP5 | GABBR2 | LOC375295 | BRMS1L | LOC146336 | C6orf162 | RNF214 | METTL4 |
| ITGAD | PCDHB12 | NFKBIL1 | NCAN | LOC145845 | PCDHA1 | PRSS8 | ACRC | RHCG | UPF3B | GLIS1 | IAH1 |
| TRIM23 | DGCR14 | FAM18A | OR7E91P | ACER2 | SLC7A3 | STON1-GTF2A1L | PRAF2 | CEP97 | SCML1 | RNF128 | FGFBP1 |
| ANKRD60 | BMP7 | C9orf82 | ZNF385D | IPO5 | FXR2 | C20orf107 | C8orf84 | CCR2 | LOC401588 | OSBPL7 | THBS3 |
| FSCN1 | HSD11B1 | ANXA2P3 | TTC38 | FLJ30838 | ABHD13 | OR2T10 | GPRC5A | C20orf195 | ZMYND17 | OR8J3 | C9orf148 |
| OR9A4 | CYP27B1 | RAB3IP | OLFML3 | ERP27 | RIN1 | GLYR1 | ADAM23 | CFHR2 | JAK3 | NDOR1 | PHF2 |
| PGLYRP2 | GATM | ANKRD62 | FAM154B | GGT5 | TMEM41A | LRMP | RGS9BP | GPR171 | OVOL2 | SDF4 | THAP9 |
| HRH4 | DIRAS2 | DOCK6 | PDE10A | ARMCX3 | SHBG | PCDHGA2 | NHSL1 | METTL14 | AGPAT6 | CD163L1 | SIGLEC10 |
| GRID2 | LOC255512 | LOC100132481 | VSIG2 | TULP2 | DAGLB | FGFR1OP2 | TUFT1 | PI3 | LPHN3 | STARD6 | RETSAT |
| OR4C46 | USP49 | NOTO | CNIH3 | STARD4 | HDAC5 | PGGT1B | KCNK5 | ACADL | INADL | LOC100132147 | LARP1B |
| LOXL2 | C20orf134 | BGLAP | FAM115C | KRTAP5-7 | SEC1 | CLDN5 | MMP9 | RAPGEF5 | FLJ90757 | DEFT1P | INPP4B |
| C19orf38 | BMS1P4 | SULT2A1 | HBM | IL13 | WDR36 | PER3 | RUFY3 | CH25H | TNFAIP1 | LOC643623 | USP37 |
| DPY19L2 | PCDH7 | SLC5A3 | TNFSF4 | GOLGA4 | ZNF674 | LOC401127 | PILRA | MGC50722 | DCT | OBFC2B | PXK |
| FAM50B | HIST1H2AH | KRT40 | LOC440040 | GSDMB | TBC1D19 | EHD4 | PCMTD2 | TECPR2 | ITLN2 | TMEM184C | EPB41 |
| NSUN6 | MPP3 | FAM184B | NXNL2 | DTNB | ACRBP | LGR5 | NPL | PTPN20A | VAV2 | OR11H4 | TRDMT1 |
| KRTAP24-1 | CCL11 | KLRC3 | KRR1 | FZR1 | OR6C2 | GOLGA2 | FIBP | G0S2 | AZU1 | SMAD1 | FGB |
| ADAM19 | LOC256483 | NHS | UROS | NCAPH | KCNMB2 | SMC5 | GSTCD | FAM122A | PAQR3 | CABP4 | ALG14 |
| MC5R | RTEL1 | STARD10 | SNORA71A | C16orf7 | FEZ1 | FBXO5 | ACOT13 | MAPK4 | LOC340017 | NPY5R | AGXT2 |
| TCTE1 | LOC100132005 | FBXW10 | CDC42BPA | TP53BP2 | SLC9A3 | NR2F2 | SNORD116-19 | THBS2 | RG9MTD2 | PTPN9 | PRRG1 |
| TMPRSS11D | CLVS1 | IQUB | NCCRP1 | MAGEB17 | C20orf160 | TAS2R50 | LAMB1 | PSD4 | TMEM220 | LOC440346 | PDZRN4 |
| SPATS2 | TCF7 | SLC45A3 | NOVA1 | TRPS1 | PUS3 | FAM126A | INCENP | MXRA7 | LOC283028 | FAM176A | ZNF346 |
| KHDC1 | LOC400940 | SPG20 | SON | RBP2 | VAMP2 | TSPYL6 | TAGAP | FPGT | BICD1 | OTX1 | BCL7B |
| FOLR3 | PCDHB9 | DSG3 | SSX2IP | IGF2 | LOC100131366 | TRIM47 | SBNO2 | NAPB | HELZ | SH2D4A | CDC14C |
| SEMA4B | C19orf12 | PCDH12 | OR2B2 | LOC100130111 | CRNKL1 | RECK | ZNF114 | HDC | MYO7A | ESYT1 | OSGEP |
| ZNF528 | DTX2 | TMPRSS11B | C7orf34 | HOMER1 | BMPR2 | DCAF17 | KCTD15 | ZNF773 | SLCO4C1 | IL2RB | ADAMTS14 |
| LOC100132774 | PPIP5K1 | LPCAT2 | DPY19L2P2 | GFPT1 | DAPK1 | KRT6C | ZHX1 | ZNF484 | TSLP | GABRB3 | MFI2 |
| OR9A2 | RBBP6 | TRIB2 | PPAP2C | CCL15 | C11orf91 | SYNGR4 | PHF17 | WWP2 | GIMAP5 | EXOC6B | CCNYL1 |
| SCARNA13 | KIAA1609 | PCDHB16 | MLLT6 | TRIM63 | CLIP3 | RPS6KA2 | LINGO2 | FLJ39051 | MAS1 | SCNN1A | CCNT2 |
| FLJ44715 | ZNF740 | LOC730183 | LCN2 | PPARA | C7 | COL2A1 | MLN | SLC1A1 | EPPK1 | SFTA2 | ZSCAN16 |
| FAM27E3 | PYDC1 | TIMM8A | HLA-DPB2 | LOC100508384 | AKR1D1 | IGF1R | PAX5 | LOC100652763 | ZP4 | POLR3B | OSM |
| IPO7 | SCN10A | TXNDC5 | SGTB | FAM76B | TMEM54 | KRT74 | CHN2 | SULT1A2 | ENPEP | DKFZP761C1711 | RIMKLB |
| IFNA10 | GULP1 | FANCE | CCL3L3 | MPDZ | YPEL4 | KRTAP21-2 | FLJ26086 | LOC644450 | MTHFSD | NANP | VWA2 |
| ZKSCAN3 | KCNMB3 | MGC70870 | CTNNA3 | MAP3K12 | MAB21L1 | CDC42EP5 | TRERF1 | FGFR3 | AGTPBP1 | ADCK1 | TMPRSS13 |
| GHRLOS | C2orf16 | DIS3L | MOBP | TSNARE1 | RBM12 | RGR | DHRS3 | CYP4B1 | DUX4 | TARSL2 | NEDD4 |
| TLR2 | IFNB1 | CYP4F11 | C2orf40 | CHRNG | CLCC1 | NKX2-5 | PTX3 | KRT84 | AZI2 | CDRT8 | GALNT12 |
| ODF2 | LAX1 | NBPF10 | HPSE2 | MBLAC1 | OCLN | ADCY4 | KDM6A | TAF2 | USP28 | LOC390705 | KCTD18 |
| GALR3 | CRY1 | PCSK1N | LOC643551 | FAM71E1 | ROPN1 | C12orf49 | UTP20 | LOC100131496 | EPHA8 | MAN2B1 | C1orf190 |
| TMEM88B | SLC5A7 | ZNF557 | FRS2 | CARD17 | ZNF497 | OR2L13 | WDR85 | LOC100128131 | ZNF311 | PHLDA2 | CYFIP2 |
| KIAA1671 | LOC729176 | SUPT16H | CCDC149 | SNAR-E | TFF1 | WDHD1 | GALNT3 | EP400NL | H2AFY2 | ARHGEF1 | CNTN5 |
| ZNF709 | IMPG1 | RPA4 | LOC731656 | RAVER1 | EBPL | GRIA4 | DPP3 | NOS1 | FLJ22184 | NR1H4 | TUBA4A |
| C3orf15 | ANGPTL5 | C1S | LOC100132069 | LOC285762 | ZFAND4 | SCML4 | HCN2 | PLA2G16 | LMAN1 | AADACL2 | RELB |
| TAS2R1 | B3GALNT2 | CHRNB2 | POR | KIAA1211 | DNAH14 | STH | SNX25 | NME4 | CYP2D6 | C22orf39 | GPIHBP1 |
| LDHC | YAP1 | CDO1 | IL5RA | MYL7 | MAML1 | LOC148696 | WDR90 | CELSR1 | C7orf54 | SP2 | TECTB |
| LOC643723 | EPS8 | CD2 | RGS7BP | ZNF212 | PLEKHA4 | METTL6 | HMGA1 | FLJ42969 | CATSPERG | CD96 | OR4K13 |
| DMRTA1 | GUF1 | CASP12 | PLK4 | TTLL10 | C10orf140 | TRPV3 | SLC22A1 | PRR25 | NKG7 | TTC32 | PELO |
| WBSCR27 | BCL11B | MEG3 | LOC100128001 | C14orf183 | GPR153 | CDR2L | RAB3B | CCL21 | DCLK1 | LCK | KRT33B |
| SRC | KDELC1 | SDAD1 | CFLAR | AFG3L2 | LOC387647 | ACPP | ZCCHC5 | TRIM7 | PRRT3 | ABCA17P | TMPRSS12 |
| PROZ | SCN1A | PROCA1 | ATP6AP1L | CLIC6 | S100A3 | MED19 | C4orf7 | LRRC48 | PNRC1 | ASS1 | TMEM89 |
| CDC37L1 | ZNF880 | GPR151 | PHLPP2 | BEX5 | EDEM1 | PRAMEF8 | LCE6A | SNTN | NANOS2 | PRR5-ARHGAP8 | CST6 |
| CA5B | ANO8 | ELL | ZNF107 | SLC25A22 | SNAP91 | SLC9A11 | RNF32 | TAS2R39 | C1orf143 | CTNNA2 | CD101 |
| ZBTB16 | POTED | CD274 | TTTY23 | SERTAD3 | CNBD1 | C19orf54 | MS4A5 | DHPS | HHIP | SARS2 | CXCL1 |
| IPMK | RSPH9 | ZNF117 | SNCA | TCEA2 | YES1 | APH1A | GPR1 | BMP2 | LCN10 | APCS | ZNF799 |
| CCDC112 | MYO16 | RD3 | FAM59A | ABCB9 | NFRKB | HMGA2 | LOC644366 | BCL2L2 | TASP1 | FBXO31 | FRRS1 |
| LOC100129502 | COQ10A | LOC100130276 | CHD1 | SULF2 | LOC728769 | ZSCAN5A | ISG20 | SNAP25 | CAGE1 | ECT2 | HIST1H3B |
| HOXA6 | VMO1 | RAB11A | TIGD4 | PLK1 | ZACN | USP54 | MMP17 | ZNF273 | FAM66C | PRIM2 | ZFAND2B |
| NDUFAF2 | CYP1B1 | RELA | HKR1 | SFMBT1 | SLC18A1 | CALML3 | PRMT10 | C16orf92 | TK2 | C20orf201 | ACN9 |
| ZSCAN12 | SCARF2 | MLYCD | FGF9 | EML3 | LPAR1 | DSCR4 | C9orf123 | PDIA4 | F2R | SPRR3 | LRRC3B |
| GCNT7 | ZNF852 | RAB27A | ZDHHC11 | FLJ33544 | PMCH | DKFZP434L187 | CDH22 | SPAG5 | RUNX3 | LARP6 | TLE2 |
| FAM188A | C14orf162 | GLYATL1 | SLC13A5 | WAC | MORC4 | PLCXD1 | NOS3 | NPBWR2 | EXOC4 | ZBED2 | LOC729047 |
| IFT88 | MAP9 | CLCN1 | ZNF576 | PYGB | LIG4 | FLJ32756 | ZNF559 | GALM | PCNXL3 | RAB18 | C14orf49 |
| VSIG1 | LAMA1 | TEX9 | SUDS3 | ARHGAP9 | PPIL4 | LOC727924 | TFF3 | TMEM39A | C1orf210 | H19 | RAI14 |
| LOC730081 | KCNG3 | SLC25A10 | MURC | GABBR1 | LOC643770 | CD14 | LRRC38 | HDAC8 | KRT72 | ZNF512 | CDC5L |
| IGSF3 | LOC339442 | SAAL1 | C10orf67 | LOC100128139 | LOC100128670 | GUCY1A3 | NEUROG2 | VAV3 | ST14 | CDA | LOC730227 |
| C18orf23 | TMEM204 | EFNA2 | GPER | LATS1 | VSX1 | SPTLC2 | PTGS1 | MORC2 | REREP3 | WBP2NL | ADSL |
| LYNX1 | OR10G4 | CNGB1 | C14orf132 | CDH23 | SOD2 | ZNF20 | PRKAG3 | PTGIR | SFTA1P | CAV2 | NGFRAP1 |
| C6orf192 | LOC400128 | TFAP2D | LOC400499 | POTEF | ADCY10 | USP21 | UPK1A | NEK7 | ATXN7L2 | TNFAIP6 | SNAI2 |
| ZNF829 | ISLR2 | KIF22 | C17orf100 | CPNE8 | CISD3 | HDX | C1orf204 | C10orf129 | LOC126536 | DEFB124 | STAG3L2 |
| CYP27C1 | DRD5 | HELLS | HIST1H2BL | ZNF286B | DIO2 | DDX46 | LOC100288428 | ROBO1 | RBM4B | PLEKHG5 | APOL5 |
| ZNF167 | SPHKAP | TMEM132B | ACTR10 | LOC729652 | C9orf7 | GPR114 | KCTD19 | TIFAB | EYA3 | QSOX2 | LOC340037 |
| LOC100129794 | DLC1 | MMRN2 | MYLK | GPR25 | LEF1 | CCDC78 | MST152 | ZBTB8OS | EHF | SERP2 | CAPN8 |
| BACE1 | KLHL36 | FLJ27352 | FLJ16124 | NOS2 | CORO1A | UBXN2A | ZNF780A | PAK1IP1 | DKK2 | ATP9A | KCNQ2 |
| DSCC1 | EVX1 | MMP2 | SPRR2E | LOC100130285 | RNF182 | CHPT1 | CSRNP2 | GHSR | IGLL1 | RGS22 | C17orf70 |
| EPS15L1 | LOC100289650 | SERAC1 | DENND2A | SLC7A1 | PLEKHO1 | SNIP1 | ZNF813 | IBSP | APOLD1 | SURF6 | SCAND3 |
| IRF2BP1 | LOC375196 | HS3ST3B1 | ELOVL5 | TATDN1 | AQP5 | PPM1K | ALDH9A1 | TUSC3 | LRRC52 | PURG | TM4SF20 |
| SCN5A | TET1 | GUCY1B2 | SLK | C22orf15 | PPP4C | ASB9 | SPATA9 | LOC731932 | FOXRED1 | ZBTB3 | JRKL |
| CCDC8 | GABRA6 | FYN | KRT31 | ID3 | PRKD2 | ZFYVE20 | CLEC6A | DAAM1 | DAPK2 | MKRN7P | INSL5 |
| NCKAP1 | FZD2 | VN1R4 | ECEL1 | KRTAP26-1 | C1orf192 | LZTS2 | ZNF800 | GRM3 | CD80 | NPHP1 | ZNF653 |
| CPSF1 | PROS1 | LOC100127994 | RRP15 | LOC84989 | UBE2N | DUSP5P | LOC113230 | SIRT1 | ZMYND11 | AP1M2 | ELF2 |
| TMEM176B | PEX5 | FZD4 | NRAS | ZNF100 | C1orf151-NBL1 | DLGAP5 | LOC100128651 | DMRTA2 | LIPG | CD34 | CCDC42B |
| FLJ45872 | CEACAM19 | CNPY1 | GAS6 | SLC23A2 | LOC100127961 | CLDN9 | DNMT1 | EBF4 | DSCAML1 | HIST1H4B | ASB15 |
| ATG9A | FAM47E | MPP5 | ARVCF | CNO | ARL4D | SERPINE3 | C7orf43 | HMX1 | RPGR | METTL2A | DLAT |
| FERMT3 | PTPRZ1 | ZNF569 | MAP2K5 | PKNOX1 | SMURF1 | DGKZ | C2orf76 | CHST3 | MAST2 | LOC729681 | TMSB15B |
| CDX1 | MLPH | BEST4 | TUBG2 | CEACAM3 | LAMB3 | FRMD8 | CKAP2 | LBX1 | ZNF678 | WAS | TMEM167A |
| FLJ32255 | CHD7 | SCGB2A2 | ZNF77 | SEZ6L2 | IRX6 | HIST1H4A | GALNTL6 | INMT | SNRNP40 | IFNAR2 | SPINK7 |
| PID1 | ZFP30 | SHROOM3 | FAM167B | DPF3 | RAP1GAP2 | TRIM8 | ELFN1 | C7orf72 | LPAR2 | C4orf39 | OTUD4 |
| LRRC8B | PDE2A | TRIM32 | PLA2G2C | RFC4 | DNAI1 | ERN2 | TUBGCP4 | TMEM68 | SRY | FAM71F2 | ZNF517 |
| KIAA1045 | TMEM206 | INE2 | CALR3 | PMS2L2 | PEX12 | HSD17B6 | KCNA4 | STAM | FOXP4 | TMEM17 | LOC645967 |
| PGR | KRTAP13-4 | LRBA | MBNL2 | KIAA0895 | LILRB2 | DUSP15 | SOX15 | LOC645202 | UBASH3B | PIP5K1B | CHERP |
| RBM27 | WDR35 | PARP11 | GALNT7 | JMY | TBC1D8B | PFKM | CSN2 | ZFP64 | INO80B | PRND | FZD1 |
| SGMS2 | TMEM115 | FMO2 | LOC100499221 | GPR113 | NFXL1 | C18orf10 | PGAP2 | LENG9 | COPE | VN1R5 | KREMEN1 |
| CASKIN1 | PGLYRP4 | LOC339593 | C8orf73 | SPAG4 | MCOLN2 | TPRG1 | STK38L | LMO3 | ARAP3 | LOC286114 | TAS2R9 |
| ALOX15 | UBA6 | DGCR8 | DNM1 | GPRC6A | MBOAT2 | ADARB1 | CEP290 | WNT5A | CRK | ZNF28 | SLC22A24 |
| LOC100131726 | ANGPT2 | ERLIN1 | C12orf60 | KIR3DX1 | LOC157381 | RNF145 | NIPAL4 | MYRIP | TNFRSF21 | FHL2 | TYRP1 |
| SOS1 | MYO15A | TNFRSF9 | USP10 | PLIN4 | C6orf35 | FAM57A | KRT19 | MYO19 | PIK3CD | PLEKHG4 | LECT2 |
| PUS7L | NUP214 | ASB13 | VANGL1 | CASR | MCF2L2 | CPNE3 | CUL7 | ATP11B | ADAL | CENPP | UTP6 |
| KIAA1143 | RIMS3 | GLE1 | TCERG1 | IL1RL1 | APAF1 | LOC100128081 | MYT1 | NEK3 | FAM180A | ZNF323 | ZNF713 |
| THADA | CCNO | SBSN | SMAD2 | CEP250 | CDC37 | LIX1 | ZYG11A | FRAS1 | USP6NL | FNIP2 | ZNF22 |
| SPRY4 | MIS12 | LRRC70 | TNRC6A | DOCK4 | ZNF75A | NOSTRIN | FOLR2 | CD1C | TEX12 | MYCBPAP | LOC100287314 |
| LPA | C8orf38 | HOPX | DIABLO | ABCG1 | FSD1L | ZFP1 | ANKRD27 | ASPG | GGPS1 | LOC200609 | PLAA |
| TMEM151B | TTK | ZNF814 | FTMT | MBL2 | JPH3 | EVC2 | LOC100130057 | GATAD2B | NMI | NELL2 | GPR124 |
| ZNF441 | GKN2 | IRX3 | TBX10 | CXCR4 | BTBD16 | BRI3BP | PRAMEF19 | TK1 | SCOC | SERPINA9 | WDR19 |
| PDCD7 | FAM187B | PIK3R5 | ERI3 | C9orf37 | PHF20L1 | LOC100506190 | SLAMF1 | PAPSS2 | CEL | FBRSL1 | TRAF4 |
| STARD5 | TMPRSS9 | LOC100128517 | SLC36A1 | SPATA2 | RSPO2 | LOC100127972 | SLC38A6 | LRRC69 | PTPRS | LDOC1L | KIAA0240 |
| CCDC60 | PRLR | ACBD4 | GABRG1 | RAC3 | TJP3 | C9orf30 | ZAP70 | LOC100128063 | EPC2 | UAP1 | ANXA8L2 |
| UST | PANK1 | NFYB | MEGF10 | SSX1 | C6orf103 | ETV3L | RSL1D1 | PDK1 | LOC100130172 | NDUFB5 | KLHDC1 |
| C5orf38 | TAS2R3 | REST | BHLHE40 | ZSCAN18 | UNQ6494 | C5orf47 | MED21 | LOC100129936 | PPAP2A | TPD52L2 | LOC283038 |
| TSPAN1 | GSTA4 | C7orf10 | CTRB2 | HNRPLL | CP | C2orf63 | GPR115 | KIR2DS4 | KIAA1539 | MSTO1 | COL24A1 |
| CTLA4 | MMP10 | RXRG | PCDHB8 | UBE2Q1 | PFKL | LOC386597 | ZNF791 | ARRDC3 | PDS5A | LINGO4 | HAPLN2 |
| LHX3 | ATF7IP | RDH11 | PSMD5 | NUP35 | OR5M1 | ACAD10 | TRIM37 | LOC154449 | ADAD1 | SMAD3 | CFI |
| FOXM1 | FAM132A | SPIN4 | LOC150381 | ANKRD28 | MED22 | OR9H1P | GEM | CYB5D1 | LACTB2 | LOC283856 | ALDH1A3 |
| CACNA1G | HHLA2 | EFCAB3 | MALL | NES | TFE3 | MELK | ADCYAP1R1 | PLK2 | TSGA10 | ANGPTL3 | PLA2G1B |
| IRF2BP2 | C22orf29 | ZWILCH | FOXN4 | ALMS1 | DPY19L2P3 | MORN5 | SLC16A9 | MTAP | DNTT | USPL1 | CAPN5 |
| HES6 | RAD51AP1 | CRNDE | PPAP2B | PCDHA11 | MBIP | RRAGB | HIST2H2AA4 | IKBKE | SLFN13 | SLAIN2 | ZNF486 |
| SH3BP5 | C17orf57 | EIF4E | LOC100129721 | C11orf53 | SCAP | PTGS2 | GMIP | POLA2 | LOC730091 | PACRG | PDE4B |
| SCN9A | LOC729770 | UMOD | ZNF592 | DCAF12 | AGPAT5 | ATP6V1C2 | LRRC3C | FLJ42709 | ADA | GNRHR | RHOT1 |
| G6PC | KIAA0195 | FKBP1B | TMEM233 | CDK8 | ITGA1 | PIP | SPRN | PLEKHA3 | EXTL1 | RNF138P1 | BRAF |
| EPB49 | RASL12 | ASCC2 | SNORA78 | FSTL4 | UBE2E1 | LOC400027 | USP45 | SLC5A11 | GNGT2 | HTR3C | CUL4B |
| SLC26A6 | UTS2R | CELA2B | PWP2 | FHOD3 | LOC100130314 | AQP3 | PATL2 | GNG7 | DRD1 | LMO7 | TRAF5 |
| KRTAP19-1 | ZNF460 | DMBX1 | C1GALT1 | LOC285857 | DUSP28 | MMAA | GRIN3B | HYAL4 | FAS | SPATA5 | RBBP9 |
| MICB | TGFB3 | DNAH1 | KLHDC9 | SIAE | PRMT5 | C3orf23 | AMN1 | PRNT | PRAC | BLNK | A4GALT |
| CLEC14A | HECW2 | C11orf73 | UNC119B | ZNF251 | LRP8 | PIGA | SFTPA1 | NUDCD1 | MLL4 | COL6A3 | LTF |
| ANKRD26P1 | LOC100289186 | TRAPPC2 | C9orf100 | PHLDA1 | C10orf118 | MAPRE3 | LOC285178 | SLC1A5 | ZNF91 | FRMD5 | CCNE2 |
| SAMD9 | LPPR4 | DNASE1L3 | CDC73 | MBLAC2 | ZSCAN10 | LRRC58 | ALPI | LOC284242 | C14orf70 | GOLGA8E | LYSMD1 |
| CSF2RB | LOC100128893 | PDE11A | UBAP2 | FLJ25694 | MYH7 | WHSC1 | NPM3 | LOC100128184 | LOC100131176 | PLEKHG6 | L3MBTL3 |
| EN2 | BATF | FAM183A | TAT | WFDC2 | MAOB | ABCD4 | ATAD1 | C16orf90 | GPC5 | NSL1 | LOC100128239 |
| LRCH1 | PCBP4 | PRDX4 | TIGD2 | ZNF236 | MARCH5 | EPB41L1 | CAMK1 | ANKRD46 | KLHL20 | HCFC1 | FAM22D |
| AGRP | KCNK9 | IGHMBP2 | RQCD1 | EPOR | ANKH | C7orf53 | ZPBP | FOXD4 | KLRK1 | C20orf141 | TSKU |
| GAPT | RCHY1 | NRBP2 | IGFBP5 | EXOC8 | SMR3B | FOXP2 | TPP1 | TKTL1 | SNAPC3 | CENPE | KLRF1 |
| KPNA1 | BTLA | CES1 | BRPF1 | TRIM54 | CCDC140 | LOC646471 | LCN15 | LOC151009 | SAMD4B | LOC100130238 | LRP1 |
| ZRANB1 | ABCA13 | PPM1E | SMAP2 | ANGEL1 | RPL27A | PDCL | PM20D2 | ZNF624 | TMEM121 | DDX52 | ELAVL4 |
| PRSS16 | PFN4 | SHISA6 | OTUD6B | OR7C2 | SLCO3A1 | POU4F2 | RPS6KB1 | KRTAP9-6 | MMAB | TAF9B | LOC100129373 |
| PRR3 | PREPL | MATN1 | UTRN | NGF | VCAN | C10orf2 | HSD11B1L | LOC541473 | KIAA1370 | RACGAP1 | GDPD4 |
| GABPB2 | PTGDR2 | CAPN9 | SELS | TCL1B | AMPH | SNX9 | SYNRG | C6orf38 | OR6B3 | C9orf172 | IFITM2 |
| FAM66D | PLCL2 | GJA4 | PRKX | MAPK14 | FGL1 | IKZF4 | SSBP3 | PPP2R5A | MGC16121 | SNORA60 | ZBTB25 |
| C1orf126 | C22orf28 | DPY19L1 | RNF114 | MEI1 | SNHG10 | ETS1 | ADH1C | APBB1 | MBD3L1 | DOCK5 | LBX2 |
| TTC28 | LINC00511 | GABRB1 | RBBP8 | FAM160A1 | OR6C74 | KIAA0947 | TOR1AIP2 | C10orf68 | SVEP1 | GPAM | TAF1L |
| DKFZp779M0652 | TAF5 | HECTD1 | CACNG3 | ZNF302 | BEX1 | BATF3 | LOC100131434 | LOC399744 | PHF6 | PRKAA1 | C1orf201 |
| KRTAP9-9 | ECM1 | FAM20B | ARL13B | ARID4B | C16orf71 | HBZ | GLYCTK | KIAA0556 | RAI1 | C20orf132 | CCNA1 |
| IL6 | TNFRSF1A | ZDHHC17 | CPEB2 | GPX2 | PIF1 | TRIM45 | DUSP10 | APPL2 | CCL13 | ONECUT3 | CORIN |
| ABCC9 | CCAR1 | LOC100130654 | C7orf29 | ADM | ARHGAP21 | KRT26 | ZNF431 | LOC100130557 | ZCCHC11 | SSX4B | ITGA3 |
| PACSIN1 | WDR33 | MTERFD3 | SLC24A1 | POPDC3 | DPH5 | OR4D5 | SERPINE1 | CALY | ANKRD23 | C11orf80 | DPPA2 |
| DIS3L2 | KRTAP10-7 | MPP7 | DYNC1LI2 | PAX4 | UBR3 | HPGDS | L1TD1 | ZCCHC14 | OTUD3 | SERPINB3 | ALDH7A1 |
| DUSP18 | SLC20A2 | CYP4F3 | BCL9 | LOC100128501 | ZMYM5 | PAPD5 | CCR8 | CSMD3 | TP53 | QPCT | TATDN2 |
| ALKBH8 | POU6F1 | PUS7 | DENND1A | SMN2 | XK | UNC5D | BAIAP3 | ALOX15B | RBM6 | NOTCH2NL | TMEM198 |
| GK | ZNF586 | ABCB8 | PNPLA4 | KCNAB2 | CA2 | MANSC1 | OVCH1 | PCDH11Y | LOC100128164 | USHBP1 | CERKL |
| FAM46C | SPINT1 | LOC441455 | PCGF6 | SLC35A1 | AASDHPPT | OXTR | CECR6 | LOC254312 | KRTAP3-1 | EPB42 | FLJ41455 |
| SAFB | LOC100131015 | FASN | MAGEA6 | SETD1B | GRAMD1A | TADA2B | DOCK3 | SNX4 | GPSM1 | DNAJC24 | PADI2 |
| C5orf13 | MMP26 | FOXG1 | AFF1 | RBM7 | NFAT5 | ZEB1 | TCN1 | TM7SF2 | JAKMIP1 | POLR3D | RHOD |
| LOC402779 | CPNE4 | TLX1NB | NNT | PEX6 | RNF144B | SMC1A | MAP3K2 | POMT1 | FOXRED2 | PKN2 | PDE9A |
| ICMT | SERPINB2 | KDM5C | GBP6 | ESAM | TCF7L1 | ADAMTSL5 | SLC5A12 | LOC100129312 | SNORA70B | NPVF | VTI1A |
| NOV | PCDHGA9 | AARS2 | INHBE | LOC283788 | MEGF6 | SUSD2 | LOC80054 | GALNT4 | C5orf51 | C1orf87 | USP27X |
| CRIP3 | NCDN | MBOAT4 | TAF1B | NUP50 | TGS1 | SLC4A4 | BMP2K | ZNF3 | GIGYF1 | IFITM1 | AREG |
| MAP3K7 | VTA1 | DEFB110 | MDN1 | C2orf85 | YSK4 | STMN4 | LOC642366 | PPME1 | TOM1L1 | PRO0628 | OAF |
| WARS2 | FETUB | C22orf36 | KIFC1 | KRTAP10-9 | SRP19 | EFEMP2 | OR10J5 | MAP7D3 | CNOT8 | FOXI1 | FAM3C |
| ZNF467 | AGMAT | LOC100131822 | NPPB | MSI1 | FAM72D | LOC100129316 | CENPK | LOC389831 | DLK2 | ZNF770 | FBXO3 |
| FBXO11 | FAM194B | HOXC6 | GSN | LYRM1 | C1orf109 | CX3CL1 | KRTAP3-2 | CYP4V2 | ADRB3 | PRKXP1 | PRKAG1 |
| LOC400685 | ZNF551 | FNDC5 | LOC100126784 | PATE4 | FCRLA | NCAM1 | LRRN1 | SLMO2 | CLEC4GP1 | ZNF25 | DCAF5 |
| FMO1 | ARR3 | EPSTI1 | LILRB5 | LOC100128843 | OSBPL2 | FAM54A | ACER1 | PACS1 | HAUS6 | HOXA10 | CMIP |
| C3orf75 | TCF12 | SPERT | PGS1 | KLHL25 | C2orf27A | SP140 | SERINC2 | ADAMDEC1 | PRCD | FKBP5 | ARHGAP11B |
| PRAMEF5 | DPT | CD2AP | PRRG4 | BHLHE22 | FAM198A | MYCT1 | ARL16 | SPTB | OLFML2A | TGM7 | DPYSL3 |
| METTL8 | C9orf128 | IKBIP | INTS7 | GUCY2F | FRYL | FAM99B | LOC143286 | LOC100129744 | ANLN | ATP2B4 | ICK |
| MYEOV | FLJ40292 | LOC284837 | MT1G | ZNF654 | STRADA | RUNDC1 | ZBTB47 | LOC100128077 | ATP1B2 | MPHOSPH8 | LOC100131825 |
| FAM95B1 | FLJ40852 | GAL3ST2 | FOXB1 | TSC2 | NFKBID | MAML2 | LOC100289079 | FLJ33581 | C8orf31 | LRCH2 | RAD50 |
| LIPC | HDAC6 | LOC202781 | OR4Q3 | PTCHD3 | ZNF566 | FNTB | KBTBD3 | CRP | RXFP1 | OR8D2 | PLEKHM2 |
| ATP6V1H | PLCH2 | RABGAP1 | MGC21881 | DBNDD1 | IL15RA | ABCB7 | ZNF578 | PLAGL2 | C1orf182 | CDT1 | CHST11 |
| CHAD | ZNF345 | SIPA1L2 | GHR | MSLN | BHLHE23 | AR | GFI1 | PITPNB | OR1N1 | DACT2 | ODF3 |
| LOC644686 | TMEM231 | MFN1 | LRRC34 | AHDC1 | GPCRLTM7 | REG4 | TAGLN3 | CEP120 | TMEM182 | ERCC4 | PYY2 |
| BLM | PIGB | C12orf35 | NUDT19 | MYO1A | CST8 | GAL | C12orf76 | FAM153B | TBCC | UTP14C | H1FOO |
| STAB1 | GRB7 | GSTM5 | TPPP3 | OR8B3 | ZC3H6 | MFAP2 | RB1CC1 | TACR1 | SYTL3 | C12orf28 | ZNF532 |
| C11orf52 | CTPS2 | SLC35E2 | CCL3 | LOC645752 | LPAL2 | BFSP1 | SLC17A3 | CNOT1 | ZNF367 | DNMT3B | C12orf70 |
| SDC4 | EYS | PHTF1 | CCL17 | RNF168 | LOC388948 | PLAC4 | IL17RD | BAI2 | SLC33A1 | H2AFX | SMAP1 |
| GSTM3 | SMPD3 | PKD1 | LOC283588 | ARG1 | BEST2 | MLF1IP | MRPL1 | RASA2 | FLJ30901 | TCP10 | ZDHHC6 |
| LCE1B | AMBN | CEP170 | EEF1A2 | GNAI3 | LOC100130456 | GPR82 | C4orf32 | LOC100128398 | SMPD2 | C18orf54 | SGOL1 |
| HES7 | AMDHD1 | LOC100190940 | RNF222 | GNAQ | FAM124A | TMEM45B | TTBK2 | KPNA5 | NSD1 | ZBTB26 | OR4N3P |
| LOC144817 | DCK | SLC46A3 | WDR49 | GOLIM4 | NEU3 | VIP | CDKN1B | OR52B2 | NCRNA00185 | CXorf30 | PRR4 |
| RAP2B | SACS | MRTO4 | ZNF189 | ZNF749 | KIAA0182 | LOC253962 | RFXAP | GLI3 | RAD9A | BRCA2 | FAM188B |
| C18orf45 | GGCX | C4orf33 | NCOA2 | LILRB4 | PPP2CA | PPP1R1C | TTC17 | NFKBIB | TPP2 | LOC153811 | GJA5 |
| SEMA3E | SKA3 | STIM2 | USP46 | LOC339666 | BSPRY | TSC1 | BCL7A | MAGEA1 | C10orf116 | CCDC110 | ZNF778 |
| ZRANB2 | PPP1R14A | GIMAP7 | ZFY | VEGFC | XKR9 | CEACAM4 | NUP210P1 | NAT2 | LOC219731 | SERPING1 | MKI67IP |
| COL4A5 | LOC644246 | CLASP1 | TTC22 | GRIN3A | EGFLAM | SNORA28 | INHBC | C1orf177 | CKMT1A | ZNF121 | ZBTB2 |
| C1QTNF4 | LRRC14B | PRTN3 | IRX5 | OR51I2 | FITM1 | SOX8 | MYOG | ANKRD32 | NAP1L4 | DHRS1 | WDR63 |
| PTBP1 | CFH | SEC24D | LOC400682 | KITLG | GOLGA7B | MUC4 | CNOT6 | HIST1H2BG | ATG5 | GCOM1 | TOX |
| ATXN1L | MT1DP | EP300 | ATP8B4 | SYTL4 | XPO4 | LRP1B | PDLIM1 | ZNF160 | UNC5B | WWC2 | PITPNC1 |
| BTBD7 | EXOC2 | AKAP12 | TTC3 | STAU2 | IRX2 | CALCOCO2 | OR5A1 | TDO2 | FMO3 | KCNIP4 | ZNF583 |
| YPEL2 | LOC100240735 | CLDN25 | GGNBP2 | C5orf30 | LOC645676 | EPN2 | ALAS2 | KCTD7 | CPA5 | CKM | DNAJC5B |
| CHRNA3 | ZNF449 | AGPS | MARCH10 | ZNF768 | SRCAP | AP1S2 | ETV1 | FABP4 | C6orf120 | SEPT11 | IRX4 |
| LOC648691 | TOR2A | DBT | FCHSD2 | LOC286437 | BAALC | CAPN10 | C1orf110 | EHD3 | TCHH | ABCB6 | STRN3 |
| GAD2 | SOD3 | UACA | PYROXD2 | FAM92A1 | ZNF480 | ZNF350 | IP6K1 | CD8B | RNU1-5 | RSPH1 | LOC100128343 |
| UGT2B10 | NUP210 | EMR2 | ECEL1P2 | C2orf61 | LOC100130468 | CASP7 | SPATA12 | CPD | LANCL1 | ZNF815 | HBD |
| ZNF267 | ACOX2 | RIBC1 | VEZT | LOC344887 | RACGAP1P | ABCA5 | BVES | DMXL2 | FAM102A | RGAG4 | ARF6 |
| TREH | TMEM190 | C20orf106 | MINK1 | TMEM165 | SRBD1 | ABCB11 | TLR3 | PCSK9 | RHOBTB1 | LOC100128531 | CRYAB |
| GINS1 | OR10C1 | OR2A7 | SOCS1 | OR10G2 | OR2F1 | PPP1R12B | HDGFL1 | TUG1 | PPP6R2 | ATP13A5 | ALDH3A1 |
| SPAST | DDX27 | LYPLA1 | CHD2 | ZNF529 | PAPL | MXI1 | FLJ45671 | TRAF7 | LOC400927 | LOC157562 | DIRAS3 |
| PAPOLG | CYP7A1 | ARSF | LETM2 | CTSL2 | SPATA3 | VPS45 | TBC1D4 | ZNF750 | XIRP2 | TMEM66 | FLJ39639 |
| TDRKH | SASH1 | CEBPD | C19orf47 | GPR75 | RANBP2 | DAG1 | CDK3 | FLJ23867 | KIAA1875 | CA13 | MSL1 |
| ZNF707 | PIGW | BRCC3 | OFD1 | TBC1D5 | ADPRHL1 | FBLN5 | FXYD3 | ZPLD1 | COMMD2 | FLJ34747 | CBX4 |
| LOC440082 | LOC100130673 | ETS2 | NBEA | DACH2 | GSPT1 | MGAT4A | BUB1B | ZKSCAN5 | SHQ1 | OR4C15 | SYDE1 |
| ABRA | LOC100131355 | LOC153684 | FAM181A | TMEM79 | SNHG7 | MTMR9 | SETD7 | USP19 | ANKRD22 | PGM2 | DUSP2 |
| KSR2 | GPR156 | FAM118A | GC | TIGD6 | SOCS3 | LOC100131283 | LOC150622 | LOC100133319 | DUPD1 | C4orf42 | APLN |
| ATG16L1 | ACER3 | SH2B1 | LOC116437 | FSD2 | BAGE | GPR45 | LOC100134237 | ANKRD7 | RNF146 | MPRIP | ZNF862 |
| OPN1SW | FA2H | GRAMD1C | EFEMP1 | TPST1 | GALNT14 | INHBB | RGL4 | S1PR1 | METTL7B | TRIM2 | MMP25 |
| ADORA2A | SDS | ELF3 | C19orf33 | IQCD | MGAM | AKAP5 | APOBEC3B | HS3ST2 | FAM83E | FAM184A | SPON2 |
| DEFA3 | TM4SF1 | C9orf125 | CLU | CNN3 | CKB | BCAS1 | SLC6A9 | SH3D19 | BCL3 | MME | MIR205HG |
| CEACAM7 | MUC1 | FUT3 | PTPRK | LOC283454 | LRIG1 | CLDN3 | PLA2G10 | CCL2 | SLAMF7 | KCNJ15 | DDR1 |
| VSTM2L | IFITM3 | CCDC88C | CCL7 | CCDC80 | C9orf89 | GPR125 | ANK3 | BAIAP2L1 | PRODH | PROC | MRVI1 |
| RGNEF | RBPMS | STYK1 | LOC440335 | SH3RF1 | RAB26 | TNFRSF8 | DYSF | FZD6 | ZNF462 | MYO5C | BNIPL |
| LAYN | EGR3 | SPINK5 | SLC7A2 | HS3ST1 | RHBDF1 | AJUBA | AATK | CLIC3 | HOMER3 | KCNMB4 | TMEM98 |
| KLK11 | SIX1 | MBOAT7 | PRR7 | CTSE | IGFBP3 | ATL1 | CLEC4G | ITGB4 | CXCR7 | GPA33 | RAB36 |
| SCIN | VGF | NEURL3 | B3GNT3 | TMCO2 | ENTPD3 | RASL11A | CHI3L1 | CCL4 | HEY1 | C1orf21 | PPP1R14C |
| OASL | SLC6A14 | TMEM61 | CD244 | MCTP2 | APCDD1 | RAB3IL1 | FAM3B | EFCAB1 | GPR84 | ITIH5 | ORM2 |
| NME5 | RIBC2 | DOK4 | WDR16 | IL18R1 | SLC25A4 | SLC35F2 | CDC42EP2 | IFITM4P | MARC1 | IER3 | DAPL1 |
| PLK3 | THBS1 | IGFBP2 | PRSS22 | CACNG6 | CLEC10A | GPR132 | ZNRF1 | PARVA | C8B | DNAH12 | KCNAB1 |
| CRYBB1 | VENTX | EPB41L4B | TMEM25 | CD5L | KCNS3 | LSAMP | RGS2 | AMIGO2 | LOC286058 | TSPAN8 | MLC1 |
| FHL1 | C5orf62 | CCR5 | TM4SF19 | ATP12A | IGFBP6 | AKAP14 | REEP2 | PLEKHA5 | MS4A8B | WIF1 | LOC100506328 |
| PLS1 | TMEM139 | EGFL6 | GNG2 | C3 | SPIRE2 | SLC2A5 | DENND3 | PPL | EPHX2 | ECE1 | FAIM3 |
| FLJ23152 | F5 | LOC388210 | ZNF469 | CCDC19 | INPP5A | CPE | ASAP2 | SOX2 | RNASE2 | SIGLEC8 | HBA2 |
| SNAI1 | RAB15 | HES4 | BPI | FERMT2 | MPO | MATK | SPP1 | CYP2J2 | LOC283485 | GZMK | PSCA |
| KRT6A | DNAH2 | DNAH5 | D4S234E | GZMB | ANO5 | LGALS3BP | LCP2 | RNASE3 | SH3BP4 | PLEKHG2 | SLC19A3 |
| SPRY2 | KRT17 | MAP3K14 | AUTS2 | HRK | LY6G6D | TINAGL1 | EFHB | PSTPIP1 | LIMD2 | C1orf106 | MACC1 |
| SALL2 | AXL | TTC18 | SLC44A3 | FOLR1 | HBB | FAM179A | NOTCH3 | ICAM1 | ADRB1 | GNAO1 | GJA1 |
| RASIP1 | LOC729860 | PRINS | NPNT | TSPAN19 | CRABP2 | SLC6A8 | C20orf46 | LOC400891 | C6orf165 | MSC | ADAMTSL3 |
| SCGB2A1 | C1orf173 | SGK1 | PAWR | ECSCR | C11orf88 | MYB | FOXJ1 | TNFRSF12A | CC2D2A | CMKLR1 | UCP3 |
| BTBD11 | LOC606724 | LDOC1 | EFCAB10 | ABCC6 | GPRC5C | NET1 | CASC1 | SETBP1 | C10orf10 | EMP1 | AWAT2 |
| CAT | AKAP2 | CRCT1 | LOC100134663 | CCDC74B | NANOS1 | CCL23 | ARHGAP20 | PZP | SLC43A1 | TGM3 | FLNB |
| SIK2 | CCR3 | SNN | LOC100134229 | MSR1 | DPP4 | MIAT | GPRIN2 | LOC645553 | MPZL2 | GATA1 | LOC283050 |
| SLC9A9 | LOC100129447 | C1orf194 | ENPP2 | SLC38A5 | PRKCZ | HAS1 | C20orf85 | ANGPTL4 | TLR10 | KPTN | INHBA |
| ARMC4 | PDCD1LG2 | MOCS3 | FAM81B | DYDC2 | C1QA | SHE | GDF15 | CGNL1 | DEFA4 | TEKT2 | RNASE1 |
| CD48 | DGKD | ORM1 | SPINK1 | STAT4 | LGMN | OLFM4 | TRAF3IP3 | CCDC75 | SAG | FLJ11235 | COLQ |
| LRRC10B | GAL3ST4 | C10orf107 | NTN1 | PEX11A | SLC39A8 | PBX4 | PECR | EPB41L4A | MAP2 | KRT5 | SRPX |
| OLIG2 | TTR | ASB18 | TNFSF15 | LOC643037 | HIST1H4H | LOC100129322 | HIST1H4E | LPCAT1 | NTS | ADAMTS7 | HILPDA |
| TIAM2 | FBN2 | PRF1 | AGAP1 | CCR7 | PGLYRP1 | AOC3 | NWD1 | TMEM95 | FCGBP | PTGER3 | KLRD1 |
| LAG3 | EPN3 | HIST1H4D | CAPSL | ATP10A | BEND5 | IDO2 | MYBPHL | PLXNC1 | SFTPD | CA8 | LOC100507637 |
| CD300E | TRPC2 | FAM20C | STAMBPL1 | CAPS | SPRR1B | CLC | CCDC37 | SGCE | SFTPC | PPIL6 | ATHL1 |
| RASAL2 | CHP2 | VCL | LOC100128607 | VNN3 | COL8A1 | PDE1B | DYNLRB2 | SLC26A11 | LOC115110 | ACOXL | TRPM2 |
| CHST9 | TRIM34 | SNX7 | CAMP | SPAG6 | SFTPA2 | CLSTN3 | PDE4D | EXD2 | DBR1 | PDCD1 | HIST1H4I |
| TNS4 | AMN | SYTL2 | TNFRSF11A | LOC283143 | SCG5 | FAM183B | PITPNM3 | PIM3 | WDR38 | PIK3R6 | PRM2 |
| RMRP | TAS1R3 | C15orf26 | FASLG | ABCC2 | CDCP1 | MS4A6E | IDO1 | RPPH1 | NUAK1 | SNORD3B-1 | ACVRL1 |
| GPR183 | LOC100288911 | GLTSCR1 | ZFP36 | MREG | PDPN | HOXB6 | TNFRSF25 | LOC100134240 | ZNF365 | STX19 | CRLF1 |
| PRRT4 | FN1 | ZNF831 | LOC145694 | CHAT | STXBP1 | PTPRE | OSBPL6 | C1QC | THBS4 | FAM157A | GNLY |
| IL1A | MOCOS | TLR9 | GEMIN5 | DLEC1 | PGA3 | ADORA3 | SCHIP1 | ZNF239 | CCDC146 | CYB5R2 | PTPRU |
| ProSAPiP1 | NAV2 | LOC100130298 | DUSP13 | TNFRSF18 | ALPK3 | GLTPD2 | SSTR3 | GPR126 | MORN3 | PTGER2 | C11orf70 |
| GZMA | RORC | TIMP3 | RNU12 | CDKN1C | ALDH1L1 | LRRC46 | AFAP1L1 | MB | KDR | OR2A2 | JAG2 |
| STAC | ODF3L1 | FAM26F | PPARG | RNF207 | C9orf116 | SCARNA7 | CXorf41 | MIOX | LDB3 | HIST2H4B | C16orf45 |
| FOXD1 | KCNN4 | C9orf24 | SPOCK2 | NPC1 | KRT4 | PSTPIP2 | ETV7 | ZFR2 | RTN1 | NRARP | MPP6 |
| SIT1 | CYP3A7 | MGAT3 | FANCD2 | PCOLCE2 | ARID5B | MSMB | AHNAK2 | PEG10 | MDH1B | C6orf118 | SLITRK4 |
| C15orf37 | ARHGAP6 | IGSF9 | RASGRP3 | ABP1 | C20orf123 | AKR1C4 | CRLF2 | AFAP1L2 | ARMC3 | FN3K | MMP1 |
| PARD6B | CCL5 | TIGIT | FLJ45248 | DIXDC1 | C7orf58 | DOK7 | TRAF1 | LEAP2 | CTNND2 | TMEM119 | JUP |
| SNORD17 | CHRM2 | KCNK7 | KGFLP2 | HAL | CHRNA10 | ENKUR | LOC100133991 | TMPRSS4 | C10orf105 | RTKN | CYTL1 |
| FAM3D | TCL1A | TGM2 | GRB10 | CTSK | PLA1A | FAP | RAB39B | CBFA2T3 | TXNDC3 | RGS1 | SLAIN1 |
| OSGIN1 | CLCN4 | C8orf47 | ITGA10 | NALCN | TMEM163 | LOC439949 | LRRIQ1 | C2orf73 | AMOTL1 | HIST1H4F | PTPDC1 |
| DAAM2 | STK17A | SMARCA1 | RETN | MICAL3 | SLC16A4 | ADH1A | COLEC12 | ISYNA1 | HPDL | PXDN | SNORD15A |
| MTL5 | SAA1 | SLC16A7 | MAP1LC3C | C4BPA | SH2D5 | OR2A9P | ATP8B1 | RAB27B | RRAS2 | GLS2 | PAQR5 |
| HBQ1 | C20orf202 | TBX21 | LOC728196 | ST18 | TMEM176A | PLXDC1 | SNORA81 | RGS16 | CD46 | DDHD1 | DHRS9 |
| ODF3L2 | SCARNA5 | FAM48B1 | IL32 | ITM2A | PLEKHF1 | LOC283392 | CACNA1C | KLHL29 | STOX1 | FGF13 | SCGB1A1 |
| ABCA1 | LAMC1 | STON1 | PNPLA3 | MIA | psiTPTE22 | FSCN3 | ICOS | IL2RA | LYPD5 | COL21A1 | ATP7A |
| HIST1H2AL | SH2D4B | MST1R | HP | RGS9 | C5orf49 | HIST1H1B | KRT1 | PNMT | ALK | MS4A1 | HIF3A |
| DFNA5 | IGJ | MTMR11 | GPR176 | DGAT2 | ADAMTS10 | CEACAM21 | HYDIN | HPR | DDAH1 | ITGA9 | DENND1B |
| IL1R1 | AQP10 | WTIP | ETNK2 | CCDC17 | LOC152286 | KIT | CNR1 | OR2M5 | CITED4 | VANGL2 | OR13C4 |
| TBPL2 | DEFB126 | LRFN5 | SOX7 | SLC48A1 | BBC3 | LOC541472 | RHBDL2 | REPS2 | NMNAT2 | PLEKHG1 | HTR2A |
| BLVRB | CBLC | LOC100129098 | LOC401442 | TNIP1 | PP14571 | LOC100129280 | KLHDC8A | BAHD1 | GLIS2 | SNORA55 | SGPP1 |
| EMX2OS | LOC100130899 | FAM133A | NPFFR2 | CCDC102B | PCDH17 | DNER | DGKI | PKD2L2 | HAO1 | FLJ27502 | CDH8 |
| LOC100129250 | TFDP2 | PLSCR3 | TSPAN31 | LOC284648 | PAMR1 | LOC100130152 | PLCZ1 | PLCB4 | CLLU1 | SDPR | EGOT |
| ABHD1 | KCNJ11 | RHBDD3 | DMRTB1 | CXorf58 | KCNK12 | CIR1 | C8orf4 | TMEM50B | ZDHHC18 | VIPR1 | SERTAD4 |
| RASSF9 | CUEDC2 | PAGE1 | KIR3DL1 | ASB5 | PYY | PLEKHB1 | AMELX | GPX8 | HS3ST5 | TRPC5 | MYOC |
| OR4A16 | KLHL14 | ABCC11 | LOC645188 | LOC285768 | PNMA6C | ZDHHC16 | GLB1L3 | OR1A1 | ABCD2 | CFTR | HOXD3 |
| C7orf55 | LOC100132707 | DDX31 | WFDC6 | CCDC132 | LOC100132891 | MAGI3 | NUCKS1 | ARPM1 | RGS14 | LOC440297 | LOC100128402 |
| C6orf225 | APOL6 | SLC24A4 | ZNF518A | FAM107A | STX3 | MUC13 | FAM167A | IL12A | FLJ32063 | LOC157503 | MUC20 |
| C9orf68 | DNAJB5 | C20orf26 | C19orf20 | HNRNPR | C21orf116 | CNGA3 | MGC4859 | CD320 | CCDC129 | AKNA | TNC |
| HPN | MS4A15 | ITGB1BP3 | SKAP1 | TMPO | TMPRSS2 | RGPD6 | GPRIN3 | PEX11G | PTPRCAP | FEZF1 | RUNX2 |
| GATA6 | PKIA | DSC2 | CDON | MYH4 | C9orf150 | ZNF324B | IL21R | NAP1L2 | LOC100132354 | OR13H1 | LOC100129869 |
| TAC4 | ZNF658 | ANKMY2 | PARP6 | SH3BGRL2 | REEP6 | OR6V1 | POU2F3 | SLC35A5 | RAB2B | MAPK13 | RCAN2 |
| PLEKHH1 | WDR65 | SLN | IFT20 | LOC401098 | CDKL1 | PRG1 | TCF4 | IFNE | PBOV1 | EBF3 | FUT9 |
| SYT3 | CENPT | ZNF580 | STXBP4 | RAB19 | KDM1B | AK7 | VTN | THOC1 | CELA1 | PDE6G | OSTM1 |
| FIGLA | SAMD13 | PCDHB19P | PAGE2 | LAMA4 | LOC400043 | FAM46D | SKI | SLC26A9 | LOC100129387 | CDKL2 | ROCK2 |
| ZNF614 | FCGR2B | TMEM27 | UBD | MAP3K15 | RNF43 | LOC338739 | C2orf65 | POLR1E | PPM1H | G6PC2 | KIAA1239 |
| ACSM3 | OR1Q1 | GSDMA | INPP5F | SECISBP2L | C4orf47 | C21orf128 | LOC729187 | AURKC | RND3 | IKZF5 | PPP2R3A |
| OR10H2 | ZSCAN4 | LOC100128054 | IL7 | LOC100129775 | BRE | LOC441601 | FBXO10 | C12orf61 | DACT3 | CPXM1 | GNAT2 |
| OR4C3 | ZMYND10 | TBC1D22A | PDZD2 | LOC100505806 | C9orf91 | POLR2C | RGMA | PKHD1L1 | LOC401052 | KIF9 | HPCA |
| LMBR1 | PNMA2 | KLK4 | RNMT | LRRIQ3 | AVPR1A | FAM113A | FAIM2 | LOC100128292 | GRTP1 | SNTA1 | LOC100506533 |
| FKBP11 | HHIPL1 | NDUFA4L2 | HSPA12A | DDX25 | SPP2 | SRPK3 | ABCB4 | EDIL3 | IVL | C14orf109 | SSH1 |
| KCNQ3 | COG5 | PKP2 | PTBP2 | LOC440925 | PRM3 | SIX2 | SLC9A1 | ZNF699 | JOSD2 | LRRC49 | BACH2 |
| ALDH1B1 | CLDN14 | IMMP1L | ZDHHC13 | TTC39B | MTUS2 | CPLX4 | ZNF99 | LDLRAD2 | SYNM | TAS2R42 | KRT75 |
| NPHS2 | NELL1 | KHDRBS3 | ZNF542 | TEAD4 | MMP19 | DTX3L | FGF19 | SERPINF2 | NT5C3 | ASCL2 | MFSD9 |
| TMPRSS11E | C17orf108 | TTC4 | PAPOLB | LOC149134 | ACBD5 | IFRD1 | ENOX1 | B3GNT9 | LOC400662 | SLC16A8 | CEP76 |
| MGC3771 | LOC100270746 | LOC157740 | LOC151484 | LOC100130278 | FKBPL | UBXN10 | PAX1 | SAP30 | PCLO | TAAR5 | LUZP2 |
| ST3GAL5 | HCFC2 | PAFAH1B2 | TMEM14E | TBC1D21 | PILRB | GATC | ZIC3 | SNORA74B | LOC401022 | DSG4 | AFAP1 |
| DHFR | OR4K1 | TBL1Y | FIBCD1 | POLQ | C21orf67 | GAFA2 | ADARB2 | CYP2U1 | SMTNL2 | WFDC10A | SLC26A2 |
| RNF144A | FAM117B | ISY1 | IL20RA | PHOSPHO1 | ST5 | ZNF416 | FAIM | VWA3B | TMC2 | TMCO7 | RBMXL1 |
| MAGEE1 | EMILIN1 | HEATR7B1 | ZFP82 | BCAN | TTC8 | LOC401188 | C16orf53 | DNAJC5G | MMP15 | LOC286189 | ZBTB44 |
| LOC100129113 | BEND4 | CCDC125 | C3orf65 | HLCS | LOC283475 | FLRT3 | P2RY12 | ELANE | MUC5AC | PRO2012 | PDK3 |
| C8A | MAGEB5 | DUOXA2 | RPE | OR2W5 | RTKN2 | PROM1 | KCTD10 | HIST1H2AI | CYP17A1 | PLAC2 | LOC729059 |
| LOC100130256 | CDH5 | VWDE | C6orf64 | FAM63B | SYT17 | STBD1 | TMEM106B | ANO2 | LIN7B | ERMAP | KANK3 |
| C20orf144 | FLJ45983 | LOC284440 | NLRP8 | RAB38 | EXTL2 | IL17RE | PIAS1 | C20orf118 | FAM135A | SLC4A8 | KDM1A |
| GABRR1 | PPP1R15A | PIGZ | TBC1D8 | SEMA3D | CCIN | TMEM133 | KCNK4 | FARSB | LOC100131490 | RBM15 | ATP8B3 |
| CDC42EP4 | GAS2 | SUPT3H | TRIM61 | OR8H2 | NFKB2 | BAX | RSBN1 | KCNB2 | LOC100130417 | CCR10 | C9orf135 |
| SLC39A9 | APC2 | TYW1B | C7orf62 | TLK2 | TSN | GJB3 | KCTD1 | GIPR | BRWD1 | NPB | PARP15 |
| CNDP1 | C5orf24 | BET1 | WDR44 | MAP1S | ZG16 | ARMCX2 | NLRP12 | OMA1 | RORA | CDK5R2 | PTCHD2 |
| ALDH1A2 | HEXIM1 | ROPN1L | ARG2 | PIP5K1A | C1orf51 | LOC100131894 | HSPB9 | FLRT2 | FLJ13744 | NFIA | FLJ37644 |
| PRKACB | SLC15A2 | TRO | NQO1 | FGG | CNGA2 | OPHN1 | TSPYL5 | NUP188 | LOC400743 | SPRYD4 | LOC550112 |
| HNF1B | FLJ39095 | NOX1 | MKRN3 | KIAA1683 | CR1L | IL27 | CYP4F22 | FRMD6 | LIAS | LONRF2 | HMMR |
| FANCB | BARX2 | KHK | ALG9 | GJB6 | OR1E1 | LIN9 | LOC100129572 | ZNF155 | CCDC13 | LOC729799 | CCNC |
| CEACAM8 | RPRD1A | PHF8 | OR10K2 | TTLL7 | SPDYE4 | OR6C70 | PDZD8 | PKN1 | GOLGA6L7P | LOC283585 | DUSP21 |
| STAM2 | ADCK2 | MGC12916 | COX6A2 | SLC30A8 | PRAMEF6 | TMEM150B | DOCK1 | OTOR | FUT5 | PRRT2 | CNOT7 |
| SMU1 | FOXE3 | LOC100130950 | TMEM213 | TUBB1 | SBDS | TRMT12 | OR7A17 | ASMT | ISLR | GP2 | FMOD |
| PHACTR3 | FAM24A | C11orf94 | DNAJC3 | FAM194A | LOC93432 | CAPN6 | TMED8 | ATP6V0D2 | ALPK1 | ZNF76 | TAC1 |
| LOC730159 | PIGM | LOC643802 | CENPJ | FLJ37798 | SRD5A3 | TIMM22 | STK3 | ITGB8 | RAB11FIP2 | CRISPLD2 | DYX1C1 |
| COL7A1 | KCNA5 | ATP6V0D1 | LOC100127983 | GNB3 | LOC100134259 | EVI2B | ADAMTS3 | LYG1 | FAM83C | ZNF274 | DHX40 |
| LRRC29 | CYYR1 | LOC647946 | EFHA2 | PITX1 | ACAP2 | LILRA4 | PRL | IGFL2 | DIRAS1 | PPT2 | CBY1 |
| COL12A1 | TTC26 | GLS | KCNG4 | APBA1 | KCNA3 | TLR6 | ARIH1 | IRG1 | TMOD2 | LOC285972 | LEFTY1 |
| ZNF250 | PANX3 | LOC340515 | LOC644554 | TMEM19 | UCHL5 | CAMTA1 | AHRR | LOC283738 | CACNA2D1 | KIAA0586 | S1PR5 |
| SNORA22 | TYW3 | CHRDL2 | C15orf38 | TTC16 | C10orf114 | PCOLCE | IL7R | TMTC1 | THRB | FAM186A | GCNT3 |
| C4orf19 | ZNF219 | C11orf74 | JMJD7 | LOC643441 | BLZF1 | NBR2 | LOC100128304 | FLJ10038 | SLC30A7 | CENPQ | C12orf48 |
| ADCY6 | SEMA5A | PCDHB4 | ZNF92 | ABCG2 | NKIRAS1 | RBM41 | TSC22D4 | NUP210L | KRTAP22-2 | HTR6 | ZNF415 |
| FEM1A | OR2B6 | PGM2L1 | OR51B6 | CBFA2T2 | NTN5 | RNU11 | ENTPD2 | PLAC8 | GALNT10 | TRUB1 | FAM46B |
| UGT8 | SNTB1 | PIGK | SLC29A2 | FAM155B | CNPY4 | C16orf87 | MRPL42P5 | UOX | SRPR | GRIN2D | DCUN1D4 |
| TTC21A | CLDN24 | ESF1 | LOC339400 | SRRM2 | PASK | LPIN1 | HIPK3 | CLSPN | ZNF295 | STK35 | SORCS3 |
| RDH8 | LOC100132344 | LRRCC1 | HMSD | PADI3 | SULT2B1 | DSCR9 | KRTAP7-1 | ARTN | OSBPL10 | SOX3 | VN1R3 |
| ITGA2B | NOVA2 | NEDD1 | H2BFM | ZFP112 | LPP | GOLGA5 | TOM1L2 | SENP6 | TOMM70A | FTSJD1 | ASCC3 |
| QPCTL | ZNF571 | GPATCH2 | ERO1LB | ZNF594 | TTLL11 | LOC145945 | GSTT2 | PLAC8L1 | GORASP1 | C14orf177 | HHEX |
| PLCG1 | PGBD5 | C11orf45 | KRTAP10-3 | STRN | C17orf99 | LUM | CLDN1 | TTC33 | FAM111B | STRA6 | C8orf83 |
| ZNF420 | ZFP28 | FAM90A1 | PCDHGC5 | CCL28 | RHEB | AGBL3 | SLC25A36 | TCF15 | DKFZP564C196 | SH2D2A | SORBS1 |
| RAB40B | FAM70A | KNTC1 | BAZ1B | ARMC2 | LRP11 | IQCE | PIK3C2A | VPS41 | C2orf3 | ISM1 | OR5AK4P |
| KIR3DL3 | FBXL7 | LMBRD2 | DMRT2 | PM20D1 | GANC | ZNF205 | CLPTM1 | MSX2P1 | HOXC4 | DEFB106B | PRICKLE1 |
| KCNH4 | RNF219 | FLJ14107 | TDRD9 | AKR1E2 | OR56A4 | PPY | CLEC17A | ERRFI1 | PRPS2 | TRDN | EIF2C3 |
| SPATA20 | RABL3 | HOXA4 | C19orf39 | C22orf43 | LOC100506342 | EPC1 | DET1 | SPINK8 | SHROOM2 | SMEK1 | RASSF4 |
| CRYBA4 | RASD2 | ZSCAN1 | ASAP1 | C6orf97 | PPP1R2P3 | SPRED2 | SLC30A5 | TRIM59 | OR5F1 | CD3E | GCC1 |
| NUDT12 | KIAA1328 | FAM150B | LCE1C | ANKRD6 | CSTF3 | TTTY2 | ZNF846 | STRA8 | PRUNE2 | C4orf51 | DDI2 |
| GRIN2C | TMEM33 | GIPC3 | FAM154A | CATSPER3 | NRSN1 | MCM9 | CD226 | GPR21 | CLCN7 | MAGEA12 | CRIPT |
| COX7A1 | ZBTB38 | MAN2A1 | TBX18 | LOC150568 | C10orf40 | DNAJC25 | OR8G2 | SESN1 | LOC148145 | ZNF490 | C1orf88 |
| GSX1 | SRM | TAOK1 | SCGB3A1 | C12orf53 | CASC5 | LOC100287326 | CTGF | KLHL15 | C15orf32 | IRAK3 | C8orf51 |
| DZIP1 | OPALIN | SLC6A18 | CENPA | ZNF564 | RHBDL1 | LOC100130560 | PIK3R3 | DPRX | WNT6 | VIPR2 | TAAR8 |
| KLRC1 | TPM2 | HEATR5B | UNC5CL | SLC4A5 | KIAA1715 | ZW10 | ZBTB33 | FAM190A | ZNF808 | SCO2 | STT3B |
| MSRB3 | RSRC2 | LOC729732 | LOC100129826 | GALNT1 | FGF11 | FAM90A7 | PCDHGB4 | FLT3LG | CD248 | FAM82A1 | ARHGAP22 |
| LOC100133920 | SLC25A30 | C11orf63 | SATB2 | CST1 | GOLGA6A | MINA | TPR | C21orf58 | PALLD | OLIG3 | STK17B |
| RASA1 | LOC100128170 | DUS4L | C8orf66 | ZCCHC18 | MEG8 | TMEM38B | KLHL28 | OR4D11 | KIF11 | TRIM10 | MFAP4 |
| OR5D13 | ALS2CL | FLJ38109 | LOC100132966 | LCMT2 | PGBD4 | SKA1 | ACSS3 | C4orf43 | CBX2 | PIK3IP1 | PTH2R |
| CTH | FAM8A1 | SGCB | ERG | PRDM15 | CXorf22 | CD22 | HOXD13 | CCDC71 | KIR2DS2 | TPD52L1 | ZNF879 |
| CACHD1 | NEK8 | ZNF84 | LOC284757 | LOC729696 | GKN1 | LOC728190 | ZNF555 | SERPINB13 | WDFY1 | COL9A2 | ZNF843 |
| OR56B1 | KALRN | SSTR4 | TUBGCP3 | ZNF93 | RECQL5 | CYP4X1 | C9orf173 | CDKN1A | KRTAP19-7 | LOC57399 | ALDH5A1 |
| SNORA53 | POLR2M | ZNF300 | RUFY2 | TAF1A | DPPA5 | UTP15 | OVGP1 | LOC283887 | KRTAP5-9 | SLC1A2 | KRTAP4-7 |
| KLHL7 | PMS1 | PRKAB1 | FKSG29 | TBC1D16 | FLJ45482 | RANBP17 | GLIS3 | CD1A | MAPT | C14orf79 | FAM133B |
| KIAA1704 | CCK | ZNF292 | DPRXP4 | LOC283624 | CCR4 | ZNF23 | DKFZp761E198 | PIAS2 | EML5 | GPR112 | PRPF38A |
| VPS13A | KRTAP4-8 | FEM1B | NCALD | WDR88 | LOC147004 | FOXN2 | SLC39A10 | SFI1 | BLK | FILIP1L | USH1G |
| DEPDC1 | PALMD | PLEKHF2 | ATAD3C | ICAM4 | ZNF841 | HES5 | KLHL32 | TP53AIP1 | CTDSPL | GFOD1 | OTOS |
| SHCBP1 | FNDC7 | OR1N2 | DNAJC6 | P4HA2 | OTUD6A | PLEK2 | NPAT | PBK | CATSPER2 | ZFHX3 | GRHL2 |
| TMPRSS3 | SLC8A2 | GOLGA6L9 | AURKAPS1 | ZNF706 | PRO0611 | TUSC1 | HCG27 | ZNF703 | WDFY2 | GPHA2 | GTF2F2 |
